# Polymeric Nanocarriers and Polymer-Assisted Delivery Platforms for Oleanolic Acid: Design Strategies, Controlled Release, Translational Challenges, and Clinical Perspectives

**DOI:** 10.3390/mi17080944

**Published:** 2026-08-07

**Authors:** Andrzej Günther, Barbara Bednarczyk-Cwynar

**Affiliations:** 1 Department of Organic Chemistry, Faculty of Pharmacy, Poznan University of Medical Sciences, Collegium Pharmaceuticum 2 (CP.2), Rokietnicka Str. 3, 60-806 Poznan, Poland; bcwynar@ump.edu.pl; 2 Center of Innovative Pharmaceutical Technology (CITF), Rokietnicka Str. 3, 60-806 Poznan, Poland

**Keywords:** oleanolic acid, polymeric nanocarriers, PLGA nanoparticles, polymer-assisted delivery platforms, controlled release, hydrogels, microneedles, local delivery, wound healing, psoriasis

## Abstract

Oleanolic acid is a naturally occurring pentacyclic triterpenoid with broad preclinical promise in inflammation, oxidative stress, liver injury, metabolic disorders, cancer-related models, skin disease, and wound repair. Its further development, however, is constrained by poor aqueous solubility, low and variable bioavailability, limited barrier transport, crystallinity, and strong dependence of biological response on the formulation used. These properties make oleanolic acid a useful example of a hydrophobic natural compound whose pharmacological performance is inseparable from delivery design. This review examines polymeric nanocarriers and polymer-assisted delivery platforms developed for oleanolic acid delivery. Polymeric nanocarriers discussed in the review include biodegradable PLA/PLGA nanoparticles, PEGylated polymeric nanoparticles, polymeric micelles, nanogels, hyaluronic-acid-based nanoprodrugs, and selected polymer-assisted hybrid nanostructures. Hydrogels, polymeric fiber membranes, local depots, and microneedle systems are included as route-enabling delivery platforms when the polymeric matrix directly contributes to OA incorporation, carrier stabilization, local retention, barrier bypass, or release control. Non-polymeric delivery systems are discussed only as comparators or when their performance depends on integration with a polymeric component. Rather than treating these carriers only as solubility enhancers, the review evaluates how polymer composition, carrier architecture, drug physical state, release behavior, and route of administration affect oleanolic acid exposure. Particular attention is given to controlled release, local retention, disease-oriented delivery, and critical quality attributes such as particle size, loading, encapsulation efficiency, solid-state form, stability, residual solvent, sterility, and batch-to-batch reproducibility. Representative quantitative data on carrier size, drug loading, encapsulation efficiency, release, stability, tissue exposure, and biological outcomes are compared to illustrate both formulation-specific performance and the substantial methodological heterogeneity of the available studies. The available evidence indicates that increased apparent solubility, increased biological exposure, and improved therapeutic response should be treated as related but distinct outcomes. The most realistic near-term opportunities may lie in local and tissue-targeted applications, including inflammatory skin disease, wound healing, dermal delivery, and osteoarthritis, where sustained target-site exposure may be more relevant than systemic bioavailability. Future progress will depend on demonstrating that each formulation provides reproducible, safe, and route-appropriate OA exposure, together with a measurable advantage over simpler delivery approaches.

## 1. Introduction

**Figure 1:**
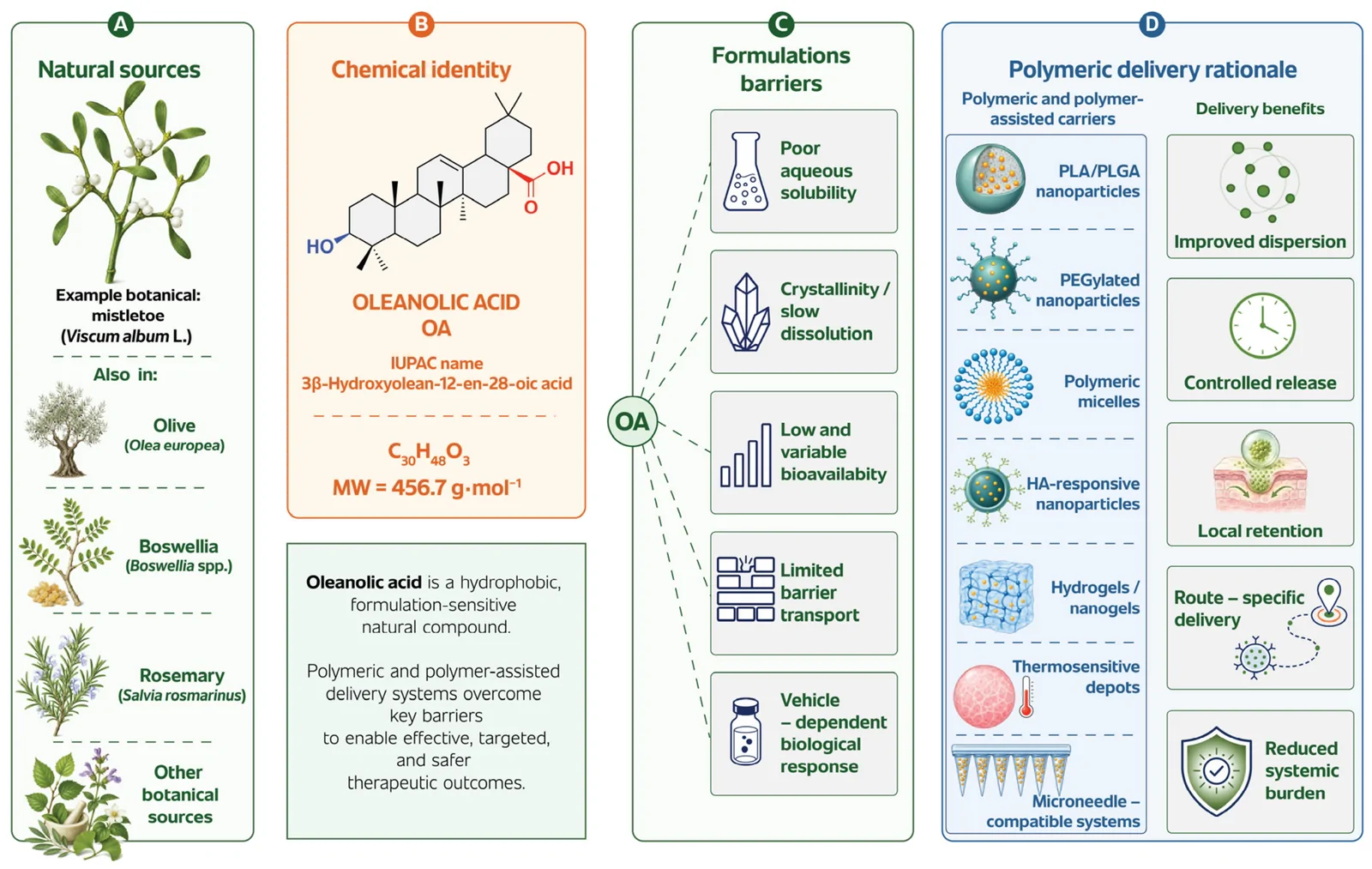
Oleanolic acid as a formulation-sensitive payload for polymeric and polymer-assisted delivery. Oleanolic acid (OA) is a naturally occurring pentacyclic triterpenoid found in multiple botanical sources, including triterpene-rich medicinal plants such as mistletoe (*Viscum album* L.). Its rigid hydrophobic oleanane scaffold, limited polar functionality, poor aqueous solubility, crystallinity, low and variable bioavailability, limited barrier transport, and vehicle-dependent biological response make OA a formulation-sensitive compound. Polymeric nanocarriers and polymer-assisted delivery platforms, including PLA/PLGA nanoparticles, PEGylated nanoparticles, polymeric micelles, hyaluronic-acid-based responsive carriers, hydrogels, nanogels, local depots, and microneedle-compatible systems, can be designed to improve OA dispersion, controlled release, local retention, and clinically relevant exposure.

Oleanolic acid (OA) is a naturally occurring pentacyclic triterpenoid present in edible plants, medicinal herbs, olive-derived materials, fruit peels, and other botanical sources [1]. Chemically, it is described as 3β-hydroxy-olean-12-en-28-oic acid, with the molecular formula C_30_H_48_O_3_ and a molecular weight of approximately 456.7 g/mol [2,3,4]. Although OA has attracted broad pharmacological interest, its formulation behavior is difficult: the rigid hydrophobic oleanane scaffold and limited polar functionality result in poor aqueous solubility, slow dissolution, and variable biological exposure [1,5]. These properties make OA less a conventional small-molecule payload and more a formulation-sensitive compound whose apparent activity depends strongly on how it is dispersed, released, and delivered. The natural occurrence, chemical identity, and formulation-relevant limitations of OA are summarized in Figure 1.

The biological literature on OA covers anti-inflammatory, antioxidant, hepatoprotective, metabolic, anticancer, antimicrobial, dermatological, and wound-healing-related effects. These reports are promising, but they are not interchangeable. The measured response depends on dose, route of administration, model, vehicle, and formulation type [1,5]. For a poorly soluble compound, the amount added to an in vitro assay or administered in vivo may differ substantially from the fraction that is actually dissolved, released, absorbed, or retained at the biological target [5].

For this reason, formulation is not a secondary technical detail in OA research. Poor aqueous solubility can limit dissolution and absorption, whereas high lipophilicity can promote partitioning into membranes, proteins, lipid compartments, or delivery matrices [1,5]. Solvents, surfactants, cyclodextrins, emulsions, nanoparticles, and polymeric carriers may therefore change not only OA solubility, but also exposure, cellular uptake, release kinetics, and apparent potency [5,6].

A wide range of OA formulations has been reported, including solid dispersions, cyclodextrin complexes, phospholipid complexes, self-emulsifying systems, liposomes, lipid nanoparticles, polymeric nanoparticles, albumin-based carriers, hydrogels, and local delivery platforms [5,6,7,8,9]. Together, these studies show that OA can be incorporated into many carrier types. They also show that carrier composition, preparation method, physicochemical characterization, and biological model strongly affect how the results should be interpreted [5,9,10]. These formulation strategies are not functionally equivalent. Cyclodextrin complexes primarily improve aqueous compatibility through inclusion complexation, whereas self-emulsifying systems maintain OA in a solubilized or lipid-associated state during gastrointestinal dispersion [5,11,12,13]. Liposomes and other lipid-based carriers provide hydrophobic domains compatible with OA, but may offer limited control over drug leakage, storage-related redistribution, or release after dilution [5,14,15]. Polymeric systems are emphasized in this review not because they are universally superior to these alternatives, but because they can combine hydrophobic drug accommodation with matrix-mediated release, surface modification, local retention, stimuli-responsive behavior, and integration with route-enabling dosage forms [7,8,9,12,13,15,16]. Simpler solubilization systems may remain preferable when prolonged release, tissue retention, barrier bypass, or carrier-mediated biological interaction is not required.

Polymeric nanocarriers and polymer-assisted delivery platforms are useful in this context because they can address several OA delivery problems simultaneously. Biodegradable polymers such as PLA and PLGA can accommodate hydrophobic OA within nanoparticle or fiber matrices and modulate release through diffusion, relaxation, erosion, or degradation [7,8,9,11,14]. PEGylated systems can improve colloidal stability and alter the biological interface of the carrier [7]. Hyaluronic-acid-based nanoprodrugs add another level of design by combining polymer–drug conjugation, disease-oriented interactions, and stimuli-responsive behavior, as shown in topical psoriasis models [12].

These systems are also relevant for local and transdermal delivery. Many plausible OA applications involve inflammation, skin pathology, tissue repair, osteoarthritis, or localized oxidative stress, where high systemic bioavailability may be less important than controlled exposure at the target site [1,5,8,12]. Polymeric fiber membranes, hydrogels, nanogels, dissolving microneedles, and hydrogel-forming microneedles can therefore be used not only to increase apparent solubility, but also to prolong local retention and reduce unnecessary systemic distribution [6,8,13].

For polymeric OA formulations, the key questions go beyond whether the compound can be loaded into a carrier. A useful formulation must define and control critical quality attributes such as particle size, size distribution, drug loading, encapsulation efficiency, polymer degradation, drug physical state, release profile, residual solvent, colloidal stability, and batch-to-batch reproducibility [6,17]. These parameters determine whether an OA-loaded system can provide reproducible exposure under conditions relevant to the intended route of administration [5,6,13].

This review focuses on two related but distinct groups of OA delivery systems. The first comprises polymeric nanocarriers in which the principal nanoscale architecture is formed by a polymer, including PLA/PLGA nanoparticles, PEGylated polymeric nanoparticles, polymeric micelles, nanogels, and polymer–drug nanoprodrugs. The second comprises polymer-assisted delivery platforms, including hydrogels, fiber membranes, thermosensitive depots, and microneedle systems, in which a polymeric matrix stabilizes an OA-containing carrier, controls local residence and release, or enables administration across a biological barrier [7,8,9,12,14,15,16,18].

The inclusion of these larger platforms is therefore based on function rather than size alone. They are discussed when the polymeric component directly determines OA incorporation, physical-state control, release kinetics, tissue contact, local retention, or barrier bypass. Purely non-polymeric systems are considered selectively as comparators or when they clarify the relative advantages and limitations of polymeric design [5,17,19,20,21,22].

The review does not aim to summarize all reported pharmacological effects of OA or all possible OA formulations. Instead, it asks a formulation-centered question: under which conditions do polymeric nanocarriers or polymer-assisted delivery platforms provide a meaningful advantage over simpler solubilization approaches in terms of release control, tissue exposure, route suitability, reproducibility, or translational feasibility [5,7,8,9,11,12,13,14,15,16]?

## 2. Literature Search Strategy and Review Methodology

This review was prepared as a critical narrative review supported by structured literature mapping. The aim was not to catalogue all reported pharmacological effects of oleanolic acid, but to evaluate how polymeric and polymer-assisted delivery systems may address the formulation barriers that limit its biomedical use [1,5].

The search was organized around four problem-oriented areas: physicochemical and biopharmaceutical limitations of oleanolic acid; polymeric and polymer-assisted carrier systems reported for oleanolic acid; controlled-release and local-delivery strategies; and translational issues related to characterization, manufacturing, safety, and disease relevance [1,5,7,9,11]. Relevant studies were identified through PubMed, Scopus, Web of Science, ScienceDirect, SpringerLink, Wiley Online Library, ACS Publications, MDPI, Taylor & Francis, RSC Publishing, and Google Scholar.

The main search terms combined “oleanolic acid” with “polymeric nanoparticles”, “PLGA”, “PLA”, “PEGylated nanoparticles”, “polymeric micelles”, “nanogels”, “hydrogels”, “hyaluronic acid”, “cyclodextrin”, “polymer-lipid hybrid nanoparticles”, “controlled release”, “transdermal delivery”, “microneedles”, “wound dressing”, “psoriasis”, “osteoarthritis”, “cancer nanomedicine”, and “oral bioavailability”. Priority was given to peer-reviewed articles published from 2000 onward, with particular attention to studies published after 2015 because they better reflect current developments in polymeric nanomedicine, stimuli-responsive carriers, hydrogels, microneedles, and translational nanocarrier design [5,7,8,9,11,12].

Studies were included when they addressed at least one of the following points: OA physicochemical or biopharmaceutical limitations; formulation of OA in polymeric nanocarriers; polymer-assisted stabilization or release control; local, dermal, transdermal, intra-articular, or microneedle-assisted administration; biological evaluation of OA-loaded carriers; or translational aspects relevant to polymeric drug delivery [5,7,8,9,11,12,15,16,18].

Macroscopic polymeric platforms, including hydrogels, fiber membranes, wound dressings, local depots, and microneedles, were included only when the polymeric structure directly contributed to OA incorporation, stabilization of an embedded nanocarrier, controlled release, tissue retention, mechanical administration, or biological-barrier bypass. Studies focused only on botanical extraction, phytochemical identification, or general OA pharmacology were used only when they provided necessary background. Purely non-polymeric systems were discussed selectively, primarily as comparators or when a polymeric component contributed to PEGylation, surface stabilization, gelation, matrix formation, or route-specific delivery [1,5,7,11,18].

The review is structured from formulation barriers to delivery design and translation. First, the intrinsic limitations of OA are defined. Next, polymeric and polymer-assisted carrier types are compared. Controlled-release, local-delivery, and microneedle-compatible strategies are then discussed. The final sections consider characterization, critical quality attributes, manufacturing, safety, clinical relevance, and future development priorities [5,6,9,13].

Where quantitative data were available, representative values for carrier size, polydispersity, surface charge, drug loading, encapsulation efficiency, release, stability, tissue exposure, and biological response were extracted from the original studies. Because the reported systems differed in formulation composition, analytical methods, release conditions, doses, and experimental models, these values were synthesized descriptively rather than subjected to formal meta-analysis.

## 3. Physicochemical and Biopharmaceutical Barriers in Oleanolic Acid Delivery

### 3.1. Chemical Identity and Structural Implications

The formulation behavior of OA follows directly from its structure. The molecule contains a rigid pentacyclic oleanane scaffold, one hydroxyl group, and one carboxylic acid group. This combination gives OA a predominantly lipophilic character with only limited capacity for interaction with aqueous media [1,2,3]. As a result, OA is more compatible with hydrophobic or amphiphilic domains than with simple aqueous formulations.

For polymeric delivery, this structural profile is important. OA can partition into hydrophobic polymer matrices, micellar cores, lipid–polymer interfaces, nanogel domains, or other microenvironments that reduce direct contact with water [5,7,9,11].

Biodegradable polymers such as PLA and PLGA are therefore attractive not only because they form nanoparticles, but because their hydrophobic domains can accommodate a compound that is poorly suited to aqueous dispersion. Amphiphilic and PEGylated polymers add a further function by combining hydrophobic drug association with improved colloidal stability in biological media [7,9,13].

The same structural features also create risks. Strong drug–matrix association may slow release, whereas insufficient compatibility between OA and the carrier can lead to crystallization, surface deposition, or precipitation during preparation and dilution [1,5,7,9]. Polymer selection should therefore be guided not only by biodegradability or particle size, but also by polymer–drug compatibility, physical-state control, and the intended route of administration [5,7,9,13].

### 3.2. Poor Aqueous Solubility as the Primary Formulation Barrier

Poor aqueous solubility remains the central formulation barrier for OA [1,5]. It limits dissolution in gastrointestinal fluids, complicates preparation of parenteral or topical dosage forms, and reduces the reliability of biological testing when nominal concentration is assumed to represent actual exposure [1,5,17]. In practice, OA added to an aqueous assay may exist as dissolved drug, suspended particles, precipitated material, carrier-associated drug, or a mixture of these states.

For polymeric nanocarrier design, poor solubility is both the problem and the rationale. OA may crystallize, precipitate, or remain incompletely incorporated during formulation. At the same time, polymeric nanoparticles, micelles, nanogels, and hydrogels can provide microenvironments in which OA is dispersed, protected, or released gradually rather than presented as a poorly soluble crystalline solid [5,7,9,12].

This distinction matters when biological effects are compared across studies. Cyclodextrin complexation, PEGylated PLA/PLGA nanoparticles, PLGA nanoparticles, micelles, gels, and other carriers do not simply “increase solubility”; they change the form in which OA reaches cells or tissues [7,9,17]. For this reason, OA should be treated as a formulation-dependent compound. Differences in activity between “free” OA and carrier-loaded OA may reflect changes in dissolution, precipitation, uptake, release rate, or tissue deposition rather than a change in intrinsic pharmacological potency [5,7,17].

### 3.3. Crystallinity, Dissolution, and Physical-State Control

OA is generally handled as a poorly soluble crystalline or semi-crystalline solid, and its solid-state properties contribute to slow dissolution and variable biological availability [1,5]. In polymeric nanocarriers and polymer-assisted delivery platforms, OA may be molecularly dispersed, amorphous, present as crystalline domains, adsorbed on the carrier surface, or partially phase-separated from the carrier. These states are not interchangeable because they can produce different release profiles, storage stability, precipitation behavior, and biological exposure even when particle size and encapsulation efficiency are similar [5,7,8,9,13].

Molecularly dispersed OA is distributed within the carrier at the molecular level. This state can support relatively uniform drug distribution and more reproducible release because dissolution of separate OA crystals is not required before transfer into the surrounding medium. However, molecular dispersion does not guarantee long-term physical stability. Changes in temperature, humidity, polymer mobility, solvent content, or drug concentration during storage may promote nucleation and recrystallization [5,8,9,13].

Amorphous OA lacks the ordered lattice structure of the crystalline drug and may therefore show faster apparent dissolution or release. Its higher free energy can be advantageous for a poorly soluble compound, but it also increases the risk of physical instability. Amorphous OA may recrystallize during drying, storage, dilution, polymer degradation, or exposure to biological fluids, leading to changes in release rate and batch-to-batch performance [5,8,9].

Crystalline OA domains within or outside the carrier generally require dissolution before the drug becomes available. Depending on crystal size, location, and interaction with the polymer matrix, this may result in slow, incomplete, or highly variable release. Undetected crystalline fractions may also produce misleadingly high apparent drug loading if crystalline material remains associated with the formulation after purification [5,7,8,9].

Surface-associated OA has a different risk profile. Drug deposited or adsorbed on the carrier surface may produce an initial burst release, followed by precipitation after dilution if the surrounding medium cannot maintain OA in a dispersed state. Surface-associated OA can also complicate the interpretation of encapsulation efficiency unless free, precipitated, and carrier-associated fractions are adequately separated during analysis [7,9,13].

Phase-separated OA may occur as drug-rich domains, external crystals, or aggregates formed during preparation, drying, storage, or release testing. Such separation can reduce dose uniformity and cause incomplete or poorly reproducible release. It may also create differences between the nominal OA concentration and the fraction that is actually available to cells or tissues [1,5,8,9].

### 3.4. Low Bioavailability and Formulation-Dependent Exposure

Low and variable bioavailability is one of the main reasons OA has attracted formulation interest. For orally or systemically administered OA, poor dissolution, precipitation, limited permeability, metabolism, and vehicle-dependent absorption can reduce and destabilize systemic exposure [1,5,19,20]. However, formulation studies should distinguish apparent solubility or dispersion, biological exposure, systemic bioavailability, and therapeutic benefit.

Apparent solubility or dispersion describes the amount of OA maintained in a dissolved, colloidal, carrier-associated, or otherwise measurable state under defined experimental conditions. Biological exposure describes the concentration and duration of OA availability in a relevant compartment, including cells, skin layers, joint space, tumor tissue, or systemic circulation. Systemic bioavailability refers specifically to the rate and extent at which OA reaches systemic circulation. Therapeutic benefit requires that the achieved exposure produces a relevant and safe biological response.

These outcomes are related but not interchangeable. A formulation may increase apparent OA dispersion without improving absorption if OA precipitates after dilution, remains strongly retained within the carrier, is degraded, or fails to cross the relevant biological barrier. Conversely, a sustained-release system may produce lower short-term free-drug concentrations while improving exposure duration or tissue retention. Greater cellular activity also cannot be assumed to demonstrate improved in vivo efficacy unless uptake, tissue exposure, pharmacokinetics, and safety are evaluated [5,9,12,17].

For local delivery, systemic bioavailability may not be the primary objective. Topical gels, wound dressings, microneedles, and intra-articular depots should instead be evaluated according to route-specific exposure, such as OA deposition in skin layers, persistence at a wound surface, residence in the joint, or availability within target cells [12,14,15,16,18]. In these settings, the relevant question is whether the formulation reproducibly maintains OA at the intended site and produces a measurable biological advantage over simpler delivery approaches.

### 3.5. Permeability Limitations and Biological Barriers

OA delivery is also limited by biological barriers, including the gastrointestinal epithelium, stratum corneum, mucus, extracellular matrix, synovial environment, and cellular membranes. Although OA is lipophilic, lipophilicity alone does not ensure efficient absorption or tissue penetration. Poor aqueous solubility can limit the concentration gradient required for transport, while strong partitioning into carrier or tissue compartments can reduce the amount of freely available OA [1,5].

Polymeric systems can be designed to interact with these barriers in different ways. PEGylated carriers may reduce aggregation and modify protein interactions, hyaluronic-acid-based systems may exploit receptor-mediated uptake in CD44-expressing cells, and hydrogels or microneedles can improve local contact time or bypass the stratum corneum barrier. These mechanisms are route-specific rather than universal. Hydrogels and microneedles can support local tissue delivery by prolonging contact time or bypassing the stratum corneum barrier [7,8,12,13,16,18].

A polymeric OA nanoparticle intended for cancer-cell uptake therefore requires different design criteria from a hydrogel intended for wound treatment or a microneedle patch intended for intradermal delivery [7,8,9,12,16,18]. Carrier selection should begin with the biological barrier and the intended site of action, not simply with the aim of increasing solubility [5,6,13].

### 3.6. Vehicle-Dependent Biological Activity

A recurring difficulty in OA research is that biological activity depends strongly on the form in which the compound is delivered [5,17]. OA dissolved in an organic solvent, complexed with cyclodextrin, encapsulated in PLGA nanoparticles, incorporated into micelles, or conjugated to hyaluronic acid may produce different exposure patterns even when the nominal OA dose is similar [7,9,12,17].

This vehicle dependence complicates interpretation. A stronger response from an OA-loaded nanocarrier may reflect improved dispersion, reduced precipitation, enhanced cellular uptake, altered intracellular localization, slower release, higher tissue retention, or carrier-associated effects. It should not automatically be interpreted as increased intrinsic potency of OA [5,7,9,12].

Appropriate controls are therefore essential. Studies should compare blank carrier, free OA, solubilized OA, and carrier-loaded OA whenever possible, and the actual OA concentration available under assay conditions should be analytically verified [9,13,17]. Without these controls, it is difficult to determine whether the observed biological effect comes from OA itself, the carrier, the solubilizing environment, or the combined drug–carrier system [5,13,17].

### 3.7. Why Oleanolic Acid Is Suitable for Polymeric Nanocarrier Engineering

Oleanolic acid is a useful payload for polymeric nanocarrier research because it combines biological relevance with difficult formulation behavior [1,5]. Its poor solubility, hydrophobicity, crystallinity, limited bioavailability, barrier-dependent transport, and vehicle-dependent activity create a demanding test case for controlled delivery systems [5,7,9].

The formulation question is no longer whether OA can be incorporated into a carrier. This has already been shown using PEGylated PLA/PLGA nanoparticles, PLGA nanoparticles, hyaluronic-acid-based systems, polymeric micelles, gels, and PLGA fiber membranes [7,8,9,12,16,18]. The more important question is how polymer composition, carrier architecture, drug physical state, release environment, and biological model determine performance [5,6,7,8,9,12,13,16,18].

This shift is important for the whole field. Polymeric and polymer-assisted OA systems should not be judged only by loading efficiency or particle size. They should be judged by whether the carrier creates a reproducible, route-appropriate exposure profile that improves the interpretation and potential usefulness of OA in a defined biological setting [5,6,7,8,9,12,13].

The main formulation-relevant barriers of OA and their implications for polymeric or polymer-assisted carrier design are summarized in Table 1.

## 4. Current Delivery Strategies for Oleanolic Acid: Positioning Polymeric and Polymer-Assisted Systems

Several formulation strategies have been explored to improve the delivery of oleanolic acid, including solid dispersions, cyclodextrin complexes, phospholipid complexes, self-emulsifying systems, liposomes, lipid nanoparticles, polymeric nanoparticles, protein-based carriers, hydrogels, and local depot systems [1,5,7,8,12]. This broad formulation landscape reflects the central difficulty of OA delivery: the compound is biologically attractive, but its performance depends strongly on how it is dispersed, stabilized, released, and presented to cells or tissues [1,5].

Conventional solubilization approaches, such as solid dispersions and cyclodextrin complexes, are useful because they can improve OA wettability, reduce crystallinity, or increase apparent aqueous compatibility. Their main value is to show that OA activity is not independent of presentation format. However, these systems do not necessarily provide sustained release, tissue retention, or route-specific control over exposure [5,17,23].

Self-emulsifying and self-nanoemulsifying systems are particularly relevant for oral delivery [1,5,19,20]. By maintaining OA in solubilized or lipid-associated states, they may improve dispersion in gastrointestinal fluids and increase the fraction available for absorption [19,20]. Their performance, however, can depend on surfactant concentration, dilution behavior, lipid digestion, precipitation risk, and gastrointestinal variability [1,5,19,20]. These factors make cross-study comparison difficult and show why solubilization alone is not sufficient for translational development.

The available OA literature illustrates the distinction between improved formulation behavior and demonstrated biological benefit. Cyclodextrin complexation has been reported to improve OA cell bioavailability and migration-related biological activity compared with DMSO-delivered OA, but this finding does not by itself establish increased systemic bioavailability [17]. PEGylated PLA/PLGA nanoparticles and OA/ursolic-acid-loaded PLGA nanoparticles altered cytotoxic responses in cancer-cell models, indicating changes in cellular exposure, uptake, or release; however, these in vitro effects should not be interpreted as evidence of improved in vivo anticancer efficacy [7,8]. Stronger route-specific evidence has been reported for the hyaluronic-acid-based OA nanoprodrug in a psoriasis model, in which improved cellular uptake was accompanied by enhanced anti-psoriasis activity [12]. Topical liquid-crystalline nanoparticle gels and thermosensitive intra-articular OA systems have likewise demonstrated biological responses in application-relevant animal models [15,18]. Together, these examples show that the most convincing formulation studies connect physicochemical improvement with measured exposure at the target site and a relevant biological outcome.

Lipid-based nanocarriers, including liposomes, solid lipid nanoparticles, nanostructured lipid carriers, and liquid crystalline nanoparticles, are compatible with OA because of its lipophilic triterpenoid structure [18,21,22]. They can improve dispersion, protect OA from direct aqueous exposure, and support local or systemic delivery. At the same time, lipid-based systems may face problems such as drug leakage, lipid oxidation, polymorphic transitions, drug expulsion during storage, and incomplete control over release after dilution in biological media [1,5,18,21,22].

Polymeric nanocarriers and polymer-assisted delivery platforms occupy a distinct position because they can provide functions that are difficult to obtain through solubilization alone. Hydrophobic polymer matrices can accommodate OA and regulate diffusion or degradation-dependent release; PEGylated surfaces can modify colloidal stability and biological interactions; hyaluronic-acid-based systems can combine drug conjugation with disease-oriented cellular interaction; and hydrogels, fiber membranes, depots, or microneedles can control local residence and route-specific administration [7,8,9,12,13,16]. These advantages do not make polymeric systems universally preferable to cyclodextrins, liposomes, or self-emulsifying formulations. Their additional material and manufacturing complexity is justified only when sustained release, physical-state stabilization, tissue retention, barrier bypass, responsive release, or a measurable improvement in target-site exposure is required.

In this review, polymeric nanocarriers are defined as nanoscale systems in which the principal carrier architecture is formed by polymers, including PLGA or PLA nanoparticles, PEGylated polymeric nanoparticles, polymeric micelles, nanogels, and polymer–drug nanoprodrugs [7,8,9,12]. Polymer-assisted delivery platforms are defined more broadly and include systems in which a polymer provides surface stabilization, gelation, local retention, controlled release, mechanical administration, or barrier-bypass functions. This group includes polymeric fiber membranes, nanocarrier-loaded hydrogels, thermosensitive depots, polymer-modified lipid systems, and polymeric microneedle matrices [8,10,16,18,21,22].

This distinction is useful because not all OA delivery systems solve the same problem. A self-emulsifying system primarily improves dispersion; a liposome provides lipid-associated delivery; a PLGA nanoparticle offers matrix-based release; a hyaluronic-acid nanoprodrug adds biological interaction and responsive cleavage; and a thermosensitive gel supports local residence after administration [7,8,12,18,21,22]. These differences should guide how each formulation is evaluated.

**Figure 2:**
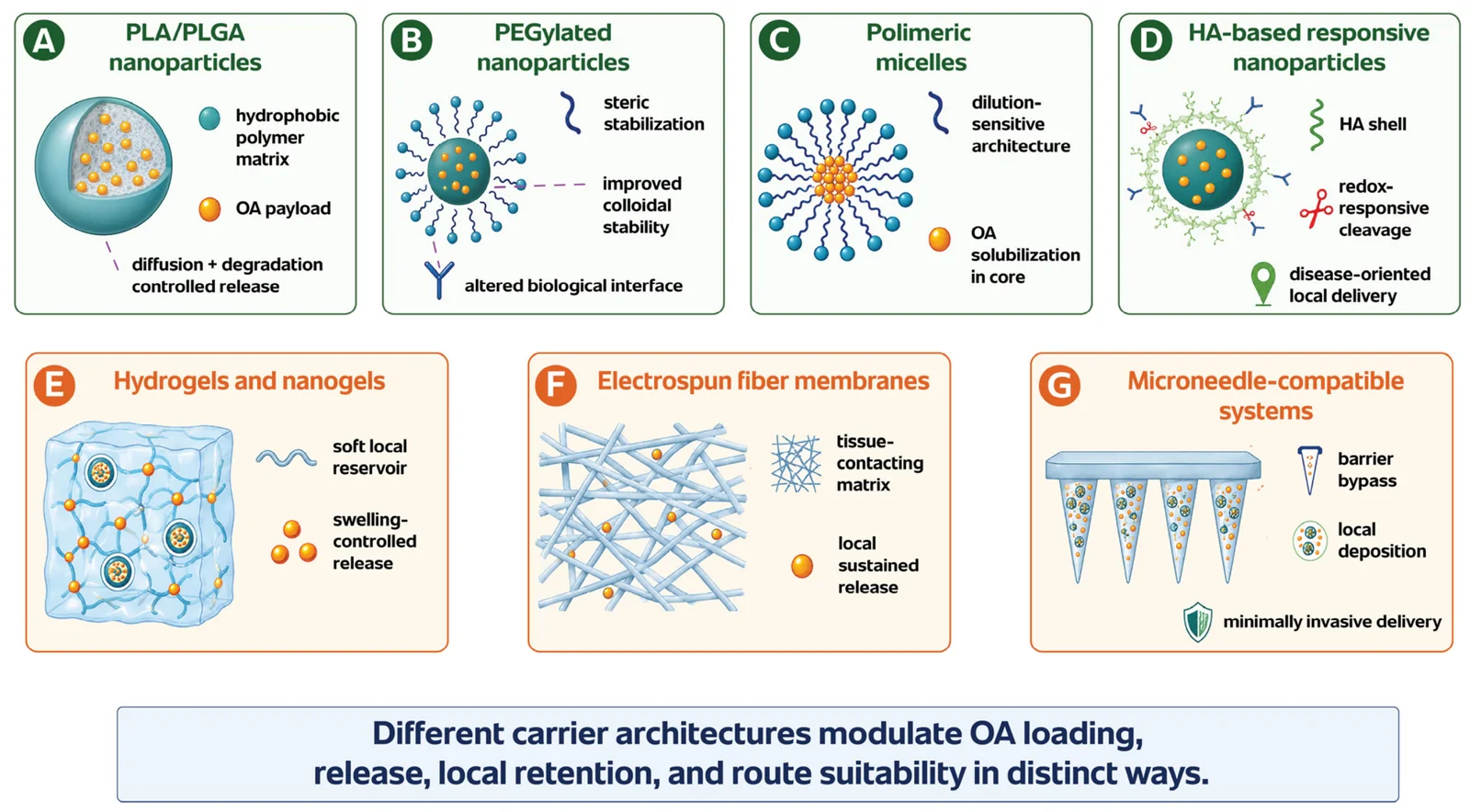
Architecture of major polymeric nanocarriers and polymer-assisted delivery platforms for OA delivery. (**A**) PLA/PLGA nanoparticles providing a hydrophobic polymer matrix and diffusion- or degradation-controlled release. (**B**) PEGylated nanoparticles providing steric stabilization and an altered biological interface. (**C**) Polymeric micelles solubilizing OA within hydrophobic cores. (**D**) Hyaluronic-acid-based responsive nanoparticles enabling disease-oriented local delivery and redox-responsive cleavage. (**E**) Hydrogels and nanogels acting as swelling-controlled local reservoirs. (**F**) Electrospun fiber membranes providing tissue-contacting matrices and sustained local release. (**G**) Microneedle-compatible systems enabling barrier bypass and local deposition. Orange circles represent OA or OA-loaded domains, whereas arrows and pathway symbols indicate release, transport, or deposition processes. Colors distinguish the principal carrier classes and delivery functions.

The current evidence supports a focused discussion of polymeric and polymer-assisted OA delivery. The field has moved beyond the simple question of whether OA can be formulated. Multiple carrier types have already demonstrated that it can [1,5,7,8,12]. The more important question is which polymeric design principles improve release control, route suitability, biological interpretation, reproducibility, and development potential [7,8,9,10,12,13,16]. The main polymeric and polymer-assisted carrier architectures discussed in this review are summarized in Figure 2.

This classification helps avoid treating all OA nanocarriers as equivalent. A self-emulsifying system mainly improves dispersion; a liposome may support membrane-associated delivery; a PLGA nanoparticle can provide matrix-based release; a hyaluronic-acid nanoprodrug can introduce receptor-mediated and redox-responsive behavior; and a thermosensitive nanogel can prolong local residence after administration. These systems differ not only in composition but also in how they control OA availability over time [7,8,12,18,21,22].

For translational purposes, polymeric and polymer-assisted carriers are especially relevant because they can be designed around unmet clinical needs rather than only around solubility improvement [7,8,9,10,12,13,16]. In cancer-oriented studies, OA-loaded PEGylated PLA/PLGA nanoparticles and OA/UA-loaded PLGA nanoparticles have been evaluated for cytotoxic activity in cancer cell models [7,9]. In inflammatory skin disease, hyaluronic-acid-based reduction-responsive OA nanoparticles have been developed for topical psoriasis therapy [12]. In local tissue delivery, OA-loaded PLGA fiber membranes and OA-loaded thermosensitive nanogels illustrate the potential of polymeric matrices and polymer-assisted depots for sustained exposure [8,18].

The current delivery landscape therefore supports a focused review on polymeric and polymer-assisted OA systems. The main question is no longer whether OA can be formulated; multiple carrier types have already demonstrated that it can [1,5,7,8,12]. The more important question is which polymeric design principles can improve controlled release, route-specific delivery, biological relevance, reproducibility, and translational feasibility [7,8,9,10,12,13,16].

Representative quantitative characteristics of OA-loaded polymeric nanocarriers and polymer-assisted delivery platforms are compared in Table 2. The reported systems differ substantially in composition, administration route, experimental model, release method, and biological endpoint; consequently, the values should be used to identify formulation-dependent trends rather than to establish a direct ranking of carrier performance.

The quantitative comparison reveals considerable heterogeneity among OA delivery studies. Reported particle sizes ranged from approximately 57 nm for polymeric micelles to about 250 nm for PEGylated PLA/PLGA nanoparticles, whereas polymeric fiber membranes had mean fiber diameters of approximately 0.5–0.6 µm [7,8,9,14]. Encapsulation efficiencies ranged from approximately 41% to more than 99%, but high encapsulation did not consistently predict faster release or stronger biological activity [7,9,14].

For example, the mPEG–PLGA nanoparticles described by Man et al. exhibited lower OA encapsulation efficiency than the corresponding mPEG–PLA systems but produced stronger cytotoxic effects in cancer cells, which the authors associated with differences in polymer degradation and cellular interaction [7]. Conversely, OA/UA-loaded PLGA nanoparticles released approximately 75% of their payload over 72 h but produced higher IC50 values than the corresponding free mixtures in Y-79 cells, illustrating that controlled release can reduce immediate cellular exposure rather than increase short-term cytotoxicity [9]. These findings show that carrier capacity and biological potency should not be interpreted as interchangeable measures of formulation performance.

The local systems provide a different pattern. Polymeric micelles increased both skin retention and permeation of OA relative to non-micellar formulations, while the HA-based nanoprodrug reduced the IC50 in keratinocytes and produced dose-dependent therapeutic effects in a psoriasis model [12,14]. The topical LCNP gel achieved substantial release and ex vivo permeation within 12 h, whereas PLGA fiber membranes released less than 30% of incorporated OA over 14 days [8,15]. These differences do not identify one universally superior platform; instead, they demonstrate that the appropriate release rate depends on whether the intended function is dermal deposition, rapid anti-inflammatory action, prolonged wound-surface exposure, or sustained intra-articular retention.

The available evidence also has important limitations. Several studies reported encapsulation efficiency but not complete mass balance, and some did not distinguish molecularly dispersed OA from amorphous, crystalline, surface-associated, or phase-separated drug. Release protocols varied in medium composition, sink conditions, membrane selection, and study duration. Biological endpoints ranged from short-term cell viability to skin permeation, cosmetic outcomes, psoriasis severity, and osteoarthritis-related behavior. Therefore, the current literature supports formulation-specific conclusions but does not yet permit a formal quantitative ranking or meta-analysis of OA polymeric delivery systems.

## 5. Polymeric Nanocarriers and Polymer-Assisted Delivery Platforms for Oleanolic Acid

### 5.1. Classification and Design Rationale

Polymeric OA delivery systems can be divided into two related but functionally distinct groups. The first comprises nanoscale carriers in which the principal architecture is formed by a polymer, including PLA/PLGA nanoparticles, PEGylated polymeric nanoparticles, polymeric micelles, nanogels, and polymer–drug nanoprodrugs. The second comprises polymer-assisted systems in which a polymer modifies the surface of another carrier, stabilizes a dispersed drug phase, forms a local matrix, or controls administration and release [7,8,9,12,13,14,15,18].

This distinction is important because these systems do not solve the same delivery problem. PLA and PLGA nanoparticles primarily provide a biodegradable hydrophobic matrix for OA incorporation and sustained release. PEGylated architectures add steric stabilization and alter the biological interface. Polymeric micelles provide dynamic hydrophobic domains for aqueous dispersion. Hyaluronic-acid-based nanoprodrugs combine carrier formation with polymer–drug conjugation and disease-oriented interaction. Gel-based and hybrid systems additionally regulate tissue contact, local retention, and route-specific administration [7,8,9,12,14,15,18].

For OA, polymeric design is justified when it provides a function beyond temporary solubilization. Relevant functions include control of drug physical state, prolonged release, protection against precipitation, surface modification, local retention, biological-barrier bypass, or integration with a tissue-facing dosage form [1,5,7,8,9,12,14,15,16,18]. Polymeric systems should not be assumed to be universally superior to cyclodextrin complexes, lipid carriers, or self-emulsifying formulations. Their increased complexity is justified only when it produces a measurable advantage in stability, exposure, release control, route suitability, or biological performance.

### 5.2. PLA and PLGA Nanoparticles

Biodegradable PLA and PLGA nanoparticles are among the most relevant polymeric carriers for OA because they can accommodate hydrophobic molecules within polymer matrices and release them through diffusion, matrix relaxation, hydrolytic degradation, and erosion [7,9,13,24]. Their molecular weight, lactide:glycolide ratio, end-group chemistry, hydrophobicity, and degradation behavior can influence nanoparticle formation, OA loading, release kinetics, and storage stability [6,13].

One of the most direct OA examples was reported by Man et al., who prepared OA-loaded mPEG–PLA and mPEG–PLGA nanoparticles by nanoprecipitation and evaluated their physicochemical properties, encapsulation efficiency, and cytotoxic activity in cancer-cell models [7]. The hydrophobic polyester region accommodated OA, whereas the PEG segment improved colloidal dispersion and modified the particle surface. The study demonstrated that biodegradable polymeric nanoparticles can change the presentation and cellular effects of OA compared with non-encapsulated drug [7].

PLGA nanoparticles have also been used for OA together with or in comparison with ursolic acid. Silva et al. evaluated OA/ursolic-acid-loaded PLGA nanoparticles in several cell lines and showed that encapsulation altered cell-specific activity. This comparative approach is relevant because OA and ursolic acid are structurally related pentacyclic triterpenoids with similar formulation limitations, but they do not necessarily exhibit identical biological responses.

The biological effects reported for these systems require cautious interpretation. Greater cytotoxicity after encapsulation may result from improved dispersion, reduced precipitation, altered uptake, intracellular delivery, or modified release rather than an increase in the intrinsic pharmacological potency of OA [1,5,7,9]. Polymeric nanoparticles should therefore be treated as integrated drug–carrier systems rather than as passive containers.

### 5.3. PEGylated and Surface-Modified Systems

PEGylation is a common strategy for improving colloidal stabilization and modifying interactions between nanocarriers and biological environments. Polyethylene glycol can reduce aggregation, increase surface hydration, and influence protein adsorption, cellular uptake, tissue penetration, and clearance [6,7,13]. For OA, PEGylation is useful because it combines a hydrated outer layer with a hydrophobic carrier domain capable of accommodating the drug.

OA-loaded mPEG–PLA and mPEG–PLGA nanoparticles illustrate this architecture directly [7]. Related polymer-assisted systems include PEGylated lipid carriers, in which the principal structure is lipidic but the PEG layer determines surface stabilization and biological interactions [22]. These systems are included as polymer-assisted platforms rather than purely polymeric nanoparticles.

PEGylation should not be described as an automatic improvement. PEG molecular weight, chain density, surface distribution, and carrier architecture can influence both stability and biological availability. A dense PEG corona may reduce aggregation but also decrease cellular interaction or slow drug transfer to the target compartment [6,7,13,22]. PEGylated OA carriers should therefore be evaluated using stability, release, uptake, tissue-exposure, and biological-response data rather than particle size and encapsulation efficiency alone.

### 5.4. Preparation Methods and Process Sensitivity

OA-loaded polymeric nanoparticles have most often been prepared by nanoprecipitation, solvent displacement, or emulsion-based methods [7,9]. These processes are compatible with hydrophobic compounds, but they are also sensitive to solvent selection, polymer and drug concentration, aqueous-phase composition, temperature, stabilizer type, mixing conditions, and solvent-removal procedure [7,9,13].

For OA, uncontrolled supersaturation can cause drug precipitation outside the polymer matrix, whereas rapid polymer precipitation can produce heterogeneous particles or reduce drug incorporation. Conversely, strong drug–polymer association may increase encapsulation but lead to excessively slow or incomplete release [1,5,9,13]. The final formulation therefore reflects both material selection and process history.

Nanoprecipitation is attractive because of its technical simplicity and potential scalability, but it should not be treated as a fully standardized method. Injection rate, solvent-to-water ratio, vessel geometry, mixing intensity, purification, and drying can alter particle nucleation, size distribution, residual solvent, OA loading, physical state, and burst release [7,9,13,24].

Preparation parameters should be reported in sufficient detail to allow reproduction. Polymer source and molecular weight, drug-to-polymer ratio, solvent composition, stabilizer concentration, mixing procedure, purification, drying, storage, and the number of independently prepared batches should be specified whenever possible. An OA-loaded carrier should be evaluated as the product of a defined manufacturing process rather than only as a final particle suspension.

**Figure 3:**
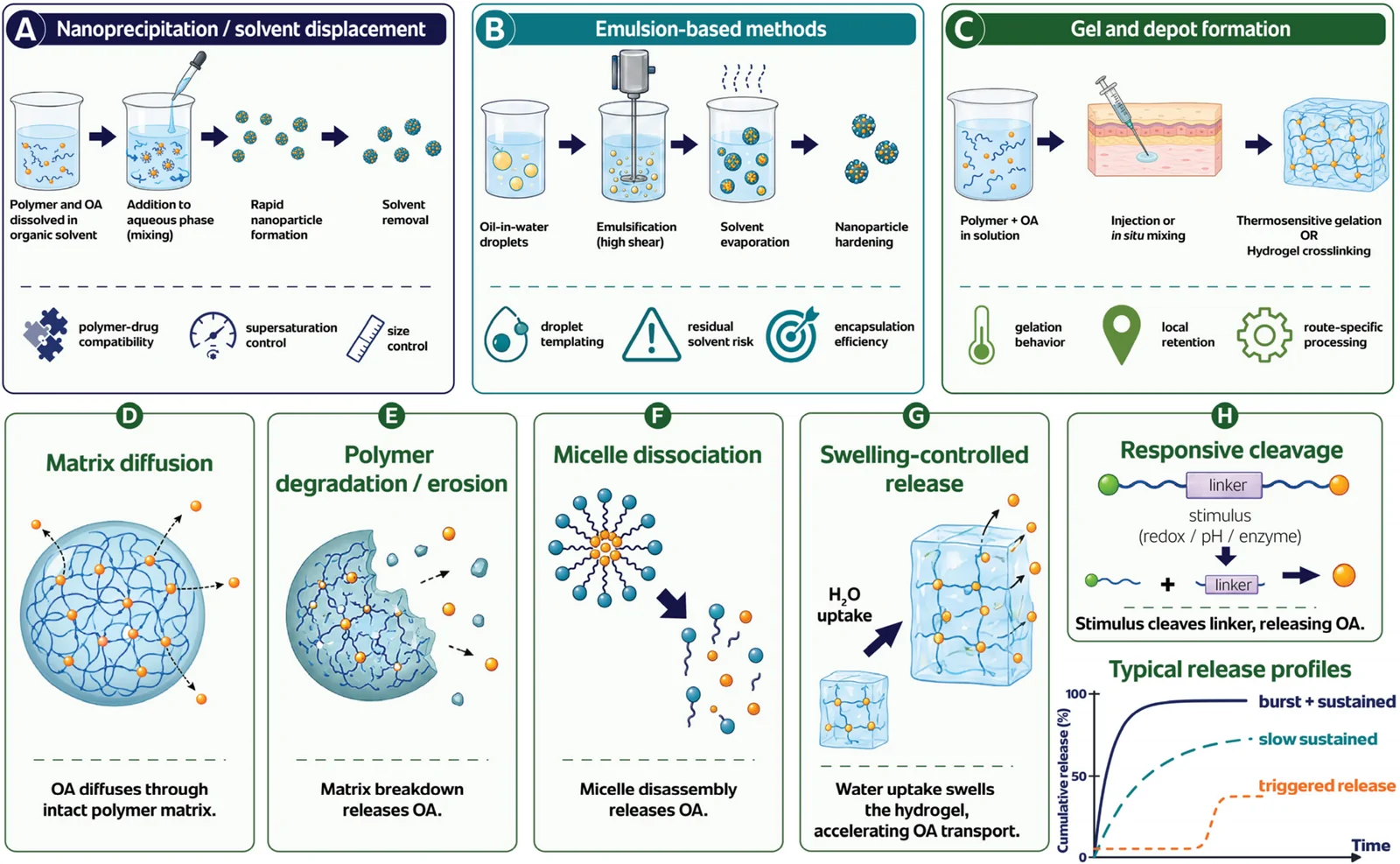
Preparation methods and release mechanisms in OA-loaded polymeric nanocarriers and polymer-assisted delivery platforms. (**A**) Nanoprecipitation or solvent displacement. (**B**) Emulsion-based preparation methods. (**C**) Gel and depot formation. (**D**) Matrix-diffusion-controlled release. (**E**) Release associated with polymer degradation or erosion. (**F**) Release through micelle dissociation. (**G**) Swelling-controlled release. (**H**) Stimuli-responsive cleavage and representative burst, sustained, and triggered release profiles. Arrows indicate the sequence of preparation steps or the direction of OA release, orange circles represent OA molecules, and colored carrier elements represent polymeric matrices or nanostructures.

The preparation routes and release mechanisms most relevant to OA-loaded polymeric systems are summarized in Figure 3.

### 5.5. Drug Loading, Physical State, and Release Behavior

Drug loading and encapsulation efficiency are central parameters, but they do not fully describe the state of OA within a carrier [7,9,13]. Apparent encapsulation may include OA molecularly dispersed in the polymer matrix, drug associated with amorphous regions, surface-bound OA, or small crystalline aggregates that remain after purification [1,5,7,9]. These fractions can differ substantially in release rate, storage stability, and biological availability.

A formulation with high encapsulation efficiency may still perform poorly if OA crystallizes, remains trapped within a hydrophobic matrix, or undergoes rapid surface-associated burst release. Two formulations with similar OA content may therefore produce different release and biological profiles. Quantitative drug assays should be accompanied by solid-state and morphological characterization capable of detecting crystallinity, phase separation, surface-associated drug, and drug–polymer interactions [1,5,7,8,9,13].

Release from PLA and PLGA nanoparticles can involve diffusion, water penetration, matrix relaxation, hydrolytic degradation, erosion, and redistribution of OA between the polymer phase and the release medium [6,13]. Because OA is highly lipophilic, the observed profile also depends on protein binding, surfactant concentration, lipid partitioning, sink conditions, and the physical state of the drug [1,5,7].

Release methods should therefore reflect the intended route. Simple aqueous buffers may underestimate OA release, whereas media containing excessive surfactant or organic cosolvent may overestimate biologically relevant availability. Skin-oriented, intra-articular, oral, and systemic carriers require different release conditions and different definitions of successful exposure [5,7,8,9,13,15,18].

### 5.6. Biological Evaluation of Polymeric OA Nanoparticles

Biological evaluation of OA-loaded polymeric nanoparticles has frequently focused on anticancer activity, especially for PLA/PLGA-based systems [7,9]. These studies show that encapsulation can alter cellular response, but two-dimensional cytotoxicity assays provide only early evidence of formulation performance and should not be interpreted as proof of clinical anticancer potential.

Appropriate controls are essential because OA activity is strongly influenced by the formulation vehicle. Studies should compare blank carrier, free OA, appropriately solubilized OA, and OA-loaded carrier under conditions where both OA and carrier concentrations are known [1,5,7,9]. Whenever possible, the released or cell-associated OA fraction should also be measured analytically.

The biological model should correspond to the proposed application. Cancer-oriented systems require uptake, penetration, three-dimensional models, pharmacokinetics, biodistribution, and safety evaluation as development progresses. Skin-oriented systems require keratinocyte, immune-cell, reconstructed-skin, deposition, and irritation studies. Joint-oriented formulations require cartilage-, synovium-, and osteoarthritis-relevant models [7,9,12,18].

Increased apparent solubility or cellular activity should not be treated as equivalent to improved therapeutic exposure. The value of a polymeric OA formulation depends on whether the carrier creates a reproducible link between dose, release, target-compartment exposure, and biological response.

### 5.7. Comparative Design and Translational Implications

The suitability of an OA carrier depends on the delivery problem being addressed. Conventional PLA/PLGA nanoparticles offer a relatively established biodegradable matrix and are most relevant when sustained release and nanoscale dispersion are required. PEGylated nanoparticles provide additional colloidal stabilization but require control of surface architecture and biological interaction. Polymeric micelles are useful for dermal dispersion but may be sensitive to dilution. Hyaluronic-acid nanoprodrugs introduce disease-oriented and responsive functions but add manufacturing and analytical complexity. Gel-based and hybrid systems are especially relevant when local residence and route-specific administration are the principal objectives.

The strongest current rationale for polymer-assisted OA delivery lies in local and tissue-targeted applications, including inflammatory skin disease, psoriasis, dermal delivery, wound-related applications, and osteoarthritis [12,14,15,18]. In these settings, sustained tissue exposure may be more relevant than increased systemic concentration. Cancer-oriented polymeric nanoparticles remain important scientifically, but their development requires stronger evidence on tumor penetration, pharmacokinetics, biodistribution, safety, and comparison with existing treatments [7,9].

No single carrier type should be considered optimal for OA. Formulation selection should be guided by the intended route, required release duration, OA physical state, target-compartment exposure, manufacturing complexity, and clinically relevant comparators. The most useful system is not necessarily the most complex one, but the one in which every polymeric component has a defined and measurable delivery function.

The functional roles, advantages, limitations, and representative applications of polymeric materials and polymer-assisted platforms used in OA delivery are summarized in Table 3.

## 6. Stimuli-Responsive and Soft Polymeric Systems for Oleanolic Acid Delivery

### 6.1. Rationale for Soft Polymeric Systems

Soft polymeric systems include polymeric micelles, nanogels, hydrogels, cyclodextrin-containing networks, thermosensitive gels, and stimuli-responsive polymer conjugates. They are relevant to OA delivery because they can combine drug dispersion with mechanisms that regulate release, tissue contact, and local residence [1,5,7,13,18,26,28]. Unlike rigid nanoparticles, these systems often contain hydrated, deformable, or supramolecular domains that respond to dilution, pH, redox conditions, enzymes, temperature, swelling, or the local tissue environment [12,13,15,18,26,28].

For OA, this flexibility is useful because the compound is poorly soluble, lipophilic, and strongly dependent on its formulation environment [1,5]. A rigid polymeric particle may encapsulate OA efficiently but release it too slowly. A simple solubilizing system may improve dispersion but fail to maintain local exposure. Soft polymeric systems occupy the intermediate space between these two extremes: they can keep OA in a dispersed state while allowing release to be influenced by polymer hydration, gel swelling, micelle dissociation, complex dissociation, degradation, or responsive bond cleavage [12,13,15,18,26,28].

This section therefore does not treat soft systems as another list of carrier types. Instead, it focuses on how their dynamic structure can be used to control OA availability. This distinction is important for local and barrier-associated applications, including inflammatory skin disease, psoriasis, wound healing, osteoarthritis, and dermal delivery, where sustained tissue exposure may be more useful than high systemic concentrations [12,14,15,16,18].

### 6.2. Polymeric Micelles as Solubilizing and Dermal Delivery Systems

Polymeric micelles are formed by amphiphilic polymers that self-assemble into structures with hydrophobic cores and hydrophilic shells. This architecture is compatible with OA because the hydrophobic core can accommodate the triterpenoid, while the shell improves dispersion in aqueous or semiaqueous media [7,14,31].

An et al. developed OA polymeric micelles and evaluated particle size, morphology, encapsulation efficiency, skin permeation, three-month physical stability, and anti-wrinkle performance in a cosmetic formulation [14]. Although the application was cosmetic, the study is relevant to polymeric drug delivery because it shows that OA can be converted into a stable nanoscale dispersed form suitable for skin application [14].

The main limitation of micellar systems is that solubilization does not necessarily equal delivery. Micelles can dissociate after dilution, exchange unimers with the surrounding medium, bind proteins, release OA prematurely, or retain the drug too strongly depending on polymer composition and hydrophobic core structure. For OA, the key question is whether micellar encapsulation increases the fraction that reaches relevant skin or tissue layers. Future micellar OA systems should therefore be evaluated using dilution stability, release kinetics, skin deposition, and biological assays matched to dermal or topical use [14,31].

### 6.3. Nanogels as Hydrated Polymeric Reservoirs

Nanogels are nanoscale crosslinked polymer networks that can swell in water or biological fluids while maintaining structural integrity. Their hydrated structure makes them attractive for controlled release because drug diffusion can be influenced by mesh size, crosslinking density, polymer composition, swelling behavior, and environmental responsiveness [26,28].

For OA, nanogels are conceptually attractive but technically demanding. Many nanogel networks are hydrophilic, whereas OA is highly lipophilic [1,5,26,28]. Useful OA loading may therefore require hydrophobic domains, inclusion complexes, lipidic substructures, polymer–drug conjugation, or hybrid nanocarrier-in-gel designs [12,15,18]. In other words, the nanogel itself may not be sufficient unless its structure contains a domain capable of accommodating OA.

The OA cubic liquid crystal nanoparticle-based thermosensitive gel developed for knee osteoarthritis illustrates this hybrid logic. The internal nanostructure accommodates OA, whereas the thermosensitive gel provides an injectable local depot that can prolong residence after intra-articular administration [18]. This design is relevant because it links nanoscale drug accommodation with macroscopic gel behavior.

Nanogels and soft gels may be especially useful in local inflammatory conditions, including osteoarthritis, inflamed skin, and wound-associated environments [12,15,18]. Their performance, however, cannot be predicted from drug loading alone. Rheology, swelling, degradation, injectability, gelation temperature, mechanical behavior, release kinetics, and local tolerability may all determine whether the system is useful for a given route [15,18,26,28].

### 6.4. Cyclodextrin-Containing and Cyclodextrin-Polymer Systems

Cyclodextrins are cyclic oligosaccharides that can form inclusion complexes with hydrophobic molecules and improve the apparent solubility or handling of poorly water-soluble compounds [29,30,34]. For OA, hydroxypropyl-cyclodextrin complexation improved stability, cell compatibility, and biological activity in migration-related assays compared with OA delivered in DMSO [17].

Simple cyclodextrin complexation is not always classified as polymeric nanocarrier delivery in the strict sense [17,29]. However, cyclodextrin-containing polymers, cyclodextrin-modified nanomaterials, cyclodextrin-based nanoparticles, and cyclodextrin nanosponges extend this inclusion-complex principle into polymeric delivery systems. These platforms can combine host–guest complexation with network formation, nanoparticle behavior, local retention, or controlled release [29,30,34].

For OA, cyclodextrin systems have two potential roles. First, they can improve aqueous compatibility and help standardize biological exposure in assays where DMSO-delivered OA may not be an ideal comparator. Second, when incorporated into polymeric networks or nanosponge-like structures, they may support more sustained release or local retention [17,29,30,34,35]. This could be useful for topical, wound-related, or microneedle-compatible formulations in which OA must remain dispersed within a hydrophilic polymeric environment [15,16,17,29].

The main caution is that inclusion complexation does not automatically predict tissue delivery. Complex stability, dissociation rate, competing biological molecules, polymer network structure, and route of administration can all affect OA release [17,29,30,34,35]. Cyclodextrin-polymer OA systems should therefore be compared with reference carriers such as PLGA nanoparticles, micelles, and hydrogels under matched biological conditions [7,14,17,29].

### 6.5. Redox-Responsive Hyaluronic-Acid Systems

Redox-responsive carriers are designed to release drug in response to differences between extracellular and intracellular redox environments. Disulfide linkages are commonly used for this purpose because they can remain relatively stable outside cells but undergo cleavage in more reductive intracellular compartments [12,26]. This mechanism is directly relevant to hyaluronic-acid-based OA nanoprodrugs developed for topical psoriasis therapy [12].

Han et al. reported disulfide-bonded OA–hyaluronic acid nanoprodrugs with additional OA encapsulation for psoriasis treatment [12]. The formulation was designed to address poor OA solubility, limited cutaneous permeation, and rapid clearance after topical application. The authors reported improved uptake through CD44 receptor-mediated endocytosis in keratinocytes and enhanced anti-psoriasis effects compared with free OA [12].

This system is one of the strongest OA examples because it combines several design elements in one platform: a hydrophilic polymeric framework, receptor-associated cellular interaction, redox-responsive cleavage, nanoscale assembly, and topical disease-oriented delivery [12]. The polymer is not only a stabilizer; it participates in both carrier architecture and biological interaction.

The main translational questions concern reproducibility and safety. The substitution degree of the OA–hyaluronic acid conjugate, disulfide stability, degradation products, batch consistency, skin irritation, repeated-dose safety, and comparison with standard psoriasis therapies all need to be clarified before such a platform can move beyond proof of concept [12,26].

### 6.6. Thermosensitive, pH-Responsive, and Enzyme-Responsive Systems

Stimuli-responsive polymeric systems can be designed to change swelling, degradation, drug release, or network structure in response to temperature, pH, enzymatic activity, or other local microenvironmental cues [12,15,18,26]. For OA, these mechanisms are attractive because they may help couple drug release to the biological compartment in which local exposure is needed. However, the strength of evidence differs between stimulus types. Thermosensitive OA systems have direct experimental support, whereas pH- and enzyme-responsive approaches remain more conceptual or extrapolated from broader polymeric drug-delivery literature unless OA-specific examples are available.

Thermosensitive polymeric systems are currently the most developed responsive local-depot approach for OA. They can be administered as liquids and form gels at physiological or tissue-relevant temperature, which is useful for topical or intra-articular applications where residence time is a major limitation [15,18,28]. The OA cubic liquid crystal nanoparticle-based thermosensitive gel developed for knee osteoarthritis illustrates this logic: the nanostructured carrier accommodates OA, while the temperature-responsive gel matrix supports local retention and sustained exposure after administration [18].

pH- and enzyme-responsive systems may also be relevant for OA, particularly in inflamed skin, wounds, tumors, or intracellular compartments where local pH, enzyme activity, or redox state may differ from healthy tissue [12,28]. In such systems, the stimulus should not be added as a decorative feature. It should have a clear mechanistic purpose, such as accelerating release in an inflamed microenvironment, promoting carrier degradation after tissue deposition, or reducing premature release before the formulation reaches the target site.

For all responsive OA systems, performance depends on whether the trigger is strong, reproducible, and relevant to the intended route. Gelation temperature, pH sensitivity, enzymatic degradation rate, polymer concentration, injectability, dilution behavior, mechanical strength, release kinetics, and tissue tolerability all need to be matched to the biological setting [15,18,28]. A responsive system is useful only if the stimulus improves local exposure, safety, or dosing practicality compared with a simpler formulation.

### 6.7. Integration with Microneedles, Hydrogels, and Wound Dressings

Soft polymeric OA systems are especially relevant when they are combined with dosage forms that solve a route-specific barrier. Microneedles can bypass the stratum corneum, hydrogels can maintain moist local contact, fiber membranes can provide tissue-facing matrices, and thermosensitive gels can improve residence after injection or topical placement [15,16,18,32,33].

This integration is important because OA delivery often requires more than one level of control. A nanocarrier may improve OA dispersion, but the dosage form determines where the carrier is placed, how long it remains there, and how the tissue environment affects release [15,16,18]. For example, nanocarrier-loaded microneedles can deposit OA-containing systems into the skin, after which release is governed by microneedle dissolution, carrier stability, tissue fluid interaction, and OA partitioning [16,32,33].

Similarly, hydrogels and wound dressings can combine local retention with controlled release. The polymeric matrix may regulate moisture balance, tissue contact, mechanical compliance, and residence time, while OA-loaded micelles, nanoparticles, or inclusion complexes improve drug dispersion within the matrix [8,15,18]. These combined systems are more complex than simple topical formulations, but they may be better matched to inflammatory skin disease, wounds, and local tissue injury.

Evaluation should reflect this multilevel design. For topical and transdermal OA systems, release data alone are not enough. Skin deposition, tissue distribution, irritation, barrier recovery, inflammatory biomarkers, mechanical performance, and comparison with current therapies are needed to determine whether improved solubilization translates into useful local delivery [12,14,15,16,17].

### 6.8. Advantages and Limitations of Stimuli-Responsive OA Systems

Stimuli-responsive and soft polymeric systems offer several advantages for OA delivery. They can improve dispersion, support controlled release, prolong local residence, enable disease-oriented interactions, and integrate with topical, intra-articular, or microneedle-based administration [12,14,15,18,26,28]. These properties are most relevant for applications where local tissue exposure matters more than systemic concentration.

Their main weakness is complexity. Performance may depend on polymer chemistry, swelling, degradation, micelle stability, inclusion complex dissociation, gelation behavior, responsive bond cleavage, tissue interaction, and environmental conditions [12,15,18,26]. This complexity increases the need for detailed characterization and may complicate manufacturing, storage, scale-up, and regulatory evaluation [26,28].

A second limitation is the difficulty of separating OA effects from carrier effects. Hyaluronic acid, cyclodextrins, gels, micellar polymers, and responsive linkers may influence tissue interaction or cellular response independently of OA [12,14,17,29]. Blank carrier controls, non-responsive carrier controls, free OA controls, solubilized OA controls, and analytically verified exposure conditions are therefore essential [26].

Finally, many responsive systems remain at proof-of-concept stage. For OA, a soft polymeric system becomes convincing only when it demonstrates reproducible preparation, stable storage, route-specific release, relevant tissue deposition, acceptable safety, and a clear advantage over simpler formulations or existing therapeutic options [12,15,18,26].

### 6.9. Mechanistic Implications

Soft and stimuli-responsive polymeric systems are valuable for OA delivery because they address more than poor solubility. They can regulate how OA is dispersed, retained, released, and presented to tissue [12,14,15,16,17,18,28]. Polymeric micelles support skin-compatible dispersion, nanogels and hydrogels can act as local reservoirs, cyclodextrin-polymer systems can combine inclusion complexation with controlled release, and hyaluronic-acid nanoprodrugs can introduce receptor-associated and redox-responsive behavior [12,14,17,29,30,34,35].

The most realistic applications are likely to be local or topical, including psoriasis, inflammatory skin disease, wound healing, dermal delivery, and osteoarthritis [12,14,15,16,18]. In these settings, the purpose is not to maximize systemic bioavailability, but to maintain OA exposure at the tissue site for a useful period. The best soft polymeric systems will therefore be those that connect carrier mechanism with route-specific performance, rather than those that add responsiveness without a clear therapeutic reason.

## 7. Hydrogels, Polymeric Wound Dressings, and Local Depot Systems

### 7.1. Why Local Polymeric Systems Are Important for Oleanolic Acid

Local polymeric delivery systems are particularly relevant for OA because several of its plausible biomedical applications involve tissue-level inflammation, skin pathology, wound repair, osteoarthritis, or localized oxidative stress rather than immediate systemic therapy [1,5,8,12,15,18]. In these settings, the formulation objective is not necessarily to maximize plasma concentration, but to maintain OA exposure at the target site for a useful period while limiting unnecessary systemic distribution [5,15,18].

This route-specific rationale follows directly from the formulation properties of OA. A system designed to improve gastrointestinal dispersion may not provide adequate wound-surface retention, whereas a nanoparticle optimized for cancer-cell uptake may not be appropriate for intra-articular or dermal administration [5,8,15,18]. Local polymeric matrices allow residence time, hydration, tissue contact, mechanical behavior, and release conditions to be adapted to the intended biological environment.

OA may first be incorporated into nanoparticles, micelles, inclusion complexes, or lipidic nanostructures and subsequently embedded within a tissue-facing polymeric matrix. In this multilevel design, the nanoscale carrier improves OA dispersion and physical-state control, whereas the hydrogel, fiber membrane, wound dressing, or thermosensitive depot determines administration-site retention, tissue contact, and the local release environment [8,12,14,15,18].

### 7.2. Hydrogels as Soft Matrices for OA Delivery

Hydrogels are useful biomaterials for local drug delivery because they contain high water content, can conform to tissue surfaces, may support moist wound environments, and can be engineered to release drugs over time. Their mechanical softness and tunable chemistry make them suitable for topical formulations, wound dressings, injectable depots, intra-articular systems, and microneedle-compatible platforms [12,35,36,37].

For OA, direct incorporation into a purely hydrophilic hydrogel may be inefficient because the compound is lipophilic and poorly soluble in aqueous media [1,5]. Hydrogel-based OA systems therefore often require an intermediate carrier, such as polymeric nanoparticles, liquid crystalline nanoparticles, polymeric micelles, cyclodextrin complexes, or nanogels, before incorporation into the hydrogel matrix [8,12,14,15,18]. This nanocarrier-in-gel approach is more rational than simply dispersing crystalline OA in a hydrated polymer network.

Hydrogels can regulate release through polymer concentration, crosslinking density, swelling, erosion, mesh size, temperature sensitivity, degradation, and interaction between the embedded carrier and the polymer network. For OA, these variables determine whether the compound is released too rapidly, retained too strongly, or made available at a sustained local concentration. Hydrogel-based OA systems should therefore be characterized not only by drug release, but also by rheology, swelling, mechanical behavior, carrier distribution, and stability during storage and use [8,14,15,18,35].

Hydrogel-based local delivery is most convincing when repeated administration is undesirable or when local residence is central to the therapeutic concept. This applies to chronic wounds, inflamed skin, intra-articular delivery, and post-injury tissue repair, where the desired effect may depend on prolonged anti-inflammatory, antioxidant, or cytoprotective exposure rather than short-term systemic absorption [12,14,18].

### 7.3. Topical OA Gels and Polymer-Assisted Local Retention

Topical OA gel systems illustrate how a polymer-assisted matrix can convert a nanocarrier into a more practical dosage form. OA-loaded liquid crystalline nanoparticle gels are relevant because the internal nanostructure can accommodate the lipophilic drug, while the gel phase improves topical application, tissue contact, and local residence. In such systems, the gel is not merely a thickening agent. It contributes to administration, retention, and release.

Shi et al. developed an OA-loaded cubic liquid crystalline nanoparticle-based topical gel and evaluated rheological behavior, release kinetics, ex vivo permeation, and anti-inflammatory performance [15]. The topical orientation is important because skin inflammation is a clinically plausible area for OA delivery. Local exposure may be more relevant than systemic bioavailability, and a gel-based format can reduce the need for systemic administration [1,5,15].

The main development questions for topical OA gels are practical rather than only nanoscale. A useful topical system should spread appropriately, remain at the application site, avoid irritation, maintain drug stability, and release OA under skin-relevant conditions [15,36,37]. Evaluation should therefore include rheology, skin deposition, permeation, irritation, barrier compatibility, microbial considerations where relevant, and comparison with established topical treatments [12,15,35].

### 7.4. Thermosensitive OA Nanogels for Intra-Articular Delivery

Thermosensitive OA nanogels represent one of the most disease-oriented examples of polymer-assisted local delivery. The OA cubic liquid crystal nanoparticle-based thermosensitive gel reported by Shi, Jia, Tang, and Li was designed for intra-articular administration in a rat knee osteoarthritis model [18]. The system combined OA-loaded nanoparticles with a Poloxamer thermosensitive gel base, creating a formulation intended to improve local residence after injection [18].

This approach is relevant because osteoarthritis is a localized degenerative and inflammatory joint disease. For such an indication, sustained intra-articular exposure may be more meaningful than systemic OA administration [18]. OA has anti-inflammatory and cartilage-protective potential, but its poor solubility and limited direct applicability make local depot design attractive [5,18].

The thermosensitive behavior is central to the formulation. It determines whether the system can be administered as a fluid and then form or maintain a depot-like structure under physiological conditions [18]. For intra-articular use, injectability, gelation temperature, viscosity, retention time, release kinetics, sterility, endotoxin control, and joint compatibility are critical quality attributes. These parameters should be considered together with nanoparticle size, OA loading, and drug physical stat [18,35,36].

This example strengthens the clinical orientation of the review because it frames OA delivery around an anatomical and therapeutic problem: local treatment of osteoarthritis. However, translation would require further work on long-term joint safety, repeated dosing, cartilage compatibility, synovial irritation, mechanical joint conditions, comparison with clinically used intra-articular therapies, and manufacturing reproducibility [18,35,36].

### 7.5. PLGA Fiber Membranes as Polymeric Local Delivery Platforms

OA-loaded PLGA fiber membranes represent a different local delivery strategy. Instead of placing OA in a spherical nanoparticle or soft gel alone, the drug is incorporated into a nonwoven biodegradable polymeric fiber matrix. Ahmed et al. developed OA-loaded poly(lactic-co-glycolic acid) fiber membranes and evaluated processing–structure–property relationships relevant to biomedical delivery [8].

This platform is important because polymeric fiber membranes provide a macroscopic tissue-contacting structure. Compared with nanoparticles alone, fiber membranes offer mechanical integrity, surface area, porosity, and a format compatible with wound dressings, local patches, transdermal platforms, or tissue-facing reservoirs [8,35,36,37]. These features are especially relevant when the formulation must remain at the application site and interact with tissue over time.

For OA, PLGA fiber membranes can help avoid direct application of crystalline drug by distributing the compound within a biodegradable polymeric matrix. Fiber processing, fiber diameter, morphology, drug loading, thermal behavior, drug distribution, crystallinity, and degradation can all influence how OA is presented to the local environment [8]. This makes the platform relevant to the broader process–structure–function logic of the review.

The translational value of fiber membranes will depend on more than drug release. Mechanical flexibility, adhesion, tensile strength, porosity, moisture behavior, sterilization, residual solvent, drug stability, degradation rate, and tissue compatibility must be considered before clinical usefulness can be claimed [8,35,36,37]. For wound applications, release testing should also consider wound-like media rather than relying only on simple buffer systems.

### 7.6. Polymeric Wound Dressings and OA Wound-Healing Relevance

Modern wound dressings are not passive covers. Polymeric dressings can regulate moisture, absorb exudate, protect tissue mechanically, support cell attachment, and deliver therapeutic agents locally [35,36,37]. Hydrogels, electrospun fibers, nanofiber membranes, nanohybrid dressings, and nanoparticle-loaded matrices are therefore relevant platforms for wound-oriented OA delivery [7,35,36,37].

OA is relevant to wound-directed formulations because it has been associated with anti-inflammatory activity, oxidative-stress modulation, and cell migration-related responses [1,5,14,38,39]. Earlier in vivo work also reported wound-healing activity for OA from natural sources, supporting the biological rationale for local wound applications [38,39]. At the same time, wound healing is stage-dependent and involves inflammation, migration, proliferation, extracellular matrix deposition, angiogenesis, and remodeling. OA wound systems should therefore be interpreted carefully and tested in models that reflect the biology of tissue repair [35,36,37,38].

A polymeric wound dressing can support OA delivery by combining local retention with a favorable wound microenvironment. Direct OA loading may be possible in some matrices, but a more robust design may involve OA-loaded nanoparticles, micelles, cyclodextrin complexes, nanogels, or lipidic nanostructures incorporated into the dressing [8,12,14,15]. In this arrangement, the carrier manages OA dispersion and release, while the dressing controls hydration, residence, exudate interaction, tissue contact, and mechanical protection.

Wound-oriented OA systems should include biologically meaningful endpoints in addition to drug release. Relevant readouts include fibroblast viability, keratinocyte migration, inflammatory cytokines, oxidative stress markers, collagen deposition, wound closure, infection risk, exudate interaction, and tissue compatibility [35,36,37,38]. Without these endpoints, it is difficult to determine whether OA release translates into improved wound repair.

### 7.7. Local Depots for Inflammatory Skin Diseases

Inflammatory skin disease is another plausible direction for local OA polymeric systems. OA has reported anti-inflammatory activity, while topical and intradermal delivery can provide high local exposure with potentially lower systemic burden [1,5,12]. Polymeric micelles, hyaluronic-acid-based nanoparticles, hydrogels, and microneedle-compatible matrices are particularly relevant because they address both OA solubility and skin-barrier limitations [12,14,16].

The hyaluronic-acid-based reduction-responsive OA nanoparticle system for psoriasis is a strong example of disease-oriented local polymeric design. It combines polymeric architecture, receptor-associated cellular interaction, redox-responsive release, and topical application in an inflammatory skin disease model [12]. This type of design is more convincing than a general solubility-enhancing formulation because it is linked to a specific disease context

Local OA systems for inflammatory skin disease should be evaluated in models that reflect barrier function, keratinocyte response, immune signaling, skin deposition, and irritation potential. Simple cytotoxicity assays are not sufficient. A topical anti-inflammatory OA formulation must provide local activity without damaging the barrier, provoking irritation, or relying on unrealistic exposure conditions [12,14,16].

Hydrogels and microneedles may strengthen this direction further. Hydrogels can improve residence and hydration, whereas microneedles can bypass the stratum corneum and deposit OA-loaded systems into viable epidermis or dermis [16,33,40]. For OA, this combination is attractive because poor solubility and limited passive penetration are both major barriers [1,5,16].

### 7.8. Local Depots for Osteoarthritis and Musculoskeletal Inflammation

Osteoarthritis is a particularly relevant target for polymer-assisted OA depots because it combines inflammation, cartilage degradation, oxidative stress, and chronic pain within an accessible anatomical compartment [18]. Intra-articular delivery can theoretically provide high local exposure while reducing systemic distribution, but rapid clearance from the joint remains a major challenge for small molecules and simple formulations [18,35].

The OA thermosensitive nanogel study directly addresses this problem by placing OA-loaded nanoparticles in a Poloxamer gel base for local knee delivery in a papain-induced osteoarthritis model. The study is therefore more than another solubility-enhancement example. It tests a local depot concept in a disease-relevant anatomical setting [18].

For musculoskeletal applications, the critical formulation question is not only how much OA is loaded, but how long the system remains in the joint and how release relates to biological response [18]. An ideal local depot should be injectable, minimally irritating, mechanically compatible with the joint, stable enough to prolong exposure, and degradable or removable without causing chronic inflammation [18,35].

Future OA osteoarthritis formulations should be compared with clinically relevant controls such as nonsteroidal anti-inflammatory drugs, corticosteroids, hyaluronic acid injections, or other intra-articular therapies where appropriate. They should also evaluate cartilage integrity, synovial inflammation, pain-related behavior, local toxicity, and repeated-dose safety. These endpoints are needed to determine whether polymer-assisted OA depots offer value beyond formulation novelty [18,35].

### 7.9. Design Criteria for OA Local Polymeric Systems

Local polymeric OA systems require design criteria that differ from oral or systemic nanocarriers [6,7,8,9]. For topical and wound systems, important parameters include tissue adhesion, hydration, breathability, exudate handling, irritation potential, microbial risk, drug release, skin or tissue deposition, and compatibility with damaged or inflamed tissue [8,12,35,36,37,38]. For intra-articular systems, injectability, gelation, residence time, sterility, endotoxin burden, mechanical compatibility, and local toxicity become more important [18,35].

Drug loading and release should be interpreted in relation to the intended tissue [8,12,15,18]. Drug loading and release must be interpreted in relation to the intended tissue. Rapid release may be useful for acute inflammation but insufficient for chronic disease. Very slow release may prolong exposure but fail to reach a useful local level [12,18]. For wound and skin applications, release data should be coupled with deposition or tissue-retention data rather than interpreted only from bulk medium measurements [12,14,16,35,36].

The polymeric matrix must also be compatible with OA physical properties. Because OA is lipophilic and poorly water-soluble, direct dispersion in hydrophilic matrices may lead to poor uniformity, crystallization, or incomplete release [1,5,8]. Nanocarrier-in-matrix systems may therefore be preferable for many local applications [8,12,14,15,18].

Biological controls are essential. Blank matrices, free OA, OA-loaded nanocarriers without matrix, and full nanocarrier-in-matrix systems should be compared where possible [12,14,16,35,36,37,38,39]. These controls help distinguish the effects of OA from the effects of the polymeric matrix, nanocarrier, hydration, occlusion, or local mechanical environment.

### 7.10. Translational Challenges of Hydrogels, Dressings, and Depots

Hydrogels, polymeric wound dressings, fiber membranes, and local depots are clinically attractive, but they also introduce practical development challenges. These include sterilization, shelf-life, mechanical stability, reproducible drug loading, polymer degradation, residual solvent, microbial contamination, patient handling, packaging, and regulatory classification [18,35,36,37]. For OA, additional concerns arise from poor solubility, crystallization risk, and vehicle-dependent biological activity [1,5].

Wound dressings and topical gels must be evaluated for irritation, sensitization, permeability, microbial compatibility, and performance under realistic skin or wound conditions. In vitro release testing alone is insufficient because wound exudate, enzymes, proteins, salts, variable pH, bacterial contamination, and tissue remodeling can influence polymer swelling, degradation, OA partitioning, and carrier stability. Progressive testing in more realistic models is therefore important. [35,36,37,38,39].

Intra-articular depots have a different translational profile. Joint injections require sterility, low endotoxin burden, appropriate viscosity, injectability through clinically relevant needles, and absence of long-term synovial irritation. A formulation that performs well in a rat model may still require substantial optimization before human translation because joint size, movement, clearance, mechanical loading, and dosing schedules differ substantially [18].

For clinical relevance, local OA systems should be compared not only with free OA, but also with existing standards of care where possible [12,18,36]. In inflammatory skin disease, this may include corticosteroids, calcineurin inhibitors, or approved topical therapies [12]. In osteoarthritis, comparison with hyaluronic acid, corticosteroids, NSAID-based approaches, or other intra-articular therapies may be appropriate [18]. In wound healing, advanced dressings or standard wound-care materials provide more meaningful comparators than free OA alone [35,36,37].

### 7.11. Local-Delivery Implications

Hydrogels, polymeric wound dressings, fiber membranes, and local depot systems are important for OA delivery because they shift the formulation goal from systemic bioavailability to controlled local exposure [8,12,15,18]. Their value lies in combining OA dispersion with tissue residence, hydration, mechanical contact, and route-specific release.

The most convincing local OA systems are those in which the carrier and matrix have separate but complementary functions. The nanocarrier improves OA dispersion or incorporation, while the hydrogel, dressing, fiber membrane, or thermosensitive depot controls placement, residence, and interaction with the tissue environment. For OA, this local formulation logic is especially relevant to inflammatory skin disease, wound healing, dermal delivery, osteoarthritis, and other tissue-restricted inflammatory conditions [8,12,14,15,16,18,36,37].

## 8. Polymeric Microneedles and Transdermal Delivery Platforms

### 8.1. Why Microneedles Are Relevant to OA Polymeric Delivery

Polymeric microneedles are relevant to OA delivery because they can bypass the stratum corneum while preserving the advantages of local, minimally invasive administration [16,32,33,40]. This is important for OA because passive topical delivery is limited by poor aqueous solubility, formulation-dependent exposure, and restricted skin penetration [1,5]. By creating transient microchannels or depositing drug-loaded polymeric material into the skin, microneedles can increase intradermal access without requiring systemic administration.

For OA, microneedles are most useful when they are treated as part of an integrated delivery platform rather than as standalone devices [16,32,33,40]. A microneedle array may solve the barrier problem, but it does not automatically solve OA solubility, crystallinity, dose loading, or release control. These formulation barriers may still require polymeric nanoparticles, micelles, nanogels, cyclodextrin complexes, hydrogels, or hyaluronic-acid-based nanocarriers embedded within or applied after the microneedle system [7,9,12,14,15,17].

This carrier-in-microneedle logic is particularly appropriate for OA. The microneedle controls skin access and deposition depth, whereas the embedded carrier controls drug dispersion, physical state, and release after insertion [7,9,12,14,15,16,17,32,33,40]. In this sense, microneedles should be viewed as a route-enabling platform for OA rather than simply as a device for pushing more drug through the skin.

The field should nevertheless be framed cautiously. Direct reports of OA-loaded microneedles remain limited, and much of the rationale comes from the convergence of OA delivery barriers, polymeric OA nanocarrier studies, and broader microneedle literature in transdermal drug delivery [1,5,7,9,12,14,15,16,17,32,33,40]. Microneedles are therefore a plausible future direction for OA, but not yet an established OA formulation class.

### 8.2. Types of Microneedles and Relevance to OA

Microneedles are commonly classified as solid, coated, hollow, dissolving, and hydrogel-forming systems [16,32,33,40]. Each type creates a different delivery mechanism, and their relevance to OA depends on whether the system can maintain the drug in a dispersed, deliverable, and biologically useful form.

Solid microneedles mainly pretreat the skin by forming microchannels, after which a topical formulation is applied. This strategy could improve OA delivery if the post-treatment formulation is an OA-loaded gel, micelle, nanogel, nanoparticle dispersion, or inclusion-complex system [1,5,9,12,14,15]. However, the approach still depends on diffusion from an external formulation into transient skin pathways, so OA must remain bioavailable during and after application.

Coated microneedles deliver drug from a coating on the microneedle surface. This approach may be less suitable for OA if the coating volume limits dose loading or if OA crystallizes during coating and drying [1,5,40]. Coated systems could still be useful if OA is incorporated into a polymeric coating containing solubilizers, micelles, cyclodextrins, or nanocarriers, but coating uniformity, drying behavior, and dose reproducibility would become critical [9,16,17,32,33,40].

Hollow microneedles can inject liquid formulations through internal channels. They could theoretically deliver OA-loaded nanosuspensions, micelles, or nanoparticle dispersions, but viscosity, clogging, sterility, dose accuracy, and colloidal stability would be major constraints [7,9,12,14,15,17]. For OA, this approach may be more demanding than dissolving or hydrogel-forming systems because the injected formulation must remain stable and free of crystalline precipitates.

Dissolving microneedles are more attractive for OA because the polymeric needle matrix can incorporate drug-loaded carriers and then dissolve after insertion [16,32,33,40]. Rapid dissolution can deposit OA-containing material in the skin, while embedded nanoparticles, micelles, nanogels, or cyclodextrin complexes can provide additional control over dispersion and release [7,9,12,14,15,17]. Hydrogel-forming microneedles offer a related but distinct strategy: the needles swell after insertion and can act as conduits or controlled interfaces between an external reservoir and the skin [16,32,33,40].

Overall, dissolving and hydrogel-forming microneedles appear most compatible with OA polymeric delivery because they can integrate skin-barrier bypass with polymeric control of dose placement, release, and formulation stability [7,9,12,14,15,16,17,32,33,40].

### 8.3. Polymeric Materials for OA-Compatible Microneedles

Polymeric microneedles can be prepared from materials such as hyaluronic acid, polyvinylpyrrolidone, polyvinyl alcohol, carboxymethylcellulose, chitosan, gelatin, alginate, and other water-soluble or hydrogel-forming polymers [16,32,33,40]. Material selection determines mechanical strength, insertion efficiency, dissolution or swelling behavior, drug compatibility, residual moisture, and storage stability.

For OA, polymer choice must account for the poor aqueous compatibility of the payload [1,5]. Hydrophilic microneedle polymers may dissolve or swell well, but they may not solubilize OA efficiently on their own. Direct loading of crystalline OA into a hydrophilic needle matrix could lead to poor dose uniformity, slow or incomplete release, and unpredictable skin deposition. A nanocarrier-loaded design may therefore be more rational than direct drug loading [7,9,12,14,15,17].

Hyaluronic acid is especially relevant because HA-based OA nanoprodrugs have already been explored for topical psoriasis delivery [12]. Incorporating HA-based OA systems into dissolving or hydrogel-forming microneedles could provide a future strategy that combines polymer-mediated skin interaction, microneedle deposition, and responsive release. However, the degree of substitution, molecular weight, mechanical strength, dissolution rate, and repeated-dose skin tolerability would all require careful control [12,16,32,33,40,41].

Chitosan and other cationic polymers may also be useful because they can interact with biological membranes and may provide mucoadhesive or antimicrobial properties. However, cationic polymers can also increase irritation or cytotoxicity, which is important for OA systems intended for inflamed or damaged skin [16,32,33,40]. Polymer selection should therefore balance mechanical performance with skin compatibility and formulation stability.

### 8.4. Nanocarrier-Loaded Microneedles

Nanocarrier-loaded microneedles are one of the most logical microneedle strategies for OA. The nanocarrier addresses OA solubility, dispersion, physical state, and release, while the microneedle enables deposition into or across the skin barrier [7,9,12,14,15,16,17,40]. This combined approach is more appropriate than attempting to load poorly soluble crystalline OA directly into a dissolving polymeric needle.

Potential OA-compatible carriers include PLGA nanoparticles, PEGylated PLA/PLGA nanoparticles, polymeric micelles, cyclodextrin complexes, hyaluronic-acid-based nanoprodrugs, nanogels, and polymer-assisted lipid systems [5,7,9,12,14,15,17]. Each carrier type would behave differently after microneedle insertion. Polymeric micelles may release OA relatively quickly after dilution, whereas PLGA nanoparticles, nanogels, or depot-forming systems may support slower release [7,9,14,15,17].

Embedding nanocarriers into microneedles adds another processing step and another source of variability. Casting, molding, drying, humidity, polymer concentration, backing-layer formation, and mechanical compression can affect both microneedle geometry and nanocarrier stability [16,32,33,40]. For OA-loaded carriers, these steps may also change drug crystallinity, aggregation, release rate, or carrier recovery [1,5,7,9,12,14,15,17].

Therefore, nanocarrier-loaded OA microneedles should be characterized at several stages: before incorporation into the microneedle matrix, after drying and storage, after insertion or dissolution, and after release from the matrix. Important quality attributes include microneedle geometry, mechanical strength, insertion depth, dissolution or swelling behavior, nanocarrier recovery, OA content uniformity, release kinetics, residual moisture, and storage stability [16,32,33,40].

### 8.5. Microneedles for Inflammatory Skin Diseases and Psoriasis

Inflammatory skin diseases, including psoriasis, are among the most plausible targets for OA microneedle delivery. OA has reported anti-inflammatory activity, and HA-based OA nanoparticles have already been developed for topical psoriasis treatment [1,5,12]. Microneedles could strengthen this direction by delivering OA-loaded carriers into viable skin layers and reducing reliance on passive penetration through the stratum corneum [12,16,32,33,40].

For psoriasis and related inflammatory skin diseases, microneedles may offer several advantages: barrier bypass, localized deposition, reduced need for chemical penetration enhancers, and the possibility of patient-friendly patch-based administration [16,32,33,40,41]. These features are especially relevant for hydrophobic compounds such as OA, which do not readily cross the skin barrier in free form [1,5,12].

However, inflamed skin is not equivalent to healthy skin. Barrier disruption, immune-cell infiltration, altered cytokine expression, increased vascularity, scaling, and local sensitivity can all influence microneedle insertion, drug deposition, and tolerability. OA microneedle systems for psoriasis should therefore be evaluated in disease-relevant skin models rather than only in healthy excised skin, synthetic membranes, or simple release media [12,16,41].

A realistic development pathway would compare free OA gel, OA micelles, HA-based OA nanoparticles, dissolving microneedles containing OA carriers, and nanocarrier-loaded hydrogel-forming microneedles in the same or comparable skin models. Such comparisons would clarify whether microneedles provide added value beyond improved solubilization [12,14,16,40,41].

### 8.6. Microneedles for Wound Healing and Tissue Repair

Microneedle systems may also be relevant to wound healing and tissue repair, but this application requires a cautious frame. OA has been associated with wound-healing activity, epithelial cell migration, anti-inflammatory effects, and oxidative-stress modulation [1,5,36,38,39]. However, wounds are dynamic environments, and the timing, dose, depth, and location of delivery are critical.

Direct microneedle application to open wounds may not always be appropriate because damaged tissue can be painful, fragile, infected, highly inflamed, or irregular in structure [36]. OA microneedle systems may be more suitable for periwound skin, chronic wound edges, scar modulation, or controlled intradermal delivery adjacent to damaged tissue [16,36,40]. For open wound surfaces, hydrogels, fiber membranes, and nanocarrier-loaded dressings may be more appropriate than microneedle patches [8,14,15,36].

If OA microneedles are developed for wound-related applications, the polymeric matrix must be designed for biocompatibility, moisture control, minimal irritation, and appropriate mechanical behavior [16,32,33,36,40]. The formulation should also consider whether OA release supports migration, reduces excessive inflammation, or affects remodeling in a stage-specific manner. Excessive or poorly timed delivery could be counterproductive if it interferes with normal inflammatory or proliferative phases of healing.

Relevant evaluation endpoints should include keratinocyte and fibroblast viability, cell migration, inflammatory cytokines, oxidative-stress markers, collagen deposition, wound closure, scar-related markers, infection risk, and tissue histology [36,38,39]. These outcomes are more informative than release data alone because they connect OA deposition to tissue repair.

### 8.7. Microneedles and OA Penetration-Enhancer Logic

A distinct but relevant part of OA transdermal research concerns OA derivatives as penetration enhancers. Bednarczyk-Cwynar et al. synthesized simple OA amides and evaluated them as potential transdermal penetration enhancers. This work is not a polymeric nanocarrier study, but it shows that OA-based structures can interact with skin permeation processes [42].

This literature supports the broader idea that OA and its derivatives may be relevant to skin delivery not only as therapeutic payloads, but also as molecules capable of modifying barrier interaction. In the context of polymeric microneedles, this raises an interesting possibility: OA derivatives or OA-loaded carriers could be designed to combine therapeutic activity with controlled skin interaction [16,40,42].

This possibility should be treated carefully. Penetration enhancement may increase permeation, but it can also disturb barrier function or increase irritation risk. In inflamed skin, psoriasis, or wound-associated tissue, barrier integrity may already be compromised, making safety evaluation particularly important [16,41,42].

For OA microneedle systems, physical barrier bypass through microneedles may provide a more controllable strategy than aggressive chemical penetration enhancement alone. The most balanced approach may combine shallow, localized microneedle deposition with polymeric control over OA release and carrier stability [16,32,33,40,42].

### 8.8. Controlled Release from Polymeric Microneedles

Controlled release from OA-loaded microneedles can be engineered through polymer dissolution, hydrogel swelling, nanocarrier release, polymer degradation, drug diffusion, and carrier–matrix interactions. This multilevel control is attractive because OA release can be regulated by both the microneedle matrix and the embedded carrier [7,9,12,14,15,16,17,32,33,40].

For dissolving microneedles, release may occur rapidly after insertion if the polymeric matrix dissolves quickly [16,32,33,40]. This may be useful for short-term local delivery but may not sustain OA exposure. Incorporating PLGA nanoparticles, nanogels, or other sustained-release carriers into dissolving microneedles could create a two-step system: rapid needle dissolution followed by slower OA release from the deposited nanocarrier [7,9,14,15,17].

For hydrogel-forming microneedles, the swollen polymer network can act as a conduit or controlled-release interface. These systems could be paired with OA reservoirs, nanoparticle dispersions, micellar systems, or soft polymeric carriers to support longer delivery periods. The key challenge is ensuring that OA or the OA-loaded carrier partitions effectively from the reservoir through the hydrogel network into the skin [7,9,12,14,15,16,17,32,33,40].

Release testing should match the microneedle mechanism. Simple immersion of a patch in buffer may not reflect skin insertion, swelling pressure, interstitial-fluid composition, polymer dissolution, or local tissue uptake [16,32,33,40]. OA microneedle studies should therefore include skin-mimicking membranes, ex vivo skin, reconstructed skin, or disease-relevant skin systems where possible [12,16,40,41].

### 8.9. Manufacturing and Quality Attributes

Polymeric microneedles introduce manufacturing and quality-control requirements that go beyond conventional nanocarrier characterization. Important attributes include needle height, base width, tip sharpness, array density, mechanical strength, insertion efficiency, dissolution or swelling time, dose uniformity, residual moisture, and packaging stability [16,32,33,40].

For OA-loaded microneedles, additional quality attributes include OA loading, drug distribution within the needles, nanocarrier stability after drying, crystallization risk, release profile, and skin deposition [1,5,7,9,12,14,15,17]. If OA-loaded nanoparticles or micelles are embedded in the microneedle matrix, the carrier should be characterized before incorporation, after drying, after storage, and after release from the dissolved or swollen patch [7,9,12,14,15,17].

Manufacturing methods such as micromolding, casting, centrifugation-assisted filling, vacuum-assisted filling, drying, and backing-layer formation can affect payload distribution and mechanical performance [16,32,33,40]. For OA, drying conditions are especially important because solvent removal and water loss may promote drug crystallization, carrier aggregation, or changes in release behavior [1,5,7,9,12,14,15,17].

Scale-up is a major translational issue. Microneedle patches must be produced with reproducible geometry, mechanical strength, microbial control or sterility when required, and dose consistency [16,32,33,40]. For OA systems intended for clinical use, scalable fabrication and packaging are as important as biological activity because inconsistent microneedles may lead to variable dose delivery and tissue exposure.

### 8.10. Safety and Patient-Oriented Considerations

Microneedle systems are often described as minimally invasive and patient-friendly, but safety and acceptability must still be demonstrated. Potential concerns include local irritation, erythema, incomplete insertion, broken tips, infection risk, polymer residue, allergic reactions, and dose variability. These concerns are especially relevant for chronic skin diseases, where repeated application may be required [16,32,33,40,41].

For OA-loaded microneedles, blank microneedle controls are essential because the polymeric matrix itself can influence skin response [16,32,33,40]. If nanocarriers are included, blank nanocarrier-loaded microneedles should also be tested. This is necessary to distinguish OA effects from polymer effects, nanocarrier effects, hydration, occlusion, mechanical puncture, and local inflammatory responses [7,9,12,14,15,16,17,32,33,40].

Patient-oriented factors also matter. A microneedle OA patch for chronic inflammatory skin disease would need to be easy to apply, stable during storage, acceptable on visible skin areas, minimally painful, non-irritating, and compatible with repeated use [16,32,33,40,41]. If microneedles are intended for periwound or scar-related applications, comfort, dressing compatibility, infection risk, and ease of use become additional design constraints [16,32,33,40].

### 8.11. Translational Positioning and Research Priorities for OA Microneedle Systems

OA microneedle delivery should be positioned as a promising but still emerging direction rather than an established formulation class. The mechanistic rationale is strong: OA is poorly soluble, passive skin delivery is limited, polymeric carriers can improve dispersion and control release, and microneedles can bypass the stratum corneum [1,5,7,9,12,14,15,16,17,32,33,40]. However, direct studies of OA-loaded microneedle systems remain necessary to demonstrate formulation feasibility and therapeutic added value.

For a poorly soluble compound such as OA, nanocarrier-loaded dissolving or hydrogel-forming microneedles may be more rational than direct incorporation of crystalline drug. Such systems could combine physical barrier bypass with controlled deposition and sustained release from an embedded nanoparticle, micelle, nanogel, or reservoir formulation [7,9,12,14,15,16,32,33].

The most plausible applications are local and skin-oriented, including inflammatory skin disease, psoriasis, localized dermatitis, scar-related conditions, and possibly periwound tissue modulation [12,16,36,37,38,39,40,41]. Development should assess needle geometry, mechanical strength, insertion efficiency, dose uniformity, dissolution or swelling, OA physical state after drying, skin deposition, release, irritation, barrier recovery, inflammatory response, repeated-dose tolerability, and patient acceptability.

Comparative studies should evaluate free OA gels, OA micelles or nanoparticles, OA-loaded dissolving microneedles, and nanocarrier-loaded microneedles within the same skin- or disease-relevant model. Such comparisons are necessary to establish whether microneedles provide a measurable benefit beyond improved solubilization or conventional topical administration [12,14,16,32,33,40,41].

At present, the evidence supports OA microneedles as a future-oriented polymer-assisted delivery platform. Advancement will require direct OA formulation studies demonstrating safety, reproducibility, target-site exposure, and an advantage over simpler topical or local alternatives.

**Figure 4:**
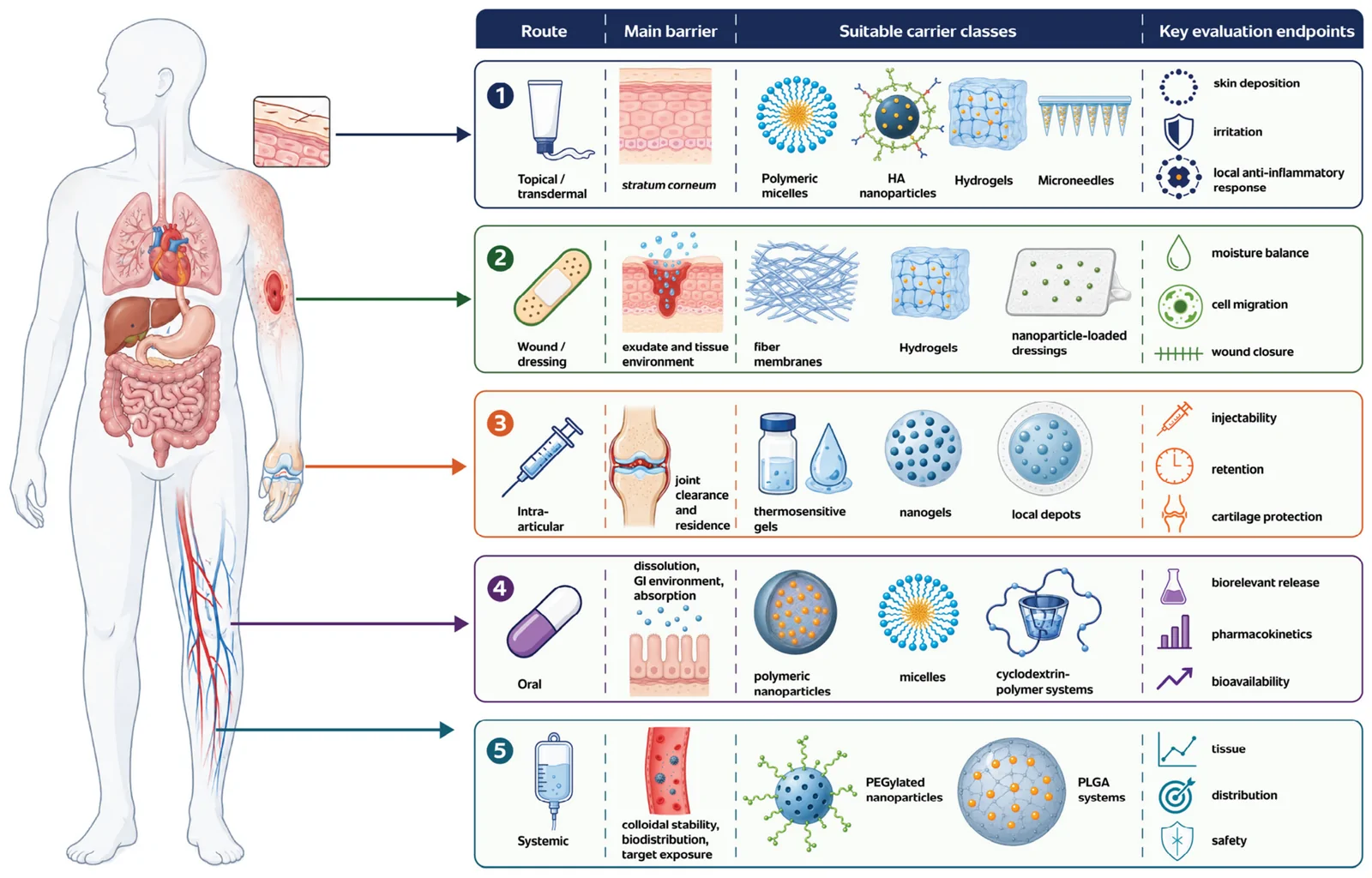
Route-specific positioning of polymeric and polymer-assisted OA delivery platforms. The numbered sections represent: (1) topical and transdermal delivery; (2) wound and dressing-based delivery; (3) intra-articular delivery; (4) oral delivery; and (5) systemic delivery. For each route, the scheme links the main biological barrier with suitable carrier classes and key evaluation endpoints. This route-specific view helps distinguish general formulation feasibility from application-oriented delivery design.

The route-specific positioning of local, transdermal, intra-articular, oral, and systemic OA delivery platforms is summarized in Figure 4.

Local, transdermal, and microneedle-compatible OA delivery platforms are compared in Table 4.

## 9. Characterization and Critical Quality Attributes of Oleanolic-Acid-Loaded Polymeric Nanocarriers and Polymer-Assisted Delivery Platforms

### 9.1. Why Characterization Is Central for OA Polymeric Nanocarriers and Polymer-Assisted Platforms

For OA-loaded polymeric nanocarriers and polymer-assisted delivery system platforms, characterization is not a supplementary technical step. It determines whether a formulation can be interpreted scientifically and whether it has any realistic development value. OA is poorly soluble, lipophilic, crystalline or semi-crystalline, and strongly formulation-dependent; therefore, small differences in carrier composition, preparation method, drug physical state, and release environment may substantially change biological response [1,5,7,8,9].

A formulation should not be considered successful only because OA can be incorporated into a nanosystem. The more important question is whether the system can be reproduced, stored, characterized, and shown to release OA in a form relevant to the intended route [5,6,7,8,9,13]. This is especially important for polymeric carriers because polymer molecular weight, degradation behavior, surface chemistry, matrix architecture, and drug–polymer compatibility can all affect performance [6,7,8,9,13].

Critical quality attributes should therefore be defined according to the target product profile. An oral polymeric nanoparticle, a topical micellar system, a wound dressing, an intra-articular thermosensitive gel, and a microneedle-loaded nanocarrier do not require identical performance criteria [8,12,15,32,33]. However, all require a rational connection between composition, structure, OA physical state, release behavior, stability, safety, and biological response [6,7,8,9,13].

**Figure 5:**
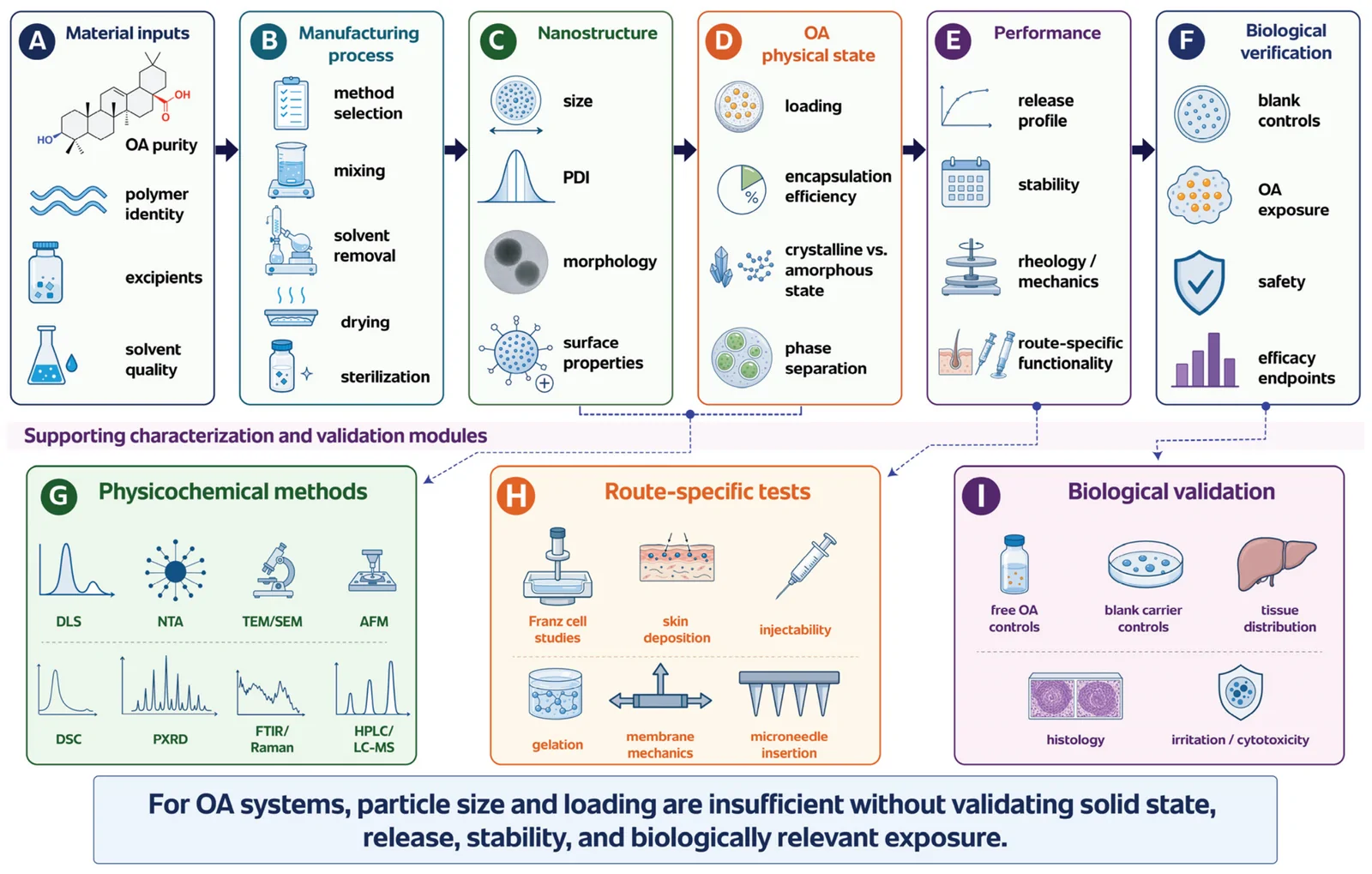
Characterization workflow and critical quality attributes for OA-loaded polymeric nanocarriers and polymer-assisted delivery platforms. Panels (**A**–**F**) present the sequential characterization workflow: (**A**) material inputs, including OA purity, polymer identity, excipients, and solvent quality; (**B**) manufacturing process, including method selection, mixing, solvent removal, drying, and sterilization; (**C**) nanostructure, including particle size, PDI, morphology, and surface properties; (**D**) OA physical state, including drug loading, encapsulation efficiency, crystalline or amorphous state, and phase separation; (**E**) formulation performance, including release profile, stability, rheology or mechanical properties, and route-specific functionality; and (**F**) biological verification, including blank controls, OA exposure, safety, and efficacy endpoints. Panels (**G**–**I**) represent parallel supporting evaluation modules rather than subsequent workflow stages: (**G**) physicochemical characterization methods used primarily to assess nanostructure, OA physical state, and composition; (**H**) route-specific performance tests, including skin, injectable, gel, membrane, and microneedle assessments; and (**I**) biological validation, including appropriate controls, tissue distribution, histology, irritation, and cytotoxicity. Arrows between panels (**A**–**F**) indicate the sequential workflow, whereas the connections to panels (**G**–**I**) indicate the analytical and validation methods supporting the relevant workflow stages.

The characterization workflow and critical quality attributes for OA-loaded polymeric and polymer-assisted systems are summarized in Figure 5.

For OA, characterization must also distinguish total drug content from biologically available drug. A system may contain a high amount of OA but release it too slowly, retain it within a hydrophobic matrix, or contain crystalline drug domains that are poorly available under biological conditions [1,5,7,8,9]. For this reason, OA nanocarrier evaluation should combine conventional nanocarrier characterization with compound-specific assessment of physical state, release, and exposure [1,5,6,7,8,9,13].

### 9.2. Particle Size, Size Distribution, and Polydispersity

Particle size is one of the most commonly reported parameters for polymeric nanocarriers because it influences colloidal behavior, tissue penetration, cellular uptake, biodistribution, clearance, and release kinetics [6,7,13]. For OA delivery, size can also affect surface area available for release and the likelihood that the compound remains dispersed rather than precipitated [1,5,7,8,9].

Dynamic light scattering is widely used to measure hydrodynamic diameter and polydispersity index, but DLS data require careful interpretation. The method is sensitive to aggregates, large particles, and mixed populations because it provides intensity-weighted measurements that may overrepresent larger species [6,13]. In OA-loaded systems, undetected aggregates, drug crystals, or carrier clusters may distort apparent size and affect biological performance [1,5,7,8,9].

Polydispersity is particularly important for development. A narrow size distribution suggests better process control, whereas broad or multimodal distributions may indicate uncontrolled nucleation, aggregation, mixed carrier populations, or incomplete stabilization [7,13]. For OA, high polydispersity may also reflect coexistence of nanocarriers with free drug aggregates, especially when drug loading exceeds the capacity of the carrier matrix [1,5,7,8,9].

Particle-size analysis should therefore be supported by complementary methods where possible. Nanoparticle tracking analysis, electron microscopy, atomic force microscopy, or field-flow fractionation can help distinguish true nanoscale carriers from aggregates, micelles, fibers, vesicular structures, or gel-associated particles [6,7,12,13,15]. This is particularly important for hybrid OA systems that contain both nanoscale carriers and polymeric matrices [8,18]

### 9.3. Surface Charge and Zeta Potential

Zeta potential is frequently used as an indirect indicator of colloidal stability and surface properties. For OA-loaded polymeric nanocarriers, surface charge may influence aggregation, protein adsorption, interaction with mucus or skin, cellular uptake, and compatibility with biological fluids [6,7,13].

However, zeta potential should not be treated as a universal predictor of stability or biological performance. Sterically stabilized systems, such as PEGylated nanoparticles or polymeric micelles, may show relatively low absolute zeta-potential values but remain stable because of their hydrated polymeric surface layers [6,7,13,14]. Conversely, a high zeta potential measured in simple buffer does not guarantee stability in serum, wound exudate, synovial-like media, or skin fluids [12,13,18].

Surface properties are especially important when polymers such as PEG, hyaluronic acid, chitosan, poloxamers, or other functional excipients are used. These materials can modify hydration, protein adsorption, receptor interaction, tissue retention, and cellular uptake [7,12,13,14,18]. For this reason, zeta potential should be interpreted together with stability in relevant media, surface composition, release behavior, and biological response.

A platform- and route-specific overview of critical quality attributes, OA-specific failure risks, and recommended characterization methods is provided in Table 5.

### 9.4. Morphology and Carrier Architecture

Morphological characterization is essential because carrier architecture can strongly affect OA loading, release, stability, and biological interaction. Microscopy can help determine whether a formulation contains spherical nanoparticles, polymeric micelles, liquid crystalline particles, nanogels, fibers, vesicle-like structures, aggregates, or mixed populations [8,9,12,15,18,33].

Electron microscopy provides direct visual information on shape, surface structure, aggregation, and size distribution. Transmission electron microscopy and scanning electron microscopy are particularly useful for nanoparticles and fiber membranes, whereas cryogenic electron microscopy may be valuable for soft or hydrated systems when available [6,7,13]. For OA-loaded PLGA fiber membranes, morphology is especially important because fiber diameter, porosity, and surface structure can influence drug release and tissue contact [8].

For hydrogels, nanogels, and thermosensitive depots, morphology must be linked with macroscopic behavior. A gel-based OA formulation cannot be characterized only by nanoparticle size because gel network structure, rheology, swelling, and depot integrity also determine release and residence time [15,18,33]. This is particularly relevant for topical and intra-articular systems, where the formulation must remain at the application site.

Morphological analysis can also reveal formulation failure. If OA crystallizes outside the carrier or forms large aggregates, average DLS values may still suggest nanoscale size while the formulation contains poorly controlled drug domains. Morphology should therefore be interpreted together with solid-state analysis, drug loading, and release behavior [1,5,6,7,8,9,13].

### 9.5. Drug Loading and Encapsulation Efficiency

Drug loading and encapsulation efficiency are central parameters for OA nanocarriers because they describe how much compound is present in the final system and how efficiently the preparation process incorporates OA [1,5,7]. However, these values are not sufficient on their own to establish formulation quality.

For hydrophobic compounds such as OA, apparent encapsulation may include several drug populations: OA molecularly dispersed within the polymer matrix, OA associated with the carrier surface, OA trapped in amorphous domains, OA contained in hydrophobic micellar or lipidic regions, and crystalline or aggregated OA that remains after purification [1,5,7,8,9]. These populations may behave differently during storage, dilution, release testing, and biological exposure.

OA nanocarrier studies should therefore distinguish, where possible, between total OA content, encapsulated OA, surface-associated OA, free or precipitated OA, and released OA [7,8,9,13]. This is especially important when comparing different polymers, preparation methods, stabilizers, or dosage forms. Two systems with similar encapsulation efficiency may have very different release profiles and biological effects if OA is present in different physical states.

Validated analytical methods are essential. HPLC or LC–MS methods should quantify OA after complete extraction and after separation of free drug from the carrier fraction [8,12]. The extraction method should be able to recover OA from polymeric matrices, hydrogels, micelles, fibers, or microneedle materials without degradation or incomplete release from the sample [8,12,15,16]. Drug loading data should also be interpreted alongside solid-state analysis and release testing.

### 9.6. Physical-State Assessment

Physical-state assessment is particularly important for OA because the compound is poorly soluble and may exist in crystalline, semi-crystalline, amorphous, molecularly dispersed, surface-associated, or phase-separated forms [1,5,7,8,9]. These states can strongly influence release, storage stability, precipitation after dilution, and biological exposure.

A high-loading OA system may be weak from a formulation standpoint if excess drug crystallizes during preparation, drying, storage, or application [1,5,17,19]. This risk is relevant for PLGA nanoparticles, electrospun fiber membranes, polymeric micelles, dried microneedles, nanocarrier-loaded gels, and thermosensitive depots [8,9,14,15,16,18]. Loading must therefore be evaluated together with physical state, not separately from it.

Differential scanning calorimetry, powder X-ray diffraction, Fourier-transform infrared spectroscopy, Raman spectroscopy, polarized-light microscopy, and electron microscopy can help determine whether OA is crystalline, amorphous, molecularly dispersed, or phase-separated [8,9,13]. For hydrated systems such as hydrogels or nanogels, sample preparation may alter the formulation, so complementary methods and careful interpretation are required [15,33].

Physical-state assessment should also be included in stability studies. OA may be initially dispersed within a carrier but recrystallize during drying, lyophilization, humidity exposure, temperature cycling, or long-term storage [1,5,7,8,9]. Monitoring drug state over time is therefore more informative than measuring it only immediately after preparation.

### 9.7. Release Testing and Exposure Verification

Release testing is one of the most important characterization steps for OA-loaded polymeric systems. Because OA is poorly soluble and hydrophobic, release results can vary substantially depending on sink conditions, surfactant concentration, protein content, medium composition, agitation, membrane selection, and separation method [1,5,7,8,9,13]. A release method that is convenient analytically may not reflect the biological environment of the intended route.

Simple aqueous buffer may underestimate OA release if sink conditions are not maintained. In contrast, excessive surfactant or organic cosolvent may overestimate the fraction that would be biologically available in vivo [1,5,7,8,9]. Release media should therefore be selected according to the route and justified mechanistically.

Route relevance is essential. Skin-oriented systems may require skin-compatible receptor media, ex vivo deposition studies, reconstructed skin, or permeation models [12,14,15,16]. Wound systems should consider wound-exudate-like conditions, including proteins, salts, enzymes, and pH variation [7,35,36,37,38]. Intra-articular depots should consider synovial-fluid-like environments and prolonged residence [18]. Oral systems should be tested under biorelevant gastrointestinal conditions [1,5]. Systemic nanoparticles may require protein-containing media and dilution stability testing [7,9,13].

Exposure verification is equally important. Biological activity should not be interpreted only from the nominal OA concentration added to cells, tissues, or animals. Whenever possible, OA should be measured in release medium, cells, skin layers, wound fluid, synovial-like compartments, or tissue extracts [15,16,18,33]. This helps determine whether the carrier changes actual OA availability or only the nominal dose.

### 9.8. Stability During Storage and Biological Dilution

Stability studies are essential for OA-loaded polymeric systems because both the drug and the carrier can change over time. Relevant instability pathways include aggregation, drug crystallization, polymer degradation, micelle dissociation, gel syneresis, drug leakage, water uptake, drying-related structural changes, and loss of mechanical integrity [1,5,7,8,9,13,14,15,18].

For nanoparticles, stability should include size, PDI, zeta potential, aggregation, drug loading, release profile, and drug physical state after storage [7,8,9,13]. For hydrogels and thermosensitive depots, rheology, gelation behavior, phase separation, water loss, and release should be monitored [15,18]. For fiber membranes and microneedles, mechanical integrity, residual moisture, drug distribution, and dose uniformity are especially important [8,16,18,32].

Stability conditions should reflect the intended product form. A freshly prepared laboratory dispersion, a lyophilized powder, a dried microneedle patch, a hydrated gel, and a packaged wound dressing have different risks [8,12,18,32,33]. For OA, humidity and temperature may be particularly important because they can influence crystallization, polymer relaxation, and release behavior [1,5,8,9].

A stable formulation should not be defined only by the absence of visible precipitation. It should maintain critical quality attributes that are linked to performance: drug content, physical state, release profile, carrier integrity, and biological activity where relevant [7,8,9,13,14,15,16,18].

### 9.9. Residual Solvents, Raw-Material Quality, and Chemical Stability

Many OA-loaded polymeric nanocarriers are prepared using organic solvents, nanoprecipitation, emulsification, electrospinning, or casting methods [7,8,9,16]. Residual solvents can influence safety, polymer behavior, drug crystallization, and biological response. They should therefore be measured and controlled, especially in systems intended for local, topical, intra-articular, or transdermal use [7,8,9,13,16].

OA raw material quality should also be considered. Purity, polymorphic form, residual solvents, impurity profile, botanical or synthetic origin, and storage conditions may affect loading, crystallization, and release [1,5]. In polymer-assisted systems, excipients such as PEGylated polymers, hyaluronic acid, poloxamers, cyclodextrins, chitosan, surfactants, and hydrogel-forming polymers may also contribute impurities, degradation products, or batch variability [12,14,15,18].

Chemical stability should be monitored for both OA and the carrier. Polymer degradation may change pH, release behavior, and drug stability, while responsive linkages such as disulfide bonds may degrade under storage or biological conditions [12,13,18]. Stability-indicating analytical methods are therefore preferable to simple content assays whenever the formulation is intended for development beyond proof of concept.

### 9.10. Route-Specific Critical Quality Attributes

A critical quality attribute should be defined by its potential influence on safety, exposure, release, or clinical performance for the intended route. The same numerical result may therefore have different significance in different OA formulations. For example, particle size is central to the colloidal stability and biodistribution of an injectable nanoparticle, but it does not by itself determine the usability of a topical gel, wound dressing, intra-articular depot, or microneedle patch.

For oral OA systems, the most important attributes include dispersion after gastrointestinal dilution, precipitation risk, release in biorelevant media, stability during pH change, and interaction with bile salts, digestive components, and food [1,5,19,20]. An oral carrier that shows high encapsulation efficiency but releases OA only under non-physiological sink conditions may have limited relevance for bioavailability enhancement.

Systemic or intravenous nanoparticles require a different quality profile. Sterility, endotoxin burden, aggregation after dilution, stability in serum, protein interaction, free OA content, release, pharmacokinetics, biodistribution, and systemic safety become central [7,9,13,25,43]. For these systems, a narrow particle-size distribution is useful only when it remains stable under clinically relevant dilution and biological conditions.

Topical and dermal systems should be evaluated according to OA dose uniformity, rheology, spreadability, skin deposition, permeation, irritation, barrier compatibility, microbial quality, and release under skin-relevant conditions [12,14,15]. The desired endpoint should also be specified. A formulation intended for epidermal or dermal activity may require local retention rather than maximum transdermal permeation.

For wound dressings, additional critical attributes include matrix morphology, porosity, moisture management, exudate interaction, mechanical flexibility, adhesion, microbial risk, and compatibility with damaged tissue [8,35,36,37,38]. Intra-articular depots require injectability, gelation under physiological conditions, sterility, endotoxin control, joint residence, release in synovial-fluid-like media, local tolerability, and cartilage or synovial compatibility [15,18].

Microneedle systems require control of needle geometry, mechanical strength, insertion efficiency, dose uniformity, residual moisture, dissolution or swelling behavior, OA physical state after drying, skin deposition, irritation, and packaging stability [16,32,33]. In these products, nanoscale carrier properties must be evaluated together with device performance because successful nanocarrier preparation does not guarantee successful intradermal administration.

The route-specific priorities summarized in Table 4 should be incorporated into the quality target product profile from the beginning of development. Acceptance ranges should ultimately be linked to release, target-compartment exposure, biological response, and safety rather than selected only from generic nanocarrier conventions.

### 9.11. Biological Controls and Assay Interpretation

Biological evaluation is part of characterization for OA systems because the compound is strongly vehicle-dependent [1,5,17]. A stronger response from an OA-loaded carrier may result from improved dispersion, reduced precipitation, altered uptake, carrier-associated effects, or changed release kinetics rather than a change in intrinsic OA activity [7,9,12,17].

Appropriate controls are therefore essential. Studies should include blank carrier, free OA, solubilized OA, OA-loaded carrier, and, where relevant, carrier-in-matrix controls [7,8,9,12,14,15,16,17,18]. For responsive systems, non-responsive analogues or controls without cleavable linkages may help clarify whether the stimulus-responsive mechanism contributes to biological activity [12].

Assays should also match the intended application. Cancer-oriented systems require uptake, penetration, cytotoxic selectivity, and eventually three-dimensional or in vivo models [7,9]. Skin and psoriasis systems require keratinocyte, immune-cell, reconstructed skin, irritation, and deposition endpoints [12,14,15,16]. Wound systems require migration, inflammatory markers, oxidative stress, matrix deposition, and tissue repair endpoints [8,35,36,37,38]. Intra-articular systems require cartilage, synovium, inflammation, pain-related behavior, and local toxicity assessment [18].

Without these controls and application-relevant endpoints, it is difficult to determine whether an OA-loaded polymeric system has meaningful biological value or only produces a formulation-dependent assay effect.

### 9.12. Reproducibility, QbD Logic, and Minimum Characterization Set

Reproducibility is a major challenge for polymeric OA nanocarriers because preparation methods are sensitive to polymer batch, solvent composition, mixing rate, temperature, concentration, drying, and post-processing conditions. For OA, small process variations may change particle size, loading, drug physical state, release, and apparent biological activity [1,5,6,7,8,9,13].

A quality-by-design-oriented approach would be valuable for OA formulation development. Instead of optimizing one variable at a time, studies should define critical material attributes, critical process parameters, and critical quality attributes, then evaluate how formulation variables affect performance. This is especially relevant for PLGA nanoparticles, electrospun membranes, nanocarrier-loaded hydrogels, thermosensitive depots, and microneedle systems [6,7,8,9,12,13,15,18,33,39].

A minimum characterization set for OA-loaded polymeric nanoparticles should include particle size, size distribution, zeta potential, morphology, drug loading, encapsulation efficiency, physical-state assessment, release kinetics, storage stability, and biological controls [1,5,6,7,8,9,13]. For polymer-assisted gels, dressings, depots, and microneedles, additional attributes should include rheology, mechanical properties, dose uniformity, gelation or dissolution behavior, application performance, and route-specific safety [8,12,15,16,18,36,37].

At least one direct imaging method should complement DLS-based size analysis for nanoparticle systems. For fibers and microneedles, microscopy should confirm morphology, drug distribution, and structural integrity. For gels and depots, rheology and gelation behavior should be connected to release and residence-time expectations [8,15,18,32,33].

Release testing should be justified by the intended application rather than selected only for convenience. A method suitable for a PLGA nanoparticle suspension may not be appropriate for a topical gel, wound dressing, intra-articular depot, or microneedle patch [8,12,15,16,18]. Biological evaluation should similarly include both formulation controls and application-relevant models.

### 9.13. Characterization Priorities

Characterization of OA-loaded polymeric nanocarriers and polymer-assisted delivery platforms must go beyond routine particle size and encapsulation efficiency. Because OA is poorly soluble, lipophilic, and formulation-dependent, critical quality attributes should include drug physical state, release behavior, stability, exposure verification, carrier safety, and route-specific performance [1,5,6,7,8,9,13].

The central question is whether polymeric design produces reproducible and route-appropriate OA exposure. A high-loading nanoparticle, gel, dressing, depot, or microneedle system has limited value if OA crystallizes, releases unpredictably, destabilizes during storage, or produces biological effects that cannot be separated from carrier- or matrix-associated artifacts.

For OA, the most useful characterization strategy is a process–structure–function approach. Polymer composition and manufacturing conditions should be linked with carrier architecture, OA physical state, release kinetics, tissue exposure, biological response, and safety. This approach will make OA delivery studies more useful not only for this compound, but also for other poorly soluble natural products developed through polymeric nanocarrier systems [6,7,8,9,12,13,15,18,22,27,33,36].

## 10. Controlled Release and Mechanistic Delivery Considerations

### 10.1. Controlled Release as the Central Value of Polymeric OA Systems

Controlled release is one of the main reasons to develop polymeric and polymer-assisted nanocarriers for OA. Improving apparent solubility is necessary, but it is not sufficient. For OA, biological performance depends on how much drug becomes available, where it becomes available, and for how long exposure is maintained. A formulation that disperses OA transiently but releases it too quickly, too slowly, or unpredictably may have limited value despite favorable loading or particle-size data [1,5,7,8,9].

Polymeric systems can regulate OA availability through several mechanisms, including matrix diffusion, polymer swelling, hydrolytic degradation, erosion, micelle dissociation, gel relaxation, prodrug cleavage, responsive linker cleavage, and carrier–tissue interaction [12,14,15,16,17,18]. These mechanisms are especially relevant for a hydrophobic compound such as OA, which may partition into polymeric, lipidic, protein-rich, or tissue-associated environments rather than behave as a freely dissolved drug [1,5,7].

The release objective should be defined by the intended application. For oral delivery, release must support absorption in gastrointestinal conditions. For cancer-oriented nanoparticles, release should support cellular or intracellular exposure. For skin delivery, release should favor deposition in relevant skin layers. For wound dressings, release should match the tissue-repair environment. For intra-articular depots, release should maintain local joint exposure over time [7,8,9,12,14,15,16,17,18,33].

Controlled release should therefore not be treated as a generic formulation advantage. In OA delivery, the same release profile may be useful for one indication and unsuitable for another. The key question is whether the polymeric system produces a route-appropriate exposure pattern for the selected tissue and disease context [7,8,9,12,14,15,16,17,18,33].

### 10.2. Matrix Diffusion and Degradation in PLA/PLGA-Based OA Nanoparticles

PLA and PLGA nanoparticles release encapsulated drugs through overlapping mechanisms, including diffusion through the polymer matrix, water penetration, polymer swelling, pore formation, hydrolytic degradation, matrix erosion, and drug partitioning into the surrounding medium [1,5,7,9,25,44,45]. For OA-loaded PLA/PLGA systems, these mechanisms are further complicated by the strong hydrophobicity and poor aqueous solubility of the payload [1,5,7,9].

Early release may occur when OA is located near the particle surface, associated with loosely bound domains, or incompletely incorporated into the polymer matrix. A moderate initial release may be useful when rapid local exposure is needed, but excessive burst release can shorten the duration of action and increase local toxicity or irritation risk [25,44,45]. For OA, burst release may also indicate a formulation problem, such as surface-associated crystalline drug or incomplete purification, rather than a deliberate release design [1,5,7,9].

Sustained release is more likely when OA is distributed within the polymeric matrix and diffuses gradually as water penetrates the carrier and the polymer relaxes or degrades [7,9]. Polymer molecular weight, lactide ratio, end-group chemistry, particle size, drug loading, and drug physical state can all influence this process [13,25,44,45]. These variables should be interpreted together rather than as isolated formulation parameters.

For OA, a slow release profile is not automatically better. If the drug remains trapped in a hydrophobic matrix or crystalline domain, sustained release may become incomplete release. A well-designed PLA/PLGA OA system should therefore balance matrix retention with biologically useful drug availability [1,5,7,8,9,13].

### 10.3. Polymer Variables That Control OA Release

Polymer composition is a major determinant of release. In PLGA systems, a higher glycolide content generally increases hydrophilicity and may accelerate water uptake and degradation, whereas a higher lactide content usually increases hydrophobicity and may prolong release [12,13,25,45]. For OA, this balance is important because the compound itself is highly hydrophobic and may associate strongly with lactide-rich domains [1,5,7,9].

Polymer molecular weight also affects release. Lower-molecular-weight polymers may degrade faster and release drug more rapidly, whereas higher-molecular-weight polymers may form denser or more stable matrices with slower release [12,25,30,43]. The optimal molecular weight depends on whether the intended application requires rapid local exposure, prolonged tissue residence, or gradual systemic availability [15,16,17,18,33].

End-group chemistry can influence hydration, degradation, and drug–polymer interaction. Acid-terminated PLGA may promote water uptake and faster degradation compared with ester-capped PLGA, whereas more hydrophobic end groups may slow hydration and diffusion [12,27,30,35]. For a poorly soluble acidic triterpenoid such as OA, end-group chemistry may also affect the local microenvironment within the carrier and the strength of drug–polymer association [1,5,7,9].

Polymer blending and copolymer modification can provide additional release-control options. Blending PLGA with PEG, poloxamers, hyaluronic acid, chitosan, cyclodextrin-containing polymers, or hydrogel-forming materials may alter hydrophilicity, swelling, degradation, mucoadhesion, skin interaction, and release kinetics [12,13,14,15,17,18,28,29]. Such modifications should have a clear mechanistic purpose. Additional polymers should not be added only to make the formulation appear more advanced.

### 10.4. Drug Physical State and Burst Release

The physical state of OA inside a polymeric system strongly influences release. OA may be molecularly dispersed, amorphous, crystalline, surface-associated, trapped in hydrophobic domains, or present in phase-separated regions [1,5,7,9]. These states can produce very different release behaviors even when total drug loading appears similar.

Molecularly dispersed or amorphous OA may release more readily than crystalline OA, but it may also be less stable during storage. Crystalline OA may release slowly and incompletely because dissolution becomes the rate-limiting step. Surface-associated OA may produce rapid initial burst release, whereas deeply matrix-associated OA may remain unavailable for extended periods [1,5,7,8,9,13].

This issue is especially important for high-loading formulations. Increasing OA content may improve dose capacity, but once the carrier matrix is saturated, excess OA may crystallize or separate into drug-rich domains [1,5,8,9]. Such systems may show attractive loading values but poor release control and weak reproducibility.

Solid-state characterization should therefore be linked directly to release interpretation. Differential scanning calorimetry, X-ray diffraction, Raman spectroscopy, Fourier-transform infrared spectroscopy, and microscopy can help determine whether OA is crystalline, amorphous, molecularly dispersed, or interacting with the polymer matrix [8,9,13]. Without this information, release profiles may be difficult to interpret mechanistically.

### 10.5. Release from Soft and Responsive Polymeric Systems

Soft and responsive polymeric systems release OA through mechanisms that differ from rigid nanoparticle matrices. Polymeric micelles may release OA through dilution, unimer exchange, micelle dissociation, diffusion from the hydrophobic core, or partitioning into skin, proteins, or lipidic environments. For dermal OA delivery, micellar release must be balanced: the micelle should retain OA long enough to improve deposition, but release enough drug to support biological activity [14,31].

Nanogels and hydrogels can release OA through swelling, diffusion through hydrated polymer networks, erosion, degradation, or release of embedded nanocarriers [15,18,28]. Because OA is lipophilic, direct release from a hydrophilic gel may be inefficient unless the system contains hydrophobic domains, cyclodextrin complexes, lipidic nanostructures, micelles, or nanoparticles [12,14,15,18,28]. This makes carrier-in-gel systems particularly relevant for OA.

Responsive systems add another layer of control. Redox-responsive hyaluronic-acid-based OA nanoprodrugs can release OA through disulfide cleavage in reductive environments, while thermosensitive gels can regulate local residence through temperature-dependent gelation [12,18]. pH- or enzyme-responsive systems may also be useful in inflamed skin, wounds, tumors, or intracellular environments, but such responsiveness should be mechanistically justified and supported by route-relevant data [12,15,18,28].

For OA, responsiveness should not be decorative. A responsive bond, gel, or network is useful only if it improves local exposure, reduces premature release, increases tissue retention, or provides a safety advantage compared with a simpler formulation [12,15,18,28].

### 10.6. Release from Local Matrices, Depots, and Microneedle-Compatible Systems

Local OA delivery systems often involve more than one release-controlling level. In nanocarrier-in-gel, nanocarrier-in-fiber, nanocarrier-in-dressing, and nanocarrier-in-microneedle systems, both the embedded carrier and the macroscopic polymeric matrix influence release [8,12,15,18,28,32]. This multilevel structure can be valuable, but it also complicates interpretation.

In wound dressings and fiber membranes, OA release may depend on polymer degradation, fiber morphology, porosity, wound fluid penetration, protein binding, pH, enzymes, exudate composition, and tissue contact. The goal is not only to release OA into bulk medium, but to provide local exposure at a tissue surface undergoing inflammation and repair [8,35,36,37,38].

In intra-articular depots, release is linked to gelation, viscosity, joint residence, synovial-fluid interaction, mechanical movement, and clearance from the joint space. The OA thermosensitive nanogel for knee osteoarthritis illustrates this logic because the formulation is designed to maintain local joint exposure rather than maximize systemic absorption [18].

In microneedle-compatible systems, release may involve rapid dissolution of the needle matrix followed by slower release from deposited nanoparticles, micelles, nanogels, or polymeric reservoirs. Release testing for OA microneedles should therefore not rely only on dissolving patches in bulk buffer. More relevant evaluation would include insertion, skin deposition, local retention, recovery of embedded carriers, and release after placement in skin-like environments [16,32,33].

A consolidated comparison of release-control mechanisms across OA polymeric and polymer-assisted systems is provided in Table S1.

### 10.7. Sink Conditions, Release Media, and Mass Balance

Maintaining sink conditions is a major challenge in OA release testing because the compound is poorly soluble in aqueous media [1,5,7,9]. If the release medium cannot dissolve released OA, release may be underestimated. Conversely, excessive surfactant, ethanol, organic cosolvent, cyclodextrin, albumin, serum protein, lipoprotein, or lipid acceptor may artificially accelerate drug extraction from the carrier and overestimate biologically relevant release [1,5,7,9,17].

Release medium should therefore be chosen according to the intended application. Oral systems require gastrointestinally relevant conditions; topical systems require skin-compatible receptor media, ex vivo skin, reconstructed skin, or deposition models; wound dressings should consider wound-exudate-like media, pH changes, enzymes, salts, and proteins; intra-articular depots require synovial-fluid-like environments and prolonged testing [1,5,8,12,14,15,16,18,35,36,37,38].

For polymeric OA systems, release testing should ideally include mass balance. The amount of OA remaining in the carrier, released into the medium, adsorbed to the apparatus, retained in membranes, precipitated, or deposited in tissue should be quantified when possible [1,5,7,8,9]. This is particularly important because hydrophobic compounds can be lost to plasticware, filters, membranes, proteins, and biological matrices.

### 10.8. Modeling Release Kinetics

Mathematical modeling can help interpret OA release profiles, but it should be used cautiously. Models such as zero-order, first-order, Higuchi, Korsmeyer–Peppas, Weibull, and diffusion–erosion models can describe release data, but fitting alone does not prove a specific mechanism [7,8,9,25,44,45]. Mechanistic interpretation requires consistency between the model, polymer chemistry, carrier morphology, OA physical state, and experimental conditions.

For OA-loaded PLA or PLGA nanoparticles, release may involve both diffusion and polymer degradation. A Higuchi-type fit may suggest diffusion control, but it may not fully capture systems where water penetration, polymer erosion, matrix swelling, drug dissolution, or crystallinity changes contribute over time [7,8,9,13,25,30,44,45,46]. Modeling should therefore be supported by polymer degradation data, solid-state analysis, morphology, and stability information.

For gels, wound dressings, and microneedles, release modeling is even more complex. Matrix swelling, dissolution, erosion, carrier-in-matrix release, tissue deposition, and changing local fluid conditions can produce multiphase release profiles [12,15,16,18,28,32,33,36]. Empirical models may still be useful for comparing formulations, estimating release half-times, identifying burst release, or supporting quality-by-design studies, but they should not replace mechanistic characterization [7,8,9,13,25,30,44,45,46].

### 10.9. Connecting Release to Biological Response

For OA polymeric nanocarriers, the most important release question is not simply how much drug leaves the carrier, but whether the released or carrier-associated OA reaches the relevant biological target [1,5,7,9]. OA may act after extracellular release, membrane partitioning, carrier uptake, intracellular release, or local tissue retention. Different carrier designs may therefore produce different exposure pathways [8,12,14,15,16,17,18,32].

In cancer-oriented polymeric nanoparticles, the relevant exposure may involve cellular uptake and intracellular availability [7,9]. In psoriasis-oriented hyaluronic-acid systems, receptor-associated uptake and reduction-responsive release may be central [12]. In topical gels, skin deposition and local anti-inflammatory effects may matter more than systemic absorption [15]. In osteoarthritis depots, joint residence and sustained intra-articular exposure are likely to be key [18]. In wound dressings, local cytokine response, migration, oxidative stress, and tissue repair endpoints may be more informative than cumulative release alone [8,35,36,37,38].

This means that release studies should be paired with biological and analytical exposure measurements. For topical systems, skin deposition and local biomarker changes may be essential. For intra-articular systems, tissue distribution, synovial residence, cartilage protection, and inflammatory markers may be relevant. For wound dressings, migration, cytokines, oxidative-stress markers, and wound closure should be considered [12,15,16,18,35,36,37].

A slower release profile is not automatically better. If release is too slow, OA may remain trapped and biologically inactive. If release is too fast, sustained exposure may be lost. The optimal release profile must be defined by therapeutic objective, tissue environment, dose, safety, and required duration of action [1,5,7,8,9,12,13,14,15,16,17,18].

### 10.10. Release-Design Implications

Controlled release is the mechanistic bridge between polymeric carrier design and biological performance in OA delivery. Because OA is poorly soluble, lipophilic, and formulation-dependent, release cannot be interpreted only as cumulative percentage released into buffer [1,5,7,8,9]. It must be understood through polymer composition, carrier architecture, OA physical state, release medium, tissue deposition, and biological response [7,8,9,12,13,14,15,16,17,18,33].

Polymeric OA systems can produce release through diffusion, swelling, degradation, erosion, micelle dissociation, gel relaxation, prodrug cleavage, responsive bond cleavage, carrier-in-matrix behavior, or matrix-assisted local retention. These mechanisms are not interchangeable. Their relevance depends on whether the intended application is oral delivery, cancer-oriented delivery, psoriasis, wound healing, osteoarthritis, dermal delivery, or microneedle-assisted administration [7,8,9,12,13,14,15,16,17,18,32,33].

For OA, a controlled-release system should be judged by functional exposure rather than by release duration alone. The best design is not necessarily the slowest or the most complex; it is the one that produces reproducible OA availability at the intended biological site for the intended period of action.

## 11. Translational and Clinical Perspectives

### 11.1. Translational Framing of OA Polymeric Nanocarriers and Polymer-Assisted Delivery Platforms

The translational potential of polymeric nanocarriers and polymer-assisted delivery platforms for oleanolic acid should be discussed with caution. Most available evidence remains preclinical and is based on formulation studies, cell models, or animal experiments [1,5,7,9]. OA has broad reported biological activities, but pharmacological breadth alone does not create a clinical product. The compound must be delivered reproducibly, safely, and at concentrations that are meaningful for the intended tissue and disease context.

The main translational limitation of OA is not only pharmacodynamics. It is exposure. Poor aqueous solubility, low and variable bioavailability, crystallinity, limited barrier transport, and vehicle-dependent activity make it difficult to compare studies and to define realistic dosing strategies [1,5]. Polymeric nanocarriers are relevant because they may convert OA from a poorly dispersed hydrophobic compound into a formulation with more controllable release, retention, and tissue exposure [5,7,8,9,12,14,16,18,32,36].

A polymeric OA system should not be considered clinically promising only because it increases apparent solubility or improves activity in vitro [7,8,9,12,14,16,18,32]. Clinical relevance requires a connection between carrier design, release kinetics, tissue exposure, safety, disease biology, patient need, and manufacturability. This is why translational assessment should focus on specific use cases rather than on general enhancement of OA delivery.

The most realistic near-term directions are local and tissue-targeted applications. These include inflammatory skin disease, psoriasis, wound healing, dermal delivery, osteoarthritis, and possibly other localized inflammatory conditions [8,12,14,16,18,32]. In these settings, sustained exposure at the target site may be more important than high systemic bioavailability. Systemic applications such as cancer, liver injury, metabolic disease, and oral bioavailability enhancement remain scientifically relevant, but they require stronger pharmacokinetic, biodistribution, safety, and comparator data before clinical value can be claimed [5,7,9,38,39,43].

### 11.2. Unmet Medical Needs and Formulation Relevance

Several disease areas associated with OA biology involve chronic inflammation, oxidative stress, tissue remodeling, barrier dysfunction, cartilage degeneration, or abnormal cell proliferation [1,5]. These processes are relevant to inflammatory skin disease, wound repair, osteoarthritis, liver injury, metabolic disorders, and cancer [26,38,39,43]. However, OA’s poor delivery properties limit direct therapeutic use and provide a rationale for formulation-based intervention [1,5].

Polymeric nanocarriers may address these needs by enabling controlled release, sustained local residence, improved tissue deposition, reduced dosing frequency, and better handling of poorly soluble OA. These advantages are most relevant when conventional free-drug administration cannot maintain sufficient exposure or when repeated administration is impractical [5,7,8,9,12,14,16,18,32]. In this sense, polymeric delivery is not merely a technical enhancement. It may make OA biologically testable in settings where unformulated OA is difficult to interpret.

The unmet-need argument must be disease-specific. Psoriasis requires local anti-inflammatory and immunomodulatory effects in skin. Wound healing requires stage-dependent modulation of inflammation, migration, oxidative stress, extracellular matrix deposition, and tissue repair. Osteoarthritis requires joint residence and cartilage- or synovium-relevant activity. Cancer requires selective tumor exposure, penetration, and safety. Liver and metabolic applications require systemic exposure, target-tissue distribution, and long-term tolerability [8,12,14,16,18,26,32,38,39,43]. A single OA formulation is unlikely to be optimal for all these indications.

Future development should therefore avoid the idea of a generally “best” OA nanocarrier. Instead, polymeric systems should be matched to disease biology, administration route, required release duration, safety constraints, and realistic clinical comparators [5,7,8,9,12,14,16,18,32].

### 11.3. Inflammatory Skin Diseases and Psoriasis

Inflammatory skin disease is one of the strongest translational directions for polymeric OA delivery. OA has reported anti-inflammatory activity, and local delivery may provide therapeutic exposure in skin while limiting systemic burden [1,5,12]. This route also matches OA’s formulation limitations: poor solubility and restricted passive penetration can be addressed by polymeric micelles, hyaluronic-acid-based nanoparticles, hydrogels, nanogels, and microneedle-compatible systems [12,14,16,32].

The hyaluronic-acid-based reduction-responsive OA nanoparticle system developed for psoriasis is particularly important because it links polymer chemistry with disease-oriented delivery [12]. The system combines OA–hyaluronic acid conjugation, disulfide-responsive behavior, topical application, and CD44-related cellular uptake in keratinocytes [12]. This makes it more translationally informative than a formulation designed only to increase apparent solubility.

For psoriasis and inflammatory skin disease, the main development questions are local deposition, repeated-dose safety, irritation, barrier recovery, disease-relevant activity, and comparison with current topical therapies [12,14,16,32]. A formulation that improves OA uptake in cells must still show that it can deliver useful exposure in diseased skin without unacceptable irritation or barrier disruption.

Microneedle-assisted systems may become relevant in this area, especially for localized plaques or difficult-to-penetrate lesions [12,16,32,33,36]. However, OA-loaded microneedles should be presented as a future-oriented platform rather than an established OA formulation class. Their value will depend on whether they improve skin deposition, dose reproducibility, patient acceptability, and safety compared with simpler topical gels, micelles, or HA-based nanoparticles [12,14,16,32,33].

### 11.4. Wound Healing and Tissue Repair

Wound healing is another plausible local application for polymeric OA delivery. OA has been associated with wound-healing-related activity, cell migration, anti-inflammatory effects, and oxidative-stress modulation [1,5,37,38]. However, wound healing is a dynamic process, and the usefulness of OA will depend on timing, dose, tissue context, and formulation design.

Polymeric wound dressings, hydrogels, nanocarrier-loaded dressings, and PLGA fiber membranes are relevant because they can combine OA delivery with tissue contact, moisture control, mechanical protection, and local retention [35,36,37,38]. In these systems, the polymeric matrix is not only a carrier. It also defines how the formulation interacts with wound exudate, damaged tissue, inflammatory cells, and the local repair environment.

The translational challenge is that wound biology is stage-dependent. A formulation may need to reduce excessive inflammation without suppressing necessary immune responses, support migration without causing abnormal proliferation, and maintain moisture without increasing infection risk. OA release data alone are therefore insufficient. Evaluation should include keratinocyte and fibroblast behavior, inflammatory cytokines, oxidative-stress markers, collagen deposition, wound closure, infection risk, exudate interaction, and tissue compatibility [35,36,37,38].

For clinical development, OA wound systems should be compared not only with free OA but also with relevant wound-care materials or advanced dressings. If a nanocarrier-loaded dressing is more complex than a conventional dressing, it must justify that complexity by better tissue response, dosing convenience, safety, or healing performance [8,35,36,37,38].

### 11.5. Osteoarthritis and Intra-Articular Local Therapy

Osteoarthritis is a strong example of a localized disease where systemic bioavailability may not be the most important formulation endpoint. The disease involves cartilage degeneration, synovial inflammation, oxidative stress, pain, and mechanical joint dysfunction within an accessible anatomical compartment [18]. Intra-articular delivery can provide high local exposure, but rapid clearance from the joint limits the usefulness of simple formulations.

The OA cubic liquid crystal nanoparticle-based thermosensitive gel developed for knee osteoarthritis in rats is therefore one of the most clinically oriented OA delivery examples [18]. It combines OA-loaded nanostructures with a Poloxamer thermosensitive gel base, creating a local depot designed to improve intra-articular residence and sustained exposure [18]. This approach is valuable because it is linked to a specific disease, route, and anatomical site.

For osteoarthritis, the formulation must be assessed through joint-relevant criteria: injectability, gelation temperature, viscosity, residence time, release in synovial-like conditions, sterility, endotoxin control, cartilage compatibility, synovial irritation, and repeated-dose safety [18]. These requirements are more demanding than simple in vitro release or cell-viability assays.

The comparator question is also important. Future OA intra-articular systems should be compared with clinically relevant standards such as hyaluronic acid injections, corticosteroids, nonsteroidal anti-inflammatory approaches, or other local therapies where appropriate [18]. Without such comparisons, it is difficult to judge whether OA depots offer practical value beyond formulation novelty.

### 11.6. Cancer Nanomedicine

Cancer is relevant to OA polymeric delivery because OA has preclinical anticancer activity and polymeric carriers can alter cellular exposure, uptake, and cytotoxic response [7,9,38,43]. OA-loaded PEGylated PLA/PLGA nanoparticles and OA/ursolic-acid-loaded PLGA nanoparticles provide direct examples of polymeric carrier systems evaluated in cancer-cell models [7,9].

However, cancer nanomedicine requires cautious interpretation. Increased cytotoxicity in monolayer cell culture does not establish clinical anticancer potential [7,9]. A polymeric carrier may increase apparent activity by improving dispersion, reducing precipitation, altering uptake, or changing intracellular availability rather than by enhancing intrinsic OA potency [5,7,9].

For cancer applications, the key development questions include tumor penetration, uptake mechanism, release at the target site, pharmacokinetics, biodistribution, off-target toxicity, immune interaction, and comparison with established anticancer therapies [7,9,43].

Three-dimensional tumor models, spheroids, organoids, and animal biodistribution studies would provide more relevant information than cytotoxicity alone.

Cancer therefore remains scientifically important but translationally difficult. Among the directions discussed in this review, it likely faces a longer development path than local dermatological, wound, or intra-articular applications because systemic exposure, safety margins, and clinical comparators are more demanding [7,9,18,38,43].

### 11.7. Liver Injury, Metabolic Disorders, and Systemic Applications

OA has a long history of investigation in hepatoprotection, metabolic regulation, antioxidant signaling, and inflammatory pathways [38,39]. These activities are scientifically important, but systemic clinical development is complicated by poor oral bioavailability, variable exposure, and the need to deliver sufficient drug to target tissues without off-target toxicity [1,5].

Polymeric nanocarriers may improve systemic OA delivery by increasing apparent dispersion, protecting the compound, modifying pharmacokinetics, and supporting tissue distribution. However, systemic formulations require more demanding evidence than local formulations because they must demonstrate absorption, circulation stability, biodistribution, metabolism, clearance, and safety [5,7,9,25].

For liver-related applications, carrier design should specify the intended target: hepatocytes, Kupffer cells, hepatic stellate cells, inflammatory pathways, antioxidant response, or metabolic regulation. A formulation that improves plasma exposure may not necessarily improve therapeutic exposure in the relevant liver cell population. Liver-targeted OA polymeric systems therefore require cell-type-specific and pharmacokinetic evaluation [5,25,38,39].

For metabolic disorders, oral or injectable OA systems would need to show sustained exposure, acceptable long-term safety, and disease-relevant outcomes [1,5]. Because metabolic indications usually require chronic administration, polymer safety, degradation products, excipient burden, cost, and patient adherence become central considerations [47]. These requirements make local indications more realistic as initial translational targets [8,12,14,18].

Overall, liver and metabolic applications remain biologically plausible but developmentally demanding. Polymeric carriers may help address exposure limitations, but stronger pharmacokinetic and disease-model evidence is needed before clinical relevance can be claimed [1,5,8,9,12,14,18,25,38,39].

### 11.8. Oral Delivery and Bioavailability Improvement

Oral delivery is attractive because of patient convenience, but it is one of the most difficult routes for OA because of poor aqueous solubility and low bioavailability [1,5]. Many approaches have been used to improve oral exposure, including solid dispersions, cyclodextrin complexes, phospholipid complexes, self-emulsifying systems, lipid nanoparticles, and polymeric carriers [5,17,19,20].

Polymeric nanocarriers could improve oral OA delivery by enhancing dispersion, protecting the drug in gastrointestinal conditions, modulating release, interacting with mucus, or promoting uptake through intestinal pathways. However, oral nanocarrier translation is difficult. Gastrointestinal dilution, enzymes, bile salts, mucus, food effects, pH variation, transit time, and interindividual variability can all influence performance [5,17,19,20,25].

For OA, the key oral-delivery question is comparative. A polymeric nanocarrier must show an advantage over simpler and often more scalable systems such as self-emulsifying formulations, solid dispersions, or cyclodextrin complexes [5,17,19,20]. If a polymeric system improves in vitro solubility but does not improve in vivo exposure, tissue availability, safety, or therapeutic effect, its translational value may be limited.

Future oral OA studies should include pharmacokinetic evaluation, food-effect considerations, gastrointestinal stability, mucus interaction, release under biorelevant conditions, and comparison with established solubilization systems [5,17,19,20,25]. Oral delivery remains important, but it should be approached as a high-barrier route rather than an automatic consequence of nanosizing.

**Figure 6:**
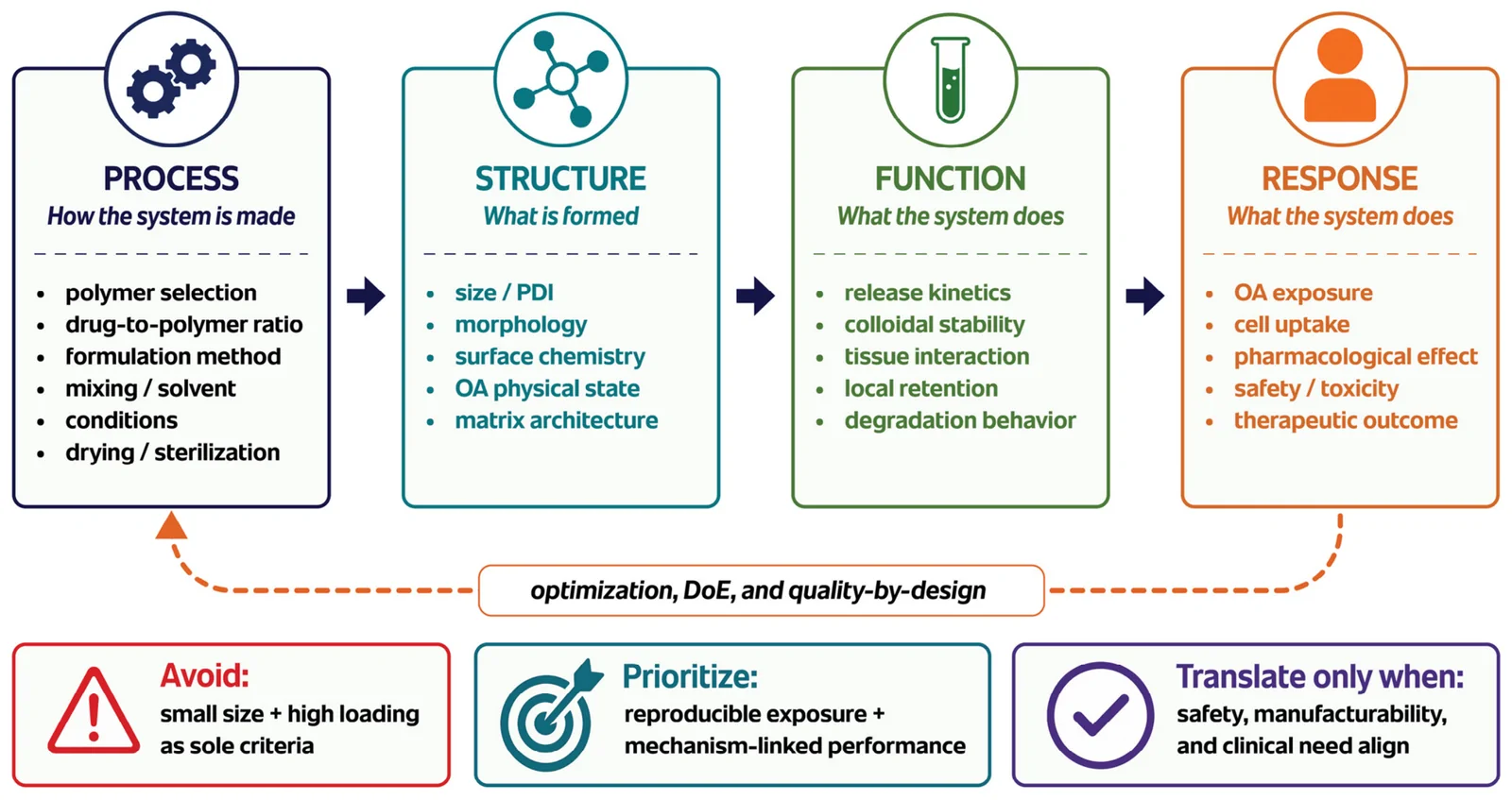
Process–structure–function–response framework for OA-loaded polymeric nanocarriers and polymer-assisted delivery platforms. The figure illustrates how formulation performance depends on the relationship between process parameters, carrier structure, functional behavior, and biological response. Polymer selection, drug-to-polymer ratio, solvent conditions, drying, and sterilization influence particle or matrix architecture, OA physical state, release kinetics, colloidal stability, tissue interaction, safety, and therapeutic outcome. The framework emphasizes that small size and high loading should not be used as the only development criteria.

The process–structure–function–response logic proposed for OA polymeric delivery is summarized in Figure 6.

### 11.9. Patient-Centered and Product-Development Considerations

Clinical usefulness depends not only on biological activity but also on patient-centered and product-development factors. Topical gels, patches, wound dressings, intra-articular depots, oral formulations, and microneedle systems differ greatly in ease of use, dosing frequency, storage needs, cost, comfort, and acceptability [15,16,30,35]. These factors can determine whether a formulation is realistic even when the biological rationale is strong.

For chronic skin disease, a patient-friendly OA formulation would need to be non-irritating, cosmetically acceptable, stable, easy to apply, and compatible with repeated use [12,14,16,30]. For wound healing, the product would need to fit into dressing changes, moisture management, infection control, and wound-care practice [8,35,36]. For osteoarthritis, injectability, dosing interval, pain during administration, and compatibility with existing intra-articular therapies would be important [15,18].

Polymeric nanocarriers add value only when the added complexity is justified by better performance. If a simple gel, micelle, or cyclodextrin complex provides sufficient local exposure, a complex multi-component nanocarrier may not be necessary [12,14,16,19]. Conversely, if sustained release, targeting, tissue retention, or barrier bypass is required, a polymeric or polymer-assisted system may be justified [7,8,9,14,16,18,30,32].

Cost and scalability also matter. Biodegradable polymeric nanoparticles, microneedles, nanogels, and polymer–drug conjugates may require specialized manufacturing, sterilization, packaging, quality control, and stability programs [16,26,32,43]. Translational development should therefore balance formulation sophistication with manufacturability, regulatory feasibility, patient need, and expected clinical advantage.

### 11.10. Regulatory and Safety Perspective

Regulatory translation of OA polymeric nanocarriers would require clear definition of the active compound, excipients, polymeric carrier, manufacturing process, critical quality attributes, release profile, safety, and intended clinical use [5,7,8,9,25,43].

Natural origin does not reduce the need for rigorous safety evaluation. OA exposure, impurities, excipients, polymer degradation products, and formulation components can all affect toxicity [1,5,25,43].

For polymeric nanoparticles, safety evaluation should include polymer degradation products, residual solvents, stabilizer or surfactant toxicity, complement activation where relevant, hemocompatibility for injectable systems, off-target tissue distribution, and immune interaction [7,8,9,25]. For topical systems, irritation, sensitization, barrier disruption, photostability if relevant, and repeated-dose local tolerability are critical [12,14,16,30,43]. For intra-articular systems, synovial irritation, cartilage compatibility, sterility, endotoxin burden, viscosity, and repeated injection safety are essential [15,18].

Combination platforms such as microneedles and drug-loaded wound dressings may face additional regulatory complexity because they combine drug delivery with device-like or biomaterial functions [32,36,40]. Dose uniformity, mechanical performance, microbial control, sterility, packaging stability, patient use, and application reproducibility should be considered early in development rather than after biological proof of concept [16,30,35].

For OA specifically, the field would benefit from standardized reporting of dose, formulation composition, free versus carrier-associated drug, release conditions, biological controls, and exposure verification [5,7,8,9]. Without such reporting, comparison between studies will remain difficult and claims of translational potential will remain weak.

### 11.11. Translational Readiness Across Application Areas

Based on current evidence, local dermatological delivery appears to be one of the most promising directions for OA polymeric systems. This area has direct support from hyaluronic-acid-based OA nanoparticles for psoriasis, polymeric OA micelles for skin application, and broader polymeric or microneedle literature in skin delivery [14,16,30,32]. The main remaining challenges are repeated-dose safety, irritation, skin deposition, barrier recovery, comparison with existing topical therapies, and scalable formulation.

Wound healing is also plausible, especially for hydrogels, PLGA fiber membranes, and nanocarrier-loaded dressings. The main barriers are stage-specific wound biology, infection risk, wound-exudate effects, realistic wound models, and comparison with advanced dressings or standard wound care [8,35,36,37,38].

Osteoarthritis has a strong local-depot rationale because intra-articular delivery can theoretically maintain OA near the diseased joint [18]. The thermosensitive nanogel study provides disease-oriented support, but translation would require stronger evidence on joint residence, cartilage compatibility, repeated dosing, synovial safety, mechanical joint conditions, and comparison with existing intra-articular therapies [15,18].

Cancer, liver injury, metabolic disease, and oral bioavailability enhancement remain important but less immediately translational. These indications require stronger pharmacokinetic, biodistribution, target-tissue exposure, long-term safety, and comparator evidence [5,7,9,19,20,38,39,43,47]. They should be presented as longer-term directions unless direct OA polymeric systems provide more advanced in vivo evidence.

A complementary application-oriented summary of suitable polymeric systems and main translational barriers is provided in Table S2.

### 11.12. Clinical Positioning

The clinical future of OA polymeric nanocarriers and polymer-assisted delivery platforms will depend on matching formulation design to a realistic use case. Local and tissue-targeted systems appear most promising in the near term because they can use controlled release and tissue residence to compensate for OA’s poor systemic bioavailability [8,14,16,18,32]. Psoriasis, inflammatory skin disease, wound healing, dermal delivery, and osteoarthritis are therefore stronger early translational targets than broad systemic applications.

Systemic and oral applications remain scientifically relevant but require more demanding evidence. For cancer, liver injury, metabolic disease, and oral bioavailability enhancement, polymeric systems must demonstrate not only improved formulation properties but also pharmacokinetic advantage, target-tissue exposure, safety, and superiority over simpler or established delivery approaches [5,7,9,19,20,38,39,43,47].

For OA, translational progress should be judged by functional necessity. A polymeric nanocarrier is justified when it solves a defined clinical delivery problem: local retention, barrier bypass, controlled release, responsive tissue exposure, dose reduction, or improved safety. Formulation complexity should advance only when it improves performance compared with simpler alternatives.

## 12. Manufacturing, Regulatory, and Scale-Up Challenges

### 12.1. Why Manufacturability Matters for OA Polymeric Nanocarriers

Manufacturability is a central issue for OA-loaded polymeric and polymer-assisted nanocarriers because formulation performance depends strongly on material attributes, process conditions, and batch reproducibility [7,9,25,43,47]. A system that performs well as a small laboratory batch may not remain stable, reproducible, sterile, or clinically useful after scale-up. This is particularly important for OA because poor solubility, crystallinity, hydrophobicity, and vehicle-dependent activity make the compound sensitive to solvent history, mixing, drying, polymer composition, and storage conditions [1,5,19,20,48].

Many OA formulations are reported as proof-of-concept systems, where the main objective is to demonstrate drug loading, improved dispersion, sustained release, or enhanced biological response [7,9,12,14,15,18]. Translational development requires a different standard. The formulation must be prepared reproducibly, characterized consistently, stored reliably, and manufactured through processes compatible with quality control, sterility or microbial limits where needed, and regulatory expectations [25,43,47].

For polymeric OA systems, manufacturability includes polymer sourcing, raw OA quality, solvent control, process parameters, nanoparticle or matrix formation, drug loading reproducibility, residual solvent removal, drying or lyophilization, sterilization or microbial control, packaging, and stability [7,9,13,25,43,47]. Each of these factors can affect particle size, OA physical state, release profile, biological exposure, and safety. Manufacturing should therefore be considered part of formulation design, not a final technical step.

### 12.2. Critical Material Attributes

Critical material attributes are especially important for OA polymeric systems because both the drug and the carrier materials influence final performance [1,5,7,19,20,47]. For PLA and PLGA systems, relevant attributes include polymer molecular weight, lactide ratio, end-group chemistry, intrinsic viscosity, crystallinity, residual monomers, water content, degradation behavior, and batch-to-batch variability [13,25,43,47].

OA itself should also be treated as a critical material input rather than a generic active ingredient. Purity, polymorphic form, particle size, residual solvents, impurity profile, botanical or synthetic origin, and storage conditions may influence loading, crystallization, release, and biological response [1,5,19,20,47,48]. For a poorly soluble compound, differences in raw material quality can translate directly into formulation variability.

Excipients such as PEGylated polymers, hyaluronic acid, poloxamers, cyclodextrins, chitosan, surfactants, stabilizers, hydrogel-forming polymers, and microneedle-forming polymers also require control [12,14,15,16,18,29,32,33]. Their molecular weight, substitution degree, charge, purity, moisture content, degradation profile, and biological interaction may influence carrier stability and tissue response. In advanced polymer-assisted systems, an excipient may be part of the therapeutic mechanism rather than a passive ingredient.

Material specifications should therefore be linked to critical quality attributes. Polymer molecular weight may affect particle size and release; hyaluronic-acid molecular weight or substitution degree may affect cellular interaction and degradation; poloxamer concentration may affect gelation temperature and local retention; and cyclodextrin substitution may affect OA complexation and release [12,15,18,31]. This connection between material attributes and formulation performance is essential for quality-by-design-oriented development.

### 12.3. Critical Process Parameters

Critical process parameters are the manufacturing variables that determine whether the intended formulation can be reproduced. For OA-loaded nanoparticles, these may include solvent type, solvent-to-water ratio, polymer concentration, OA concentration, stabilizer concentration, mixing rate, injection mode, temperature, purification method, and solvent-removal procedure [7,9,13,25].

For hydrogels, thermosensitive depots, and nanocarrier-loaded matrices, process parameters include polymer concentration, mixing order, temperature, hydration time, pH, ionic strength, gelation conditions, carrier distribution, and filling or casting procedure [15,18,25]. For electrospun fiber membranes, polymer solution viscosity, solvent composition, voltage, flow rate, collector distance, humidity, and drying conditions can affect fiber diameter, morphology, drug distribution, and release [8]. For microneedles, molding, casting, drying, backing-layer formation, humidity, and demolding can affect geometry, mechanical strength, dose uniformity, and carrier stability [16,32,33].

OA makes these parameters especially important because uncontrolled supersaturation, solvent evaporation, drying, or concentration changes may promote crystallization or phase separation [1,5,7,8,9]. A process that produces high loading in one batch may produce different OA physical state or release behavior in another batch if mixing, drying, or purification conditions change.

Process descriptions should therefore be detailed enough to allow reproduction. Reporting only the final particle size and encapsulation efficiency is not sufficient. Studies should describe material sources, concentrations, processing sequence, temperature, solvent-removal conditions, purification, drying, storage, and the number of independent batches evaluated [7,9,13,25,43,47].

### 12.4. Scale-Up of Polymeric Nanoparticles

Scale-up of polymeric nanoparticles is difficult because the processes used at laboratory scale may not translate directly to larger batches. Nanoprecipitation, solvent displacement, and emulsion-based methods depend on mixing time, local concentration gradients, solvent diffusion, interfacial behavior, and energy input [7,9,13,25]. These parameters can shift during scale-up and change particle size, polydispersity, OA loading, and release.

Batch nanoprecipitation may be simple for screening, but it can be sensitive to injection speed, vessel geometry, stirring rate, solvent ratio, and operator technique [7,9,13]. Emulsion-based methods may face additional challenges related to droplet-size control, surfactant removal, residual solvent, and scale-dependent shear [7,9,13,25]. For OA, both approaches must also control drug precipitation and physical state.

Continuous or semi-continuous methods, including microfluidic mixing, controlled impingement, or scalable precipitation platforms, may improve process control for polymeric nanoparticles [43,49,50]. These approaches can provide better control over mixing and nucleation, but they require optimization of flow rate, concentration, solvent compatibility, throughput, fouling, and downstream processing [49,50]. Their value for OA formulations would need to be demonstrated through reproducible loading, physical-state control, release, and stability.

Scale-up should be evaluated through multiple independent batches rather than by increasing batch size once. Important endpoints include particle size, PDI, zeta potential, OA loading, encapsulation efficiency, residual solvent, physical state, release profile, microbial quality, stability, and biological performance [7,9,13,25,43,47]. Without such data, claims of translational potential remain premature.

### 12.5. Manufacturing of Hydrogels, Depots, Dressings, and Microneedles

Manufacturing challenges differ by dosage form. Hydrogels and thermosensitive depots require control of polymer concentration, mixing order, viscosity, gelation temperature, injectability or spreadability, drug distribution, microbial quality, and filling conditions [15,18,28]. For OA-loaded systems, the distribution of the embedded nanocarrier or drug phase within the gel is especially important because nonuniformity can lead to dose variability and inconsistent release.

Wound dressings and fiber membranes require control of matrix morphology, mechanical strength, porosity, thickness, residual solvent, drug distribution, sterilization compatibility, packaging, and moisture behavior [8,35,36]. If OA is incorporated through nanoparticles, micelles, or cyclodextrin complexes, the stability of those carriers after embedding and drying must also be verified [8,14,15,29].

Microneedles introduce device-like manufacturing requirements. Needle height, base width, tip sharpness, array density, mechanical strength, insertion efficiency, dose uniformity, dissolution or swelling time, residual moisture, and packaging stability must be controlled [16,32,33]. If OA-loaded nanocarriers are embedded in microneedles, the carrier should be characterized before fabrication, after drying, after storage, and after dissolution or swelling [7,8,9,18,25,26,27,31].

These dosage forms are often more clinically practical than nanoparticle suspensions alone, but they are also more complex as products. A nanocarrier-loaded hydrogel, dressing, depot, or microneedle patch must be controlled as the complete formulation, not only as the nanoscale component.

### 12.6. Sterilization and Microbial Control

Sterilization or microbial control is a major issue for OA polymeric systems intended for topical, wound, intra-articular, transdermal, or injectable use. The required level of control depends on route and product type. Intra-articular depots and injectable systems require strict sterility and endotoxin control, whereas topical gels and wound dressings require microbial limits or sterility depending on intended use and regulatory classification [15,18,35,36].

Common sterilization methods may affect polymeric nanocarriers. Heat can accelerate polymer degradation, alter gelation, or promote OA crystallization. Gamma irradiation or electron-beam sterilization may affect polymer chains, drug stability, or mechanical properties. Filtration may not be feasible for gels, fibers, microneedles, or larger nanocarrier systems. Aseptic manufacturing may be required for sensitive formulations [15,18,25,35,36].

For OA, sterilization should be evaluated not only by microbial outcome but also by formulation performance. Size, PDI, OA loading, physical state, release profile, rheology, mechanical behavior, dose uniformity, and biological activity may change after sterilization [7,9,15,16,18]. This is especially important for thermosensitive gels, wound dressings, and microneedle patches, where polymer structure and mechanical behavior are part of the product function.

### 12.7. Drying, Lyophilization, and Storage

Drying and storage are critical for OA-loaded polymeric systems because water removal, humidity, temperature, and packaging can alter carrier structure and drug physical state [1,5,7,8,9]. Lyophilization may improve shelf-life for nanoparticle dispersions, but it can also cause aggregation, changes in release, or drug crystallization unless cryoprotectants and reconstitution conditions are optimized [7,9,13].

For microneedles, fiber membranes, and wound dressings, drying is part of the manufacturing process rather than a post-processing option [8,16,32,33]. Drying conditions can influence residual moisture, mechanical strength, OA distribution, carrier stability, and crystallization risk. For hydrogels and thermosensitive depots, storage may involve hydrated or semisolid states, which introduce risks of phase separation, microbial growth, syneresis, viscosity change, and drug redistribution [15,18].

Stability studies should monitor more than appearance. Key parameters include OA content, degradation products, physical state, particle size, release profile, residual moisture, rheology, mechanical strength, dose uniformity, sterility or microbial quality, and biological activity where relevant [7,8,9,16,18,32,33]. Packaging should protect against moisture, oxygen, light, microbial contamination, and mechanical damage according to the dosage form.

### 12.8. Reproducibility and Batch-to-Batch Variability

Batch-to-batch reproducibility is essential for OA polymeric nanocarriers because small process variations may change particle size, OA physical state, loading, release, and biological response [1,5,7,9,20,39,43,47,48]. This is particularly relevant for poorly soluble compounds whose activity may depend strongly on vehicle and exposure conditions [1,5,20,48].

Preclinical studies often report results from optimized batches, but translational development requires multiple independently prepared batches tested against the same critical quality attributes [7,9,39,43,47]. These attributes should include not only size and encapsulation efficiency, but also physical state, release, stability, residual solvent, and biological activity where relevant [1,5,20,27,43].

Reproducibility becomes more difficult for multi-component systems. Hyaluronic-acid nanoprodrugs, nanocarrier-loaded hydrogels, thermosensitive depots, fiber membranes, and microneedle patches involve several material and process steps, each of which may introduce variability [8,12,15,16,18,32,33]. Batch control must therefore cover the full product rather than only the nanoparticle or active-drug component.

A useful approach is to define acceptable ranges for critical quality attributes and link them to biological performance. A range of particle sizes may be acceptable if release and biological activity remain consistent, while a small change in OA physical state may be unacceptable if it alters release or tissue exposure [1,5,7,9,20,25,27,31,39,43,47,48]. This performance-based logic is central to QbD development.

### 12.9. Quality-by-Design and Risk-Based Development

Quality-by-design provides a useful framework for OA polymeric nanocarrier development because it connects product design, process understanding, risk assessment, and quality control [7,14,20,39,43,47]. Instead of relying only on empirical optimization, QbD encourages definition of the quality target product profile, critical material attributes, critical process parameters, and critical quality attributes.

For OA systems, the target product profile should be indication-specific. A topical psoriasis nanoparticle, a wound dressing, an intra-articular thermosensitive gel, an oral polymeric nanoparticle, and a microneedle patch have different dose, release, safety, sterility, stability, and usability requirements [8,12,14,15,16,18,32,33]. The desired product profile should therefore be defined before selecting polymers, carriers, or manufacturing methods.

Risk assessment can help identify variables most likely to affect performance. For OA, high-risk factors may include drug crystallization, incomplete encapsulation, aggregation, solvent residue, poor release reproducibility, polymer degradation, gelation failure, microneedle fracture, skin irritation, joint irritation, and lack of exposure verification [1,5,7,9,16,18,19,20,38,39,43,47,48].

Design-of-experiments approaches may be especially valuable. DoE can evaluate how polymer concentration, drug-to-polymer ratio, solvent ratio, stabilizer amount, mixing conditions, drying, storage, and sterilization affect size, loading, physical state, release, and stability [39,43,47]. This would help move the field from one-off formulation reports toward more systematic design rules.

### 12.10. Regulatory Considerations

Regulatory expectations for OA polymeric and polymer-assisted systems would depend on route, composition, dosage form, and intended indication. A topical gel, oral nanoparticle, intra-articular depot, wound dressing, and microneedle patch may fall under different regulatory categories or require different evidence packages [16,25,30,35,43]. Regulatory considerations should therefore be integrated early into formulation design.

Although OA is a natural compound, natural origin does not reduce the need for rigorous quality and safety evaluation [1,5,25,43]. The active compound, excipients, polymeric carrier, manufacturing process, release profile, impurities, degradation products, and intended use all contribute to risk. For polymeric and hybrid systems, the full formulation must be evaluated, not only OA.

For polymer-assisted lipid or hybrid carriers, regulatory principles from complex nanomedicines, liposomal products, and pharmaceutical development provide useful analogies [25,43]. These emphasize composition control, structure, drug loading, release, stability, pharmacokinetics or local exposure, safety, and reproducible manufacturing. OA hybrid systems can draw from these principles even when they are not strictly liposomal products.

Microneedles, wound dressings, and intra-articular depots may require additional consideration because they combine drug delivery with device-like or biomaterial functions [16,30,35]. Dose uniformity, mechanical performance, sterility, biocompatibility, local tolerability, user handling, and packaging stability may be as important as release kinetics. This reinforces the need to design OA polymeric systems as products rather than only experimental formulations.

### 12.11. Clinical Manufacturing and GMP Considerations

Clinical translation requires manufacturing under controlled quality systems, with defined raw materials, validated processes, validated analytical methods, and documentation [25,39,43,47]. For OA polymeric nanocarriers, GMP-compatible development would require specifications for OA, polymers, excipients, solvents, process parameters, intermediate controls, and final product attributes.

Analytical method validation is particularly important. Methods for OA assay, impurity profiling, residual solvent analysis, release testing, particle size, morphology, physical state, sterility, endotoxin, and stability must be appropriate for the formulation type [7,8,9,12,14,15,16,18,25]. For multi-component systems, separate methods may be needed for the drug, polymer, nanocarrier, gel matrix, dressing, or device component.

Scale-up should also consider equipment compatibility and process robustness. Methods that depend on manual pipetting, small-batch sonication, laboratory-specific drying, or uncontrolled mixing may be difficult to translate. Continuous manufacturing, controlled mixing, spray drying, lyophilization, scalable molding, or aseptic filling may be needed depending on dosage form [16,25,43,49,50,51].

A clinically manufacturable OA product should balance complexity with therapeutic value. If a simple gel, micelle, or cyclodextrin complex provides sufficient local exposure, a highly complex multi-component nanocarrier may not be justified [12,14,17,19]. Conversely, if controlled release, barrier bypass, local retention, or route-specific targeting is necessary, a polymeric or polymer-assisted system may be worth the additional development burden [7,8,9,12,14,15,16,18,32].

### 12.12. Relative Industrial Readiness and Early Development Priorities

None of the OA polymeric platforms discussed in this review can currently be considered industrially mature on the basis of OA-specific evidence alone. Their relative development potential can nevertheless be compared according to material complexity, number of manufacturing steps, process controllability, sterilization requirements, analytical burden, stability, and the availability of pharmaceutical-development precedents. This comparison reflects relative development burden rather than a formal technology-readiness classification.

Conventional PLA/PLGA nanoparticles have a comparatively strong development rationale because the polymers are well established in drug-delivery research and the particles can be produced by nanoprecipitation, solvent displacement, or emulsion-based methods [7,9,13,25,43]. Their main industrial risks are scale-dependent mixing, residual solvent, aggregation, OA crystallization, sterilization, drying or lyophilization, and reproducibility of loading and release. Continuous or controlled-mixing processes may reduce operator dependence, but OA-specific process robustness must still be demonstrated across multiple independent batches.

Simple polymeric micelles and topical gel systems may offer a relatively lower manufacturing burden when they use pharmaceutically familiar excipients and do not require sterile production [14,15]. Their development risks include dilution-dependent micelle dissociation, OA precipitation, microbial quality, rheological stability, dose uniformity, and packaging compatibility. For topical products, reproducible skin deposition and local tolerability may be more important than highly complex nanoscale architecture.

Electrospun PLGA fiber membranes, nanocarrier-loaded hydrogels, and thermosensitive intra-articular depots have an intermediate development burden. These platforms provide clear local-delivery functions, but the complete dosage form must be controlled. Fiber membranes require reproducible morphology, thickness, porosity, drug distribution, residual-solvent removal, mechanical properties, sterilization compatibility, and packaging [8]. Nanocarrier-loaded hydrogels and thermosensitive depots require control of both the embedded carrier and the macroscopic matrix, including carrier distribution, viscosity, gelation, injectability, local residence, sterility or microbial quality, and stability [15,18].

Hyaluronic-acid-based nanoprodrugs, multicomponent stimuli-responsive systems, and nanocarrier-loaded microneedles have the highest relative development burden among the platforms considered. HA-based nanoprodrugs require reproducible polymer–drug conjugation, control of substitution degree, free OA, molecular-weight distribution, linker stability, degradation products, and biological interaction [12]. Nanocarrier-loaded microneedles additionally combine nanocarrier manufacturing with molding, drying, mechanical testing, dose-uniformity control, insertion performance, residual-moisture control, and device-like packaging requirements [16,32,33].

Industrial potential should therefore be assessed against the intended clinical benefit. A more complex system is justified only when it provides a measurable advantage that cannot be achieved by a simpler formulation, such as prolonged local residence, barrier bypass, controlled release, disease-oriented interaction, reduced dosing frequency, or improved safety. For local OA applications, a simple gel, micelle, or polymeric matrix may be preferable when it provides sufficient exposure and stability. More complex responsive or device-integrated systems require correspondingly stronger evidence of therapeutic advantage.

Several parameters should be defined from the earliest development stage: the intended indication and route; target dose and dose volume; required exposure duration; acceptable release profile; OA raw-material specifications; polymer or excipient specifications; critical process parameters; residual-solvent limits; physical-state requirements; sterilization or microbial-control strategy; packaging; storage conditions; analytical methods; batch-reproducibility criteria; clinically relevant comparator; and expected manufacturing throughput. Establishing these requirements early can prevent selection of a formulation that performs well at laboratory scale but cannot be manufactured, stored, administered, or controlled as a pharmaceutical product.

For OA, the most industrially promising platform will not necessarily be the one with the highest encapsulation efficiency or the most sophisticated architecture. It will be the platform that combines a defined therapeutic purpose with reproducible preparation, stable storage, scalable processing, route-appropriate quality attributes, and a measurable advantage over simpler alternatives [16,32,33].

## 13. Future Directions and Research Roadmap

### 13.1. From Formulation Feasibility to Clinically Oriented Design

Future research on OA-loaded polymeric nanocarriers and polymer-assisted delivery platforms should move beyond demonstrating that OA can be incorporated into another carrier or matrix [1,5,7,8,9,12]. This feasibility has already been shown for PEGylated PLA/PLGA nanoparticles, PLGA nanoparticles, hyaluronic-acid-based nanoprodrugs, polymeric micelles, PLGA fiber membranes, topical gels, and thermosensitive local depots [7,8,9,12,14,15,16,18]. The next stage is to determine which of these systems can provide reproducible, safe, and route-appropriate OA exposure.

The central shift should be from carrier construction to product logic. Polymer type, carrier architecture, drug loading, release profile, route of administration, and biological model should be selected according to a defined disease problem rather than optimized in isolation. A formulation designed for psoriasis, a wound dressing, an intra-articular depot, an oral delivery system, and a cancer-oriented nanoparticle require different design criteria and different evidence [7,8,9,12,14,15,16,18].

For inflammatory skin disease, the priorities are local deposition, barrier compatibility, controlled release in skin, minimal irritation, and comparison with topical standards [12,14,16]. For wound healing, the formulation must support tissue contact, moisture management, stage-relevant repair biology, and compatibility with wound-care practice [8,35,36,37]. For osteoarthritis, injectability, joint residence, cartilage compatibility, synovial tolerability, and repeated-dose safety are central [15,18]. For cancer, liver, metabolic, or other systemic applications, pharmacokinetics, biodistribution, target-tissue exposure, and safety become much more demanding [8,35,36,37,38].

### 13.2. Disease-Specific Target Product Profiles

A clear target product profile should be defined before selecting the carrier. For OA, this profile should specify the intended indication, route of administration, target tissue, required exposure duration, dose range, release objective, safety constraints, comparator, and acceptable level of formulation complexity [5,7,8,9,12,14,15,16,18].

This approach would reduce overgeneralization. There is no universal “best” OA nanocarrier. A polymeric micelle may be useful for dermal delivery but insufficient for sustained intra-articular exposure. A PLGA nanoparticle may support sustained release but may not provide enough skin deposition without a topical matrix or microneedle platform. A thermosensitive gel may improve joint residence but would be unnecessary for applications where rapid release is preferred [8,12,15,18].

Target product profiles would also help determine whether polymeric complexity is justified. If a simple gel, cyclodextrin complex, or self-emulsifying formulation provides sufficient exposure, a multi-component nanocarrier may not be needed [17,19,20]. Conversely, if the therapeutic goal requires local retention, barrier bypass, prolonged release, responsive delivery, or reduced systemic distribution, polymeric and polymer-assisted systems become more rational [7,8,9,12,15,16,18].

### 13.3. Prioritizing Local and Tissue-Targeted Applications

The strongest near-term opportunities for OA polymeric delivery are likely to be local and tissue-targeted applications. These include psoriasis, inflammatory skin disease, wound healing, dermal delivery, osteoarthritis, and other localized inflammatory disorders [8,12,14,15,16,18,32,36,37,38]. These indications match the strengths of polymeric systems because they may benefit from sustained local exposure, controlled release, tissue retention, and reduced systemic burden.

Local delivery also reduces some of the uncertainty associated with poor systemic bioavailability. For OA, a formulation does not always need to maximize plasma concentration if the intended effect is local. A topical gel, nanocarrier-loaded dressing, microneedle patch, or intra-articular depot can be designed around tissue residence rather than systemic absorption [8,12,14,15,16,32].

This does not mean that local systems are simple. They still require dose uniformity, tissue compatibility, release control, irritation testing, sterility or microbial control where relevant, shelf-life stability, and comparison with existing treatments [12,15,16,18,35]. However, the route and endpoint are often more directly connected than in systemic delivery, making local applications a realistic first translational step.

### 13.4. Better Biological Models and Exposure Verification

A major future priority is the use of biological models that match the intended route and disease. Many OA carrier studies still rely heavily on simplified in vitro assays. These models are useful for early screening, but they cannot fully evaluate skin deposition, wound repair, joint residence, tumor penetration, oral absorption, or systemic biodistribution [7,8,9,12,14,15,16,18].

Skin-oriented systems should be evaluated in keratinocyte, immune-cell, reconstructed skin, ex vivo skin, or disease-relevant inflammatory skin models [12,14,16]. Wound systems should include migration, inflammatory cytokines, oxidative-stress markers, collagen deposition, wound closure, infection-related parameters, and tissue compatibility [8,35,36,37,39]. Intra-articular OA systems should include cartilage, synovium, inflammatory markers, pain-related behavior, repeated dosing, and local toxicity [14,15,16,18]. Cancer-oriented systems require three-dimensional tumor models, penetration, uptake, pharmacokinetics, biodistribution, and safety evaluation [7,8,9].

Exposure verification should become routine. Nominal OA dose is not enough for a formulation-dependent compound [1,5,17]. Future studies should quantify OA in release media, skin layers, wound models, joint compartments, cells, or target tissues where possible. This would help determine whether a polymeric carrier improves actual OA availability or only changes apparent solubility.

### 13.5. Physical-State Control as a Development Priority

Physical-state control should be treated as a major development priority for OA-loaded polymeric systems. OA may be crystalline, semi-crystalline, amorphous, molecularly dispersed, surface-associated, or phase-separated in a carrier [1,5,7,8,9]. These states can strongly affect release, storage stability, precipitation after dilution, and biological response.

Future studies should connect physical-state analysis with release and biological performance. Differential scanning calorimetry, X-ray diffraction, Raman spectroscopy, Fourier-transform infrared spectroscopy, microscopy, and validated extraction-based assays should be used more consistently to determine how OA is distributed within polymeric nanoparticles, micelles, fibers, hydrogels, depots, and microneedles [8,9,13].

This is particularly important for high-loading systems. Increasing OA content may improve dose capacity, but excessive loading may also promote crystallization, burst release, incomplete release, or batch variability [1,5,8,9]. A formulation with moderate loading and reproducible release may be more useful than a high-loading system with poorly controlled drug state.

### 13.6. Standardized and Route-Relevant Release Testing

Release testing remains one of the weakest points in many poorly soluble drug-delivery studies. For OA, release profiles are strongly influenced by sink conditions, medium composition, surfactants, proteins, membranes, agitation, and separation methods [1,5,7,8,9,13]. A release method that is analytically convenient may not represent the intended biological environment.

Future OA studies should justify release methods according to route. Oral systems require biorelevant gastrointestinal media. Topical and microneedle systems require skin-compatible receptor media, ex vivo skin, reconstructed skin, or deposition models. Wound dressings should consider exudate-like media, pH, enzymes, proteins, and salts. Intra-articular depots should use synovial-fluid-like conditions where possible [8,12,14,15,16,18,26,32,35,36,37,38,39].

Mass balance should also be improved. Released OA, carrier-retained OA, precipitated OA, tissue-deposited OA, membrane-associated OA, and apparatus-associated OA should be quantified where possible [1,5,7,8,9]. This is especially important because hydrophobic compounds can partition into plastics, proteins, lipids, membranes, and tissue compartments.

### 13.7. Manufacturing-Aware Innovation

Future OA polymeric systems should be designed with manufacturing in mind from the beginning. Many promising systems fail to progress because they are difficult to reproduce, sterilize, dry, store, scale, or package [25,43,47]. This risk is particularly high for multi-component systems such as polymer–drug conjugates, nanocarrier-loaded hydrogels, thermosensitive depots, fiber membranes, and microneedle patches [8,12,15,16,18,32,33].

Quality-by-design approaches should be used more widely. Critical material attributes, critical process parameters, and critical quality attributes should be defined for each product concept [39,43,47]. For OA, high-risk variables include drug crystallization, polymer molecular weight, solvent history, mixing, drying, residual solvent, release variability, gelation behavior, microneedle mechanics, sterilization effects, and storage stability [1,5,7,8,9,16,18].

Manufacturing-aware innovation does not mean avoiding advanced systems. It means ensuring that each added component has a clear function. PEG should improve stabilization or interface behavior; hyaluronic acid should support biological interaction or responsive delivery; hydrogels should improve residence; microneedles should solve a barrier problem; and thermosensitive gels should improve local depot performance [12,14,15,16,18,32,33]. Complexity is justified only when it improves performance compared with simpler alternatives.

### 13.8. Comparators and Decision Criteria

Future OA nanocarrier studies should include clinically and formulation-relevant comparators. Free OA is useful but not sufficient. Depending on the route and indication, relevant comparators may include cyclodextrin complexes, self-emulsifying systems, simple gels, polymeric micelles, standard wound dressings, approved topical therapies, hyaluronic acid injections, corticosteroids, NSAID-based approaches, or other established local treatments [9,12,14,15,16,17,18,19,20,35,36].

Comparator selection matters because polymeric systems are often more complex than conventional formulations. A nanocarrier should show added value in exposure, release control, tissue deposition, safety, dosing convenience, or biological performance. If it only improves an in vitro solubility number, its translational value remains uncertain [5,7,8,9,14,16,18].

Go/no-go criteria should also be defined earlier. Examples include minimum drug loading, acceptable release window, absence of drug crystallization after storage, reproducible batch quality, acceptable local tolerability, measurable tissue deposition, and superiority or clear advantage over simpler formulations [5,7,8,9,12,14,15,16,18]. These criteria would help move OA delivery research from formulation screening toward product-oriented development.

### 13.9. Staged Roadmap for Future OA Polymeric Systems

A staged roadmap may help organize future work. The first stage should define the target product profile: indication, route, target tissue, dosing logic, comparator, and intended clinical advantage. The second stage should select the polymeric or polymer-assisted system according to that profile. The third stage should establish critical quality attributes, including OA physical state, loading, release, stability, sterility or microbial quality where relevant, and route-specific performance.

The fourth stage should use disease-relevant models and exposure verification. At this point, studies should show not only that OA is released, but that it reaches the relevant tissue or cellular compartment and produces a measurable biological response. The fifth stage should address manufacturing, scale-up, storage, packaging, safety, and comparator performance.

Only systems that pass these stages should move toward more advanced animal models or early clinical feasibility studies. This staged approach avoids overclaiming while still providing a clear development path for OA polymeric nanocarriers [8,12,14,15,16,18,25,32,35,36,37,38,39,44].

### 13.10. Clinical Prioritization Roadmap

A practical roadmap for OA polymeric nanocarriers should prioritize applications with the strongest combination of biological rationale, delivery feasibility, and clinical relevance. Based on current evidence, local dermatological delivery, wound-oriented systems, and intra-articular osteoarthritis depots appear more immediately realistic than systemic cancer or metabolic-disease applications.

In the first stage, formulation studies should focus on robust polymeric design and characterization. This includes defining polymer role, OA physical state, release kinetics, stability, and appropriate controls. In the second stage, disease-relevant biological models should be used to confirm that the release profile produces meaningful tissue response [8,12,14,15,16,18,32,36,38,39].

In the third stage, translational formulation attributes should be evaluated, including sterilization, scalability, shelf-life, dose uniformity, local tolerability, and comparison with standard therapies [13,15,16,25,26,32,36,44]. In the fourth stage, selected formulations could be advanced into more predictive animal models and, eventually, early clinical feasibility studies if safety and efficacy signals justify progression.

This staged roadmap avoids overclaiming while still identifying a clear path forward [8,12,13,14,15,16,18,25,26,32,36,38,39,44]. OA polymeric nanocarriers are promising, but their future depends on disciplined formulation development, not only on repeated demonstration of improved solubility or in vitro activity [1,5,7,8,9,12,14,16,18,32].

### 13.11. Research Roadmap

Future progress in OA polymeric nanocarriers and polymer-assisted delivery platforms will depend on moving from formulation feasibility to disease-specific development. The field should prioritize local and tissue-targeted systems, physical-state control, route-relevant release testing, exposure verification, disease-relevant models, QbD-based development, manufacturing-aware innovation, and meaningful comparators [8,13,14,15,16,17,18,25,32,35,36,38,39,44].

The most realistic early opportunities are likely to involve skin inflammation, psoriasis, wound healing, dermal delivery, osteoarthritis, and related localized inflammatory conditions. These applications fit the strengths of polymeric delivery because they can benefit from sustained local exposure, controlled release, tissue retention, and reduced systemic burden [8,12,14,15,16,18,32,35,36,38,39].

The long-term goal should be to establish a process–structure–function framework for OA delivery. This means connecting polymer selection and manufacturing conditions with carrier architecture, OA physical state, release kinetics, tissue exposure, biological response, safety, and patient-relevant performance. For OA, the best future formulations will not be the most complex systems, but those that solve a clearly defined delivery problem better than simpler alternatives.

**Figure 7:**
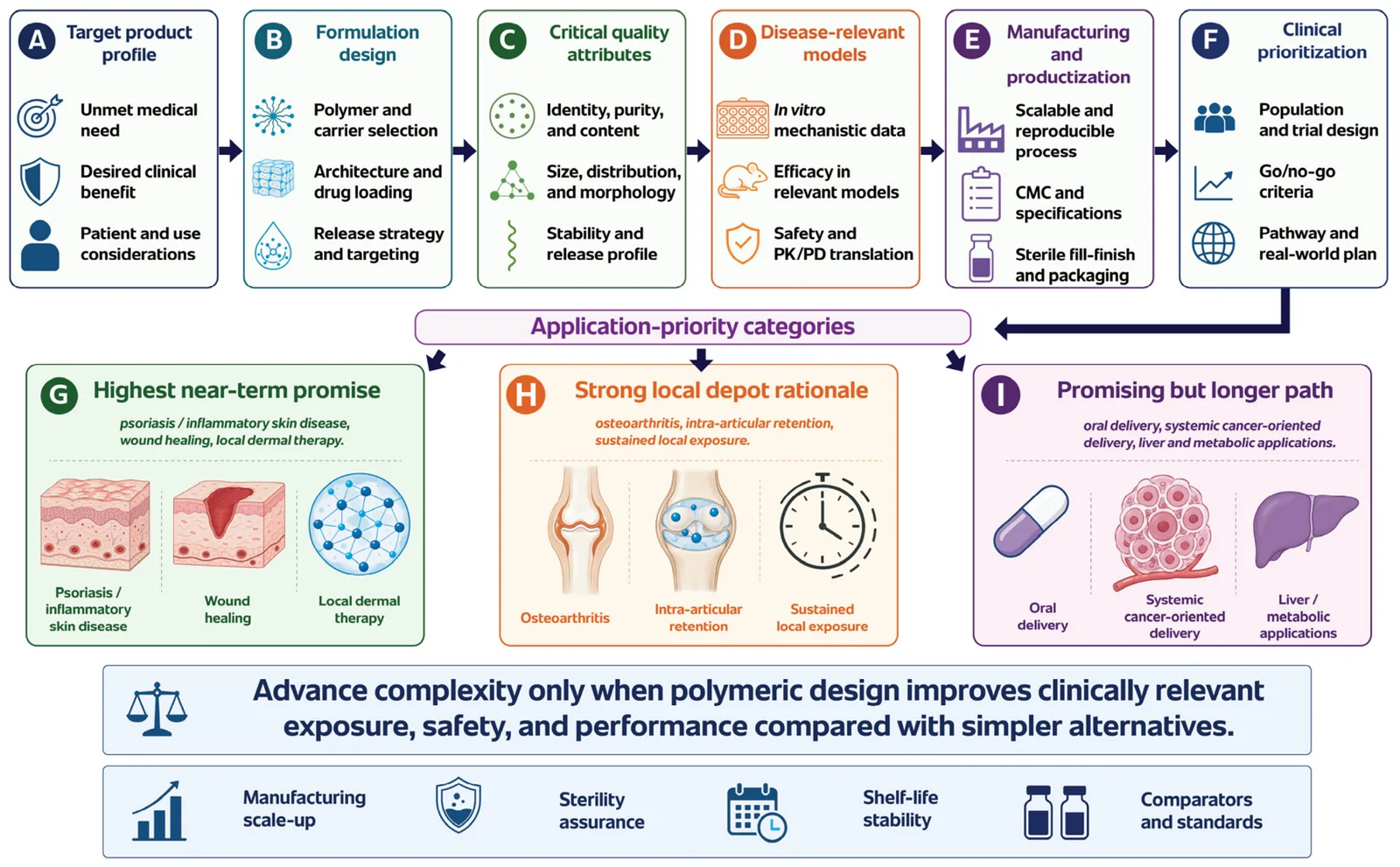
Translational roadmap for OA-loaded polymeric nanocarriers and polymer-assisted delivery platforms. Panels (**A**–**F**) present the sequential development pathway: (**A**) definition of the target product profile and unmet medical need; (**B**) formulation design and carrier selection; (**C**) definition of critical quality attributes; (**D**) evaluation in disease-relevant models; (**E**) manufacturing and productization; and (**F**) clinical prioritization. The arrows between panels A–F indicate the sequence of development. Panels (**G**–**I**) represent three parallel application-priority categories resulting from clinical prioritization: (**G**) applications with the highest near-term promise, including psoriasis, inflammatory skin disease, wound healing, and local dermal therapy; (**H**) applications with a strong local-depot rationale, particularly osteoarthritis, intra-articular retention, and sustained local exposure; and (**I**) promising applications requiring a longer development path, including oral delivery, systemic cancer-oriented delivery, and liver or metabolic applications. The lower elements indicate cross-cutting development requirements related to manufacturing scale-up, sterility assurance, shelf-life stability, and comparison with relevant standards and simpler alternatives.

A staged translational roadmap for OA-loaded polymeric nanocarriers and polymer-assisted delivery platforms is shown in Figure 7.

**Figure 8:**
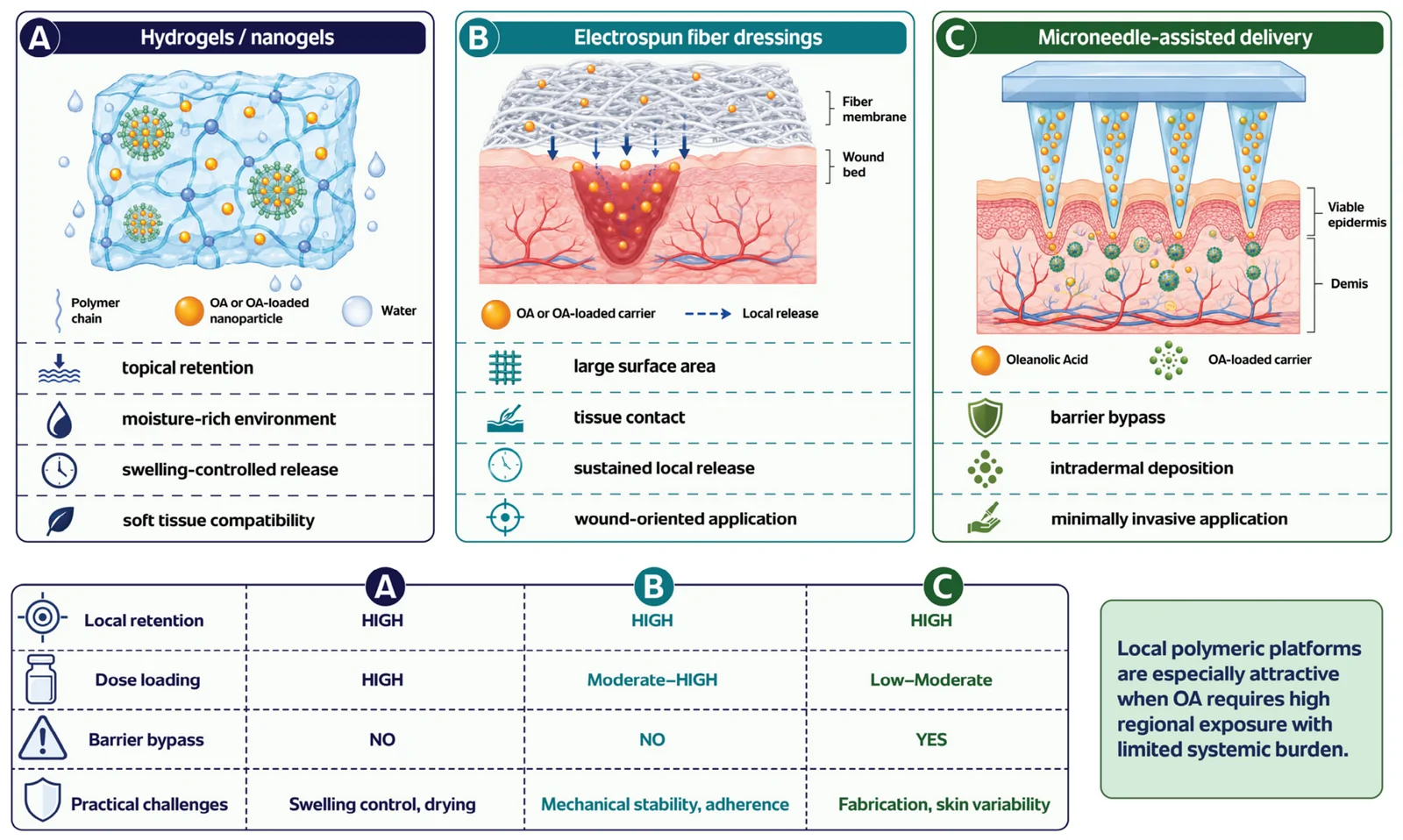
Local matrix-based polymeric delivery platforms for OA: hydrogels, fiber dressings, and microneedles. (**A**) Hydrogel and nanogel matrices providing topical retention, a moisture-rich environment, swelling-controlled release, and soft-tissue compatibility. (**B**) Electrospun fiber dressings providing a large surface area, tissue contact, sustained local release, and wound-oriented application. (**C**) Microneedle-assisted delivery providing skin-barrier bypass, intradermal deposition, and minimally invasive administration. The lower comparison matrix refers to the same platforms shown in panels (**A**–**C**) and summarizes their relative local retention, dose-loading capacity, barrier-bypass capability, and principal practical challenges.

A narrower comparison of local matrix-based platforms, including hydrogels, fiber dressings, and microneedle-assisted delivery, is shown in Figure 8.

Research priorities and decision criteria for future OA polymeric delivery are summarized in Table 6.

## 14. Conclusions

Oleanolic acid is a biologically attractive but formulation-limited pentacyclic triterpenoid. Poor aqueous solubility, crystallinity, slow dissolution, limited barrier transport, low and variable bioavailability, and strong vehicle dependence continue to restrict its biomedical development [1,5]. These properties make OA a useful example of a compound for which pharmacological performance cannot be interpreted independently of formulation and delivery.

Polymeric nanocarriers and polymer-assisted delivery platforms address different parts of this problem. PLA/PLGA nanoparticles, PEGylated nanoparticles, polymeric micelles, and hyaluronic-acid-based nanoprodrugs can improve OA dispersion, modify its physical state, regulate release, or alter cellular and tissue interaction. Hydrogels, fiber membranes, thermosensitive depots, wound dressings, and microneedle systems provide additional route-enabling functions, including local retention, tissue contact, mechanical administration, and barrier bypass [7,8,9,12,14,15,16,18,32]. The value of these systems lies not only in improved apparent solubility, but in their ability to define exposure more precisely.

The field should now move beyond proof of loading or formulation feasibility. Future OA delivery studies need to connect polymer composition, carrier architecture, drug physical state, release kinetics, tissue deposition, biological response, safety, and manufacturing reproducibility. This process–structure–function logic is essential for distinguishing useful delivery systems from formulations that are technically interesting but not developmentally persuasive [7,8,9,12,14,15,18].

The quantitative evidence reviewed here shows that carrier size, drug loading, encapsulation efficiency, and apparent solubility do not independently predict biological performance. High encapsulation efficiency may coexist with slow or incomplete release, whereas lower loading may still produce greater cellular exposure or a stronger biological response. Similarly, improved in vitro dispersion does not by itself demonstrate increased systemic bioavailability or therapeutic efficacy. The most informative studies are those that connect physicochemical characterization with release, target-compartment exposure, application-relevant biological response, and safety.

No single polymeric platform can currently be identified as universally superior for OA. The reported studies differ in carrier composition, analytical methods, release media, administered doses, biological models, and endpoints, which prevents direct quantitative ranking or formal comparison across all systems. Formulation selection should therefore begin with the intended route, target tissue, required exposure duration, safety requirements, and clinically relevant comparator rather than with the aim of maximizing particle-related parameters alone.

Local and tissue-targeted applications currently appear to provide the strongest near-term rationale. Inflammatory skin disease, psoriasis, wound healing, dermal delivery, and osteoarthritis can benefit from controlled release and prolonged local residence without requiring maximal systemic exposure [8,12,14,15,16,18]. Cancer, liver injury, metabolic disorders, and oral bioavailability enhancement remain scientifically relevant, but require stronger pharmacokinetic, biodistribution, target-tissue exposure, repeated-dose safety, and comparative-efficacy evidence before translational claims can be made [5,7,9,19,20,38,39,43,47].

Manufacturing and product-development considerations should be integrated from the earliest stages. OA physical state, raw-material properties, polymer specifications, solvent history, process parameters, sterilization or microbial control, drying, storage, packaging, batch reproducibility, and route-specific critical quality attributes may all determine performance. More complex systems, including responsive nanoprodrugs and nanocarrier-loaded microneedles, require correspondingly stronger evidence that their additional development burden produces a meaningful therapeutic advantage. The most promising OA formulation will therefore not necessarily be the system with the highest loading, smallest particle size, or most complex architecture. It will be the system that provides reproducible and safe OA exposure at the intended site, can be manufactured and stored reliably, and performs measurably better than an appropriate simpler formulation or current standard of care.

## Figures and Tables

**Table 1:** Formulation-relevant barriers of oleanolic acid and their implications for polymeric delivery.

Barrier/Property [Reference]	Relevance for OA Delivery	Implication for Polymeric or Polymer-Assisted Carrier Design
Hydrophobic pentacyclic triterpenoid structure [1,2,3]	OA contains a rigid hydrophobic scaffold with limited polar functionality, which reduces aqueous compatibility.	Supports incorporation into hydrophobic polymer matrices, micellar cores, lipid–polymer interfaces, and nanogel domains.
Poor aqueous solubility [1,5]	Low solubility limits dissolution, biological availability, and reproducible in vitro exposure.	Polymeric nanoparticles, micelles, cyclodextrin-containing systems, and nanogels can improve apparent dispersion and handling.
Crystallinity and solid-state behavior [5,7,9]	Crystalline OA may dissolve slowly and show variable release.	Solid-state characterization should be included to determine whether OA is crystalline, amorphous, molecularly dispersed, or phase-separated.
Low and variable bioavailability [1,5,12,14]	Oral and systemic exposure are limited by dissolution, permeability, and formulation-dependent absorption.	Polymeric systems may improve exposure, but pharmacokinetic and tissue-distribution studies remain necessary.
Limited permeability and barrier transport [5,8,15,18]	Lipophilicity alone does not ensure efficient skin, epithelial, or tissue penetration.	Nanocarriers, hydrogels, local depots, and microneedles may improve tissue deposition or bypass biological barriers.
Vehicle-dependent biological response [5,17]	OA activity depends strongly on the vehicle, solubilizer, carrier, or matrix used for delivery.	Blank carriers, free OA, solubilized OA, and OA-loaded carrier controls are essential for biological interpretation.

**Table 2:** Quantitative comparison of representative OA-loaded polymeric nanocarriers and polymer-assisted delivery platforms.

Delivery System [Reference]	Particle or Matrix Characteristics	OA Loading and Encapsulation	Release, Stability, or Local Exposure	Experimental Model	Quantitative Biological or Formulation Outcome
OA-loaded PEGylated PLA/PLGA nanoparticles [7]	Diameter: 201.5 ± 0.72 to 250.2 ± 25.39 nm; PDI: 0.20 ± 0.07 to 0.27 ± 0.03; ζ-potential: −6.66 ± 0.75 to −20.1 ± 1.62 mV	DL: 4.08 ± 0.30 to 7.58 ± 0.92%; EE: 40.8 ± 2.97 to 75.8 ± 9.17%	Physically stable for at least 20 weeks at 4 and 25 °C; enabled aqueous administration of OA at concentrations up to 100 µg/mL	A549 and HepG2 cancer cells; BEAS-2B non-cancer cells	All four OA-loaded nanoparticle formulations reduced A549 viability below 50% after 48 h; mPEG–PLGA systems showed stronger cancer-cell cytotoxicity despite lower EE than mPEG–PLA systems
OA/UA-loaded PLGA nanoparticles [9]	Diameter: 213.55 ± 1.60 and 217.98 ± 2.74 nm; PDI: 0.090 ± 0.038 and 0.073 ± 0.011; ζ-potential: −27.12 ± 0.27 and −26.85 ± 0.49 mV	EE: 79.01 ± 4.22 and 78.25 ± 3.01%	Approximately 75% release by 72 h; physical stability for 6 months	HepG2, Caco-2, and Y-79 cells	In Y-79 cells, 48 h IC50 values were 19.1 ± 2.2 and 11.1 ± 0.8 µmol/L for nanoparticle formulations versus 2.6 ± 0.4 and 2.9 ± 0.3 µmol/L for the corresponding free OA/UA mixtures
OA polymeric micelles [14]	Diameter: 80.4 ± 11.1 nm for PMO-G and 57.0 ± 5.24 nm for PMO-H	EE: 98.26 ± 0.17% for PMO-G and 99.18 ± 1.06% for PMO-H	After 24 h, PMO-H produced 56.22 ± 13.50% OA retention in skin and 29.49 ± 4.00% permeation; OA content remained above 98% and particle size remained 49.6 ± 5 nm after 3 months at 40 °C/75% RH	Ex vivo hairless-mouse skin; human irritation study; 8-week split-face clinical study	In 20 participants, mean skin-roughness R3 decreased from 0.094 ± 0.023 to 0.086 ± 0.020 after 8 weeks; no irritation was observed in 25 volunteers
Reduction-responsive HA–OA nanoprodrug nanoparticles with additional encapsulated OA [12]	Diameter: 75.18 ± 3.24 nm; PDI: 0.20 ± 0.01; ζ-potential: approximately −27 mV; CMC: 30.85 ± 1.95 µg/mL	OA grafting degree: 15%; total drug loading: up to 35%	Approximately 90% cumulative release within 72 h in the presence of 10 mM GSH; particle size and ζ-potential remained stable over 15 days	HaCaT keratinocytes; IMQ-induced psoriasis model in mice	Free-OA IC50 values were 267.43 ± 21.3 and 82.59 ± 13.12 µg/mL after 24 and 48 h, respectively; OA-NPs@OA values were 7.49 ± 0.41 and 4.25 ± 0.50 µg/mL. Topical treatment produced dose-dependent improvement and was more effective than free OA and calcipotriol
OA-loaded cubic liquid crystalline nanoparticles incorporated into topical gels [15]	Diameter: 129 ± 12.11 to 272 ± 21.83 nm; PDI: 0.218–0.436; ζ-potential: −18.3 to −21.2 mV	DEE: 68.31 ± 2.86 to 73.18 ± 3.21%; DL: 12.31 ± 0.41 to 14.12 ± 0.32%	Gel formulations released 84.93–87.89% of OA within 12 h; cumulative ex vivo permeation was 371.5–429.8 µg/cm^2^ after 12 h	Ex vivo skin permeation; carrageenan-induced rat hind-paw edema	The optimized OA-LCNP gel produced 45.26% edema inhibition at 24 h after a single application, compared with 25.28% edema in the test group and 40.92% in the untreated control
OA-loaded PLGA fiber membranes [8]	Mean fiber diameter: 542 ± 165 nm for PLGAOA20 and 630 ± 192 nm for PLGAOA33	Actual DL: 16.34 and 27.25%; drug-loading efficiency: 81.7 and 82.57%	Release after 1 day: 8.56 and 10.27%; cumulative release after 14 days: 28.27 and 27.96%	NIH 3T3 mouse fibroblasts	Both fiber formulations supported fibroblast adhesion and proliferation and showed non-cytotoxic behavior; slow release was attributed to the hydrophobic PLGA–OA matrix and polymer degradation
OA cubic liquid crystalline nanoparticle-based thermosensitive gel [18]	Thermosensitive gelation at approximately 34 °C; release described by the Higuchi model; nanoparticle size and EE were not re-reported in the therapeutic study	NR in the therapeutic study; formulation was prepared from OA-LCNP dispersion using Poloxamers F127 and F68	Sustained intra-articular release and joint retention; complete quantitative release profile referred to earlier formulation-development work	Papain-induced knee osteoarthritis in rats; *n* = 10 per group	Intra-articular doses of 25 and 50 mg/kg significantly improved locomotor and pain-related outcomes and reduced inflammatory and cartilage-degradation markers compared with the untreated model group

Abbreviations: CMC, critical micelle concentration; DEE, drug entrapment efficiency; DL, drug loading; EE, encapsulation efficiency; GSH, glutathione; HA, hyaluronic acid; IC50, half-maximal inhibitory concentration; IMQ, imiquimod; NR, not reported; OA, oleanolic acid; PDI, polydispersity index; PLA, poly(lactic acid); PLGA, poly(lactic-co-glycolic acid); UA, ursolic acid; ζ-potential, zeta potential. Values are presented as reported in the original studies. Direct comparisons should be interpreted cautiously because formulations differed in composition, analytical methods, release media, administered doses, and biological models.

**Table 3:** Functional roles, advantages, limitations and representative applications of polymeric materials and polymer-assisted platforms used in OA delivery.

Polymeric Material/Platform	Functional Role in OA Delivery	Advantages	Main Limitations or Risks	Most Relevant Applications	References
PLGA/PLA	Biodegradable hydrophobic matrix for nanoparticle or fiber-based delivery.	Sustained release, hydrophobic drug accommodation, established use in drug delivery research.	Residual solvent, acidic degradation products, crystallization risk, incomplete release.	Cancer models, local membranes, sustained-release systems.	[7,8,9,13,25]
PEGylated polymers	Steric stabilization and improved colloidal dispersion.	Reduced aggregation, improved hydration, altered protein and cell interactions.	Possible reduced cellular uptake; PEG density and surface architecture require control.	PEGylated PLA/PLGA nanoparticles, PEGylated hybrid systems.	[7,26]
Hyaluronic acid	Biointeractive polymeric component and possible receptor-mediated delivery element.	Biocompatibility, disease-oriented interaction, potential CD44-related uptake.	Molecular-weight dependence, conjugation variability, degradation control.	Psoriasis and inflammatory skin disease.	[12,27]
Poloxamers and thermosensitive polymers	Gelation and local depot formation.	Injectable or topical gel formation, sustained local residence.	Concentration-dependent gelation, dilution sensitivity, local tolerability.	Intra-articular depots, topical gels, thermosensitive systems.	[18,28]
Cyclodextrin-containing systems	Inclusion complexation and solubility enhancement.	Improved OA handling and exposure standardization.	Complex dissociation; limited sustained release unless incorporated into polymeric networks.	Solubility enhancement, topical matrices, biological assay standardization.	[17,29,30]
Polymeric micelle-forming copolymers	Hydrophobic core formation and aqueous dispersion.	Improved solubilization of hydrophobic OA; dermal delivery potential.	Dilution instability, premature release, micelle dissociation.	Skin delivery and topical formulations.	[14,31]
Hydrogel- and microneedle-forming polymers	Local retention, controlled release, and skin-barrier bypass.	Tissue residence, minimally invasive delivery, local exposure.	Mechanical strength, dose uniformity, drying stability, irritation risk.	Wounds, inflammatory skin disease, intradermal delivery.	[15,18,32,33]

**Table 4:** Local, transdermal, and microneedle-compatible OA delivery platforms based on polymeric or polymer-assisted systems.

Platform	Main Formulation Logic	Potential Clinical Relevance	Key Evaluation Parameters	References
Topical polymeric micelles	OA solubilization in amphiphilic polymeric assemblies.	Dermal delivery, skin-compatible delivery, possible inflammatory skin applications.	Micelle size, dilution stability, skin permeation, deposition, irritation.	[14]
HA-based OA nanoparticles	Disease-oriented polymeric nanoprodrug and responsive delivery.	Psoriasis and inflammatory skin disease.	Uptake, skin deposition, cytokine response, irritation, repeated-dose safety.	[12]
OA-loaded topical gel	Nanocarrier-in-gel local delivery.	Local anti-inflammatory topical therapy.	Rheology, release, ex vivo permeation, anti-inflammatory response.	[15]
OA-loaded thermosensitive nanogel	Injectable local depot with temperature-triggered gelation.	Knee osteoarthritis and intra-articular therapy.	Gelation temperature, injectability, joint residence, cartilage protection, synovial inflammation.	[18]
OA-loaded PLGA fiber membrane	Biodegradable tissue-contacting polymeric matrix.	Wound dressing, topical patch, local depot.	Fiber morphology, mechanical strength, drug distribution, release, tissue compatibility.	[8]
Nanocarrier-loaded microneedles	Skin-barrier bypass combined with polymeric or nanocarrier-mediated release.	Inflammatory skin disease and intradermal OA delivery.	Needle strength, insertion, dissolution/swelling, OA deposition, carrier stability.	[32,33]
Polymeric wound dressing	Moisture-regulating matrix with local OA release.	Wound healing and tissue repair.	Exudate handling, migration assays, cytokines, oxidative stress, wound closure.	[8,38,39]

**Table 5:** Platform- and route-specific critical quality attributes for OA-loaded polymeric nanocarriers and polymer-assisted delivery platforms.

Platform/ Intended Route [References]	Primary Critical Quality Attributes	OA-Specific Rationale and Main Failure Risks	Recommended Characterization
Attributes common to all OA polymeric systems [1,5,7,8,9,13]	OA identity and content; impurity profile; drug loading; encapsulation efficiency; physical state; morphology; release; stability; batch reproducibility	Nominal OA content may include molecularly dispersed, amorphous, crystalline, surface-associated, or precipitated fractions. Changes in physical state may alter release and biological exposure without changing total drug content.	Validated HPLC or LC-MS; recovery and mass-balance studies; DSC; PXRD; FTIR or Raman spectroscopy; microscopy; release testing; stability-indicating assays
Oral nanoparticles, micelles, or polymer-assisted systems [1,5,19,20]	Dispersion after gastrointestinal dilution; precipitation risk; particle size and PDI; release in biorelevant media; interaction with bile salts, digestive components, and food; chemical stability	A formulation may initially disperse OA but lose performance after dilution, digestion, or pH change. High apparent solubility does not guarantee absorption if OA precipitates or remains strongly carrier-associated.	Simulated gastric and intestinal media; dilution and precipitation studies; lipolysis where relevant; dissolution/release testing; permeability models; pharmacokinetics
Systemic or intravenous nanoparticles [7,9,13,25,43]	Sterility; endotoxin burden; particle size and PDI; serum stability; protein interaction; aggregation; free OA fraction; release; biodistribution; systemic safety	Aggregation, protein-corona formation, premature release, or excessive carrier retention may alter circulation and tissue distribution. Particle size and EE alone are insufficient predictors of systemic performance.	DLS and imaging before and after serum exposure; sterility and endotoxin assays; protein-binding studies; release in protein-containing media; pharmacokinetics; biodistribution; hematological and histological safety
Topical or dermal micelles, nanoparticles, and gels [12,14,15]	OA content and uniformity; rheology; spreadability; skin deposition; permeation; local release; microbial quality; irritation; barrier compatibility	Increased permeation is not always desirable if the therapeutic goal is epidermal or dermal retention. OA crystallization or phase separation may reduce dose uniformity and local availability.	Rheology; Franz diffusion cells; skin-layer extraction; ex vivo or reconstructed-skin models; microscopy; irritation and barrier-integrity testing; microbial-limit testing
Wound dressings and polymeric fiber membranes [8,35,36,37,38]	Drug distribution; fiber or matrix morphology; porosity; thickness; tensile properties; flexibility; adhesion; moisture and exudate management; release; microbial control; damaged-tissue compatibility	Wound exudate, proteins, enzymes, pH variation, and infection can alter OA release and matrix integrity. Nonuniform OA distribution may produce variable local dosing.	SEM; mechanical testing; porosity and swelling analysis; release in wound-exudate-like media; adhesion testing; microbial testing; migration, inflammation, tissue-repair, and histological models
Intra-articular thermosensitive gels and depots [15,18]	Sterility and endotoxin control; injectability; viscosity; gelation temperature and time; OA dose uniformity; local release; joint residence; cartilage and synovial compatibility	A formulation must remain injectable before administration but form or maintain a stable depot under joint conditions. Excessive retention, rapid clearance, or OA crystallization may reduce safety or efficacy.	Rheology; syringeability and injection-force testing; gelation studies; release in synovial-fluid-like media; imaging or tissue quantification of joint residence; cartilage, synovial, and repeated-dose safety
Dissolving or hydrogel-forming microneedles [16,32,33]	Needle geometry; mechanical strength; insertion efficiency; OA dose uniformity; residual moisture; dissolution or swelling; carrier stability after drying; skin deposition; packaging stability	Drying and concentration may induce OA crystallization or destabilize an embedded nanocarrier. Adequate drug loading is irrelevant if needles fail to insert or deliver a uniform dose.	Optical or electron microscopy; compression and fracture testing; insertion models; dissolution/swelling testing; OA assay per patch; residual-moisture analysis; skin-deposition and irritation studies

Abbreviations: DSC, differential scanning calorimetry; EE, encapsulation efficiency; FTIR, Fourier-transform infrared spectroscopy; HPLC, high-performance liquid chromatography; LC-MS, liquid chromatography–mass spectrometry; OA, oleanolic acid; PDI, polydispersity index; PXRD, powder X-ray diffraction; SEM, scanning electron microscopy. The listed attributes represent development priorities rather than universal acceptance criteria. Appropriate specifications should be defined according to the intended route, dosage form, target tissue, and relationship between product attributes and biological performance.

**Table 6:** Research priorities and translational roadmap for OA-loaded polymeric nanocarriers and polymer-assisted delivery platforms.

Research Priority	Current Limitation	Recommended Direction	Expected Impact
Disease-specific design	Many systems are optimized for loading rather than clinical use.	Define a target product profile for psoriasis, wound healing, osteoarthritis, cancer, or oral delivery before carrier design.	Stronger translational relevance.
Physical-state control	OA crystallinity and amorphous/crystalline transitions are often underexplored.	Include DSC, PXRD, Raman/FTIR, microscopy, and stability monitoring.	More reliable release and shelf-life prediction.
Route-relevant release testing	Simple buffer release may not reflect biological conditions.	Use skin-, wound-, synovial-, gastrointestinal-, or protein-containing media depending on application.	More meaningful release interpretation.
Exposure verification	Nominal OA dose may not equal biologically available OA.	Quantify OA in media, cells, skin layers, tissue, or local compartments.	Better link between formulation and biological response.
Comparative benchmarking	New formulations are often compared only with free OA.	Compare polymeric systems with cyclodextrin complexes, micelles, gels, SEDDS/SNEDDS, and standard therapies when appropriate.	Clarifies whether formulation complexity is justified.
Manufacturing-aware development	Many systems are difficult to sterilize, store, or scale.	Consider residual solvent, sterility, drying, packaging, process robustness, QbD, and DoE early.	Higher product-development feasibility.
Clinical prioritization	OA applications are broad but not equally mature.	Prioritize local skin, wound, and osteoarthritis delivery before more demanding systemic indications.	More realistic translational pathway.

## Data Availability

No new data were created or analyzed in this study. Data sharing is not applicable to this article.

## Supplementary Materials

The following supporting information can be downloaded at: https://www.mdpi.com/article/10.3390/mi17080944/s1, Table S1: Controlled-release mechanisms relevant to OA polymeric and polymer-assisted systems; Table S2: Translational positioning of OA polymeric and polymer-assisted systems by application area. Reference [52] is cited in Supplementary Materials.

## Abbreviations

The following abbreviations are used in this manuscript:

CQA	Critical quality attribute
DSC	Differential scanning calorimetry
FTIR	Fourier-transform infrared spectroscopy
GMP	Good manufacturing practice
HA	Hyaluronic acid
HPLC	High-performance liquid chromatography
LC–MS	Liquid chromatography–mass spectrometry
OA	Oleanolic acid
PEG	Polyethylene glycol
PLA	Poly(lactic acid)
PLGA	Poly(lactic-co-glycolic acid)
PDI	Polydispersity index
PXRD	Powder X-ray diffraction
QbD	Quality by design
QTPP	Quality target product profile
