# Supplementary material for: Polymeric Nanocarriers and Polymer-Assisted Delivery Platforms for Oleanolic Acid: Design Strategies, Controlled Release, Translational Challenges, and Clinical Perspectives
Source: Micromachines (Basel). 2026 Aug 7;17(8):944. doi: 10.3390/mi17080944 (PMC13515084; doi:10.3390/mi17080944)
Supplement: Supplementary file 1 [file micromachines-17-00944-s001.zip › micromachines-4442419-supplementary.pdf]

micromachines

Review

Polymeric Nanocarriers and Polymer-Assisted Delivery Platforms for Oleanolic Acid: Design Strategies, Controlled Release, Translational Challenges, and Clinical Perspectives

Andrzej Günther [1,*] and Barbara Bednarczyk-Cwynar [1,2]

1 Department of Organic Chemistry, Faculty of Pharmacy, Poznan University of Medical Sciences, Collegium Pharmaceuticum 2 (CP.2), Rokietnicka Str. 3, 60-806 Poznan, Poland; bcwynar@ump.edu.pl
2 Center of Innovative Pharmaceutical Technology (CITF), Rokietnicka Str. 3, 60-806 Poznan, Poland
* Correspondence: andrzej.gunther@me.pl

 https://doi.org/10.3390/mi17080944

Table S1. Controlled-release mechanisms relevant to OA polymeric and polymer-assisted systems.

System type	Main release-control mechanism	OA-specific concerns	Suitable applications	References
PLGA / PLA nanoparticles	Diffusion, polymer hydration, matrix relaxation, hydrolytic degradation, erosion.	OA may remain trapped in the hydrophobic matrix or crystallize at high loading.	Sustained systemic or local delivery, cancer models.	[1–5]
PEGylated polymeric nanoparticles	Matrix release combined with steric surface effects.	PEG may improve stability but alter uptake and tissue interaction.	Cancer-oriented delivery, systemic nanocarriers.	[1,6]
Polymeric micelles	Diffusion from micellar core, micelle dissociation, dilution-triggered release.	Micelles may dissociate after dilution; OA may precipitate or partition into tissue.	Dermal and topical delivery.	[7,8]
HA-based OA nanoprodrugs	Polymer–drug cleavage, redox-responsive release, receptor-mediated uptake.	Requires control of conjugation degree, free OA, linker stability, and degradation behavior.	Psoriasis and inflammatory skin disease.	[9,10]
Cyclodextrin complexes / cyclodextrin-polymer systems	Inclusion complex dissociation; network diffusion if polymerized.	Complexation improves solubility but may not ensure sustained release.	Exposure standardization, topical matrices, soft polymeric systems.	[11–13]
Hydrogels and nanogels	Swelling, diffusion, erosion, network relaxation, carrier-in-gel release.	Direct OA loading may be poor without a nanocarrier or solubilizing component.	Wounds, topical delivery, local depots.	[14–16]
Thermosensitive gels	Temperature-triggered gelation and diffusion from depot.	Gelation temperature, injectability, and residence time are critical.	Intra-articular osteoarthritis delivery.	[15,16]
Polymeric fiber membranes	Diffusion from fibers, polymer degradation, surface-area-dependent release.	Fiber processing may alter OA distribution and crystallinity.	Wound dressings, local tissue-contacting platforms.	[17]
Nanocarrier-loaded microneedles	Microneedle dissolution or swelling followed by nanocarrier-level release.	Drying may destabilize carriers or induce OA crystallization.	Skin inflammation, intradermal delivery.	[18,19]

https://doi.org/10.3390/mi17080944

Table S2. Translational positioning of OA polymeric and polymer-assisted systems by application area.

Application area	Why OA is relevant	Most suitable polymeric / polymer-assisted systems	Main translational barriers	References
Inflammatory skin diseases / psoriasis	OA has anti-inflammatory potential, and topical delivery may reduce systemic burden.	HA-based nanoparticles, polymeric micelles, hydrogels, microneedles.	Skin irritation, barrier recovery, repeated-dose safety, comparison with standard therapies.	[7,9,18–20]
Wound healing	OA has reported wound-healing and cell-migration-related effects.	PLGA fiber membranes, hydrogels, nanocarrier-loaded dressings.	Stage-specific wound biology, infection risk, wound exudate effects, realistic wound models.	[17,21,22]
Osteoarthritis	Local inflammation and cartilage degeneration may benefit from sustained intra-articular exposure.	Thermosensitive nanogels, injectable depots, polymer-assisted local carriers.	Joint residence, repeated-dose safety, cartilage compatibility, comparison with intra-articular standards.	[15]
Cancer nanomedicine	OA shows preclinical anticancer activity and polymeric carriers can alter cellular exposure.	PEGylated PLA/PLGA nanoparticles, PLGA nanoparticles.	Tumor penetration, biodistribution, pharmacokinetics, safety, clinical comparators.	[2,23,24]
Liver injury / hepatoprotection	OA has reported hepatoprotective and Nrf2-related effects.	Oral or systemic polymeric nanoparticles, polymer-assisted solubilizing systems.	Liver targeting, systemic exposure, safety, pharmacokinetics.	[20,25]
Oral bioavailability enhancement	OA has low oral bioavailability and poor dissolution.	Polymeric nanoparticles, micelles, cyclodextrin-polymer systems.	GI dilution, mucus, food effects, sink conditions, comparison with SEDDS/SNEDDS.	[11,20,26–28]
Cosmetic / dermal aging	OA has been evaluated in polymeric micelles for anti-wrinkle efficacy.	Polymeric micelles, topical gels.	Cosmetic vs therapeutic claims, skin deposition, long-term safety.	[7]

https://doi.org/10.3390/mi17080944

References

1. Man, D.K.W.; Casettari, L.; Cespi, M.; Bonacucina, G.; Palmieri, G.F.; Sze, S.C.W.; Leung, G.P.H.; Lam, J.K.W.; Kwok, P.C.L. Oleanolic Acid Loaded PEGylated PLA and PLGA Nanoparticles with Enhanced Cytotoxic Activity against Cancer Cells. *Mol. Pharm.* **2015**, *12*, 2112–2125, doi:10.1021/acs.molpharmaceut.5b00085.
2. Silva, A.M.; Alvarado, H.L.; Abrego, G.; Martins-Gomes, C.; Garduño-Ramirez, M.L.; García, M.L.; Calpena, A.C.; Souto, E.B. In Vitro Cytotoxicity of Oleanolic/Ursolic Acids-Loaded in PLGA Nanoparticles in Different Cell Lines. *Pharmaceutics* **2019**, *11*, 362, doi:10.3390/pharmaceutics11080362.
3. Yang, J.; Zeng, H.; Luo, Y.; Chen, Y.; Wang, M.; Wu, C.; Hu, P. Recent Applications of PLGA in Drug Delivery Systems. *Polymers* **2024**, *16*, 2606, doi:10.3390/polym16182606.
4. Makadia, H.K.; Siegel, S.J. Poly Lactic-Co-Glycolic Acid (PLGA) as Biodegradable Controlled Drug Delivery Carrier. *Polymers* **2011**, *3*, 1377–1397, doi:10.3390/polym3031377.
5. Siepmann, J.; Siepmann, F. Release Mechanisms of PLGA-Based Drug Delivery Systems: A Review. *Int. J. Pharm. X* **2025**, *10*, 100440, doi:10.1016/j.ijpx.2025.100440.
6. Mitchell, M.J.; Billingsley, M.M.; Haley, R.M.; Wechsler, M.E.; Peppas, N.A.; Langer, R. Engineering Precision Nanoparticles for Drug Delivery. *Nat. Rev. Drug Discov.* **2021**, *20*, 101–124, doi:10.1038/s41573-020-0090-8.
7. An, J.Y.; Yang, H.S.; Park, N.R.; Koo, T.; Shin, B.; Lee, E.H.; Cho, S.H. Development of Polymeric Micelles of Oleanolic Acid and Evaluation of Their Clinical Efficacy. *Nanoscale Res. Lett.* **2020**, *15*, 133, doi:10.1186/s11671-020-03348-3.
8. Kedar, U.; Phutane, P.; Shidhaye, S.; Kadam, V. Advances in Polymeric Micelles for Drug Delivery and Tumor Targeting. *Nanomedicine Nanotechnol. Biol. Med.* **2010**, *6*, 714–729, doi:10.1016/j.nano.2010.05.005.
9. Han, Y.; Ren, J.; Su, Y.; Zhou, L.; Zhu, X.; Wang, Z.; Kong, F.; Li, G. Hyaluronic Acid-Based Reduction Responsive Nanoparticles for Improved Anti-Psoriasis Effects of Traditional Chinese Herb Monomer Oleanolic Acid via Blocking YAP-AREG Axis. *J. Nanobiotechnology* **2026**, *24*, 240, doi:10.1186/s12951-026-04264-x.
10. Cyphert, J.M.; Trempus, C.S.; Garantziotis, S. Size Matters: Molecular Weight Specificity of Hyaluronan Effects in Cell Biology. *Int. J. Cell Biol.* **2015**, *2015*, 1–8, doi:10.1155/2015/563818.
11. Stelling-Férez, J.; López-Miranda, S.; Gabaldón, J.A.; Nicolás, F.J. Oleanolic Acid Complexation with Cyclodextrins Improves Its Cell Bio-Availability and Biological Activities for Cell Migration. *Int. J. Mol. Sci.* **2023**, *24*, 14860, doi:10.3390/ijms241914860.
12. Real, D.A.; Bolaños, K.; Priotti, J.; Yutronic, N.; Kogan, M.J.; Sierpe, R.; Donoso-González, O. Cyclodextrin-Modified Nanomaterials for Drug Delivery: Classification and Advances in Controlled Release and Bioavailability. *Pharmaceutics* **2021**, *13*, 2131, doi:10.3390/pharmaceutics13122131.

13. Liu, Y.; Lin, T.; Cheng, C.; Wang, Q.; Lin, S.; Liu, C.; Han, X. Research Progress on Synthesis and Application of Cyclodextrin Polymers. *Molecules* **2021**, *26*, 1090, doi:10.3390/molecules26041090.
14. Shi, Z.; Pan, S.; Wang, L.; Li, S. Topical Gel Based Nanoparticles for the Controlled Release of Oleanolic Acid: Design and in Vivo Characterization of a Cubic Liquid Crystalline Anti-Inflammatory Drug. *BMC Complement. Med. Ther.* **2021**, *21*, 224, doi:10.1186/s12906-021-03399-8.
15. Shi, Z.; Jia, F.; Tang, X.; Li, Q. Oleanolic Acid Cubic Liquid Crystal Nanoparticle-Based Thermosensitive Gel Attenuates Knee Osteoarthritis Symptoms in Rats. *Front. Pharmacol.* **2025**, *16*, 1730566, doi:10.3389/fphar.2025.1730566.
16. Mauri, E.; Giannitelli, S.M.; Trombetta, M.; Rainer, A. Synthesis of Nanogels: Current Trends and Future Outlook. *Gels* **2021**, *7*, 36, doi:10.3390/gels7020036.
17. Ahmed, S.; Padilla-Gainza, V.M.; Gilkerson, R.; Narula, A.; Lozano, K. Processing-Structure–Property Relationships of Oleanolic Acid Loaded PLGA Fiber Membranes. *J. Mater. Sci.* **2023**, *58*, 4240–4255, doi:10.1007/s10853-023-08246-4.
18. Larrañeta, E.; Lutton, R.E.M.; Woolfson, A.D.; Donnelly, R.F. Microneedle Arrays as Transdermal and Intradermal Drug Delivery Systems: Materials Science, Manufacture and Commercial Development. *Mater. Sci. Eng. R Rep.* **2016**, *104*, 1–32, doi:10.1016/j.mser.2016.03.001.
19. Aldawood, F.K.; Andar, A.; Desai, S. A Comprehensive Review of Microneedles: Types, Materials, Processes, Characterizations and Applications. *Polymers* **2021**, *13*, 2815, doi:10.3390/polym13162815.
20. Castellano, J.M.; Ramos-Romero, S.; Perona, J.S. Oleanolic Acid: Extraction, Characterization and Biological Activity. *Multidiscip. Digit. Publ. Inst.* **2022**, *14*, 623–623, doi:10.3390/nu14030623.
21. Moura-Letts, G.; Villegas, L.F.; Marçalo, A.; Vaisberg, A.J.; Hammond, G.B. In Vivo Wound-Healing Activity of Oleanolic Acid Derived from the Acid Hydrolysis of *Anredera Diffusa*. *J. Nat. Prod.* **2006**, *69*, 978–979, doi:10.1021/np0601152.
22. Stelling-Férez, J.; Gabaldón, J.A.; Nicolás, F.J. Oleanolic Acid Stimulation of Cell Migration Involves a Biphasic Signaling Mechanism. *Sci. Rep.* **2022**, *12*, 15065, doi:10.1038/s41598-022-17553-w.
23. Shanmugam, M.K.; Dai, X.; Kumar, A.P.; Tan, B.K.H.; Sethi, G.; Bishayee, A. Oleanolic Acid and Its Synthetic Derivatives for the Prevention and Therapy of Cancer: Preclinical and Clinical Evidence. *Cancer Lett.* **2014**, *346*, 206–216, doi:10.1016/j.canlet.2014.01.016.
24. Fan, D.; Cao, Y.; Cao, M.; Wang, Y.; Cao, Y.; Gong, T. Nanomedicine in Cancer Therapy. *Signal Transduct. Target. Ther.* **2023**, *8*, 293, doi:10.1038/s41392-023-01536-y.
25. Reisman, S.A.; Aleksunes, L.M.; Klaassen, C.D. Oleanolic Acid Activates Nrf2 and Protects from Acetaminophen Hepatotoxicity via Nrf2-Dependent and Nrf2-Independent Processes. *Biochem. Pharmacol.* **2009**, *77*, 1273–1282, doi:10.1016/j.bcp.2008.12.028.

26. Wasim, M.; Bergonzi, M.C. Unlocking the Potential of Oleanolic Acid: Integrating Pharmacological Insights and Advancements in Delivery Systems. *Pharmaceutics* **2024**, *16*, 692, doi:10.3390/pharmaceutics16060692.
27. Xi, J.; Chang, Q.; Chan, C.K.; Meng, Z.Y.; Wang, G.N.; Sun, J.B.; Wang, Y.T.; Tong, H.H.Y.; Zheng, Y. Formulation Development and Bioavailability Evaluation of a Self-Nanoemulsified Drug Delivery System of Oleanolic Acid. *AAPS PharmSciTech* **2009**, *10*, 172–182, doi:10.1208/s12249-009-9190-9.
28. Su, L.; Yang; Huang; Dou; Zhai Self-Microemulsifying Drug Delivery System for Improved Oral Bioavailability of Oleanolic Acid: Design and Evaluation. *Int. J. Nanomedicine* **2013**, 2917, doi:10.2147/IJN.S47510.
